# On the Origins and Control of Community Types in the Human Microbiome

**DOI:** 10.1371/journal.pcbi.1004688

**Published:** 2016-02-11

**Authors:** Travis E. Gibson, Amir Bashan, Hong-Tai Cao, Scott T. Weiss, Yang-Yu Liu

**Affiliations:** 1 Channing Division of Network Medicine, Brigham and Women’s Hospital, Harvard Medical School, Boston, Massachusetts, United States of America; 2 Department of Electrical Engineering, University of Southern California, Los Angeles, California, United States of America; 3 Chu Kochen Honors College, College of Electrical Engineering, Zhejiang University, Hangzhou, Zhejiang, China; 4 Center for Cancer Systems Biology, Dana-Farber Cancer Institute, Boston, Massachusetts, United States of America; MIT, UNITED STATES

## Abstract

Microbiome-based stratification of healthy individuals into compositional categories, referred to as “enterotypes” or “community types”, holds promise for drastically improving personalized medicine. Despite this potential, the existence of community types and the degree of their distinctness have been highly debated. Here we adopted a dynamic systems approach and found that heterogeneity in the interspecific interactions or the presence of strongly interacting species is sufficient to explain community types, independent of the topology of the underlying ecological network. By controlling the presence or absence of these strongly interacting species we can steer the microbial ecosystem to any desired community type. This open-loop control strategy still holds even when the community types are not distinct but appear as dense regions within a continuous gradient. This finding can be used to develop viable therapeutic strategies for shifting the microbial composition to a healthy configuration.

## Author Summary

We coexist with a vast number of microbes that live in and on our bodies, and play important roles in physiology and disease. Two interesting phenomena have been observed in the human microbiome. The first is the stratification of healthy individuals based on the relative abundances of their microbes, which holds promise for drastically improving personalized medicine. The second is the astounding success of fecal microbial transplantation in treating certain diseases related to disordered microbiomes. Surprisingly, both phenomena have not been analytically or quantitatively understood, despite a few early qualitative attempts. This work shows that through a dynamic systems and control theoretical approach the success of fecal microbial transplantation can be explained and that the microbiome-based stratification can be as simple as the existence of strongly interacting species.

## Introduction

Rather than simple passengers in and on our bodies, commensal microorganisms have been shown to play key roles in our physiology and in the evolution of several chronic diseases [1, 2]. Many scientific advances have been made through the work of large-scale, consortium-driven metagenomic projects, such as the *Metagenomics of the Human Intestinal Tract* (MetaHIT) [3] and the *Human Microbiome Project* (HMP) [4, 5]. In particular, the HMP has analyzed the largest cohort and set of distinct, clinically relevant body habitats to date, in order to characterize the ecology of human-associated microbial communities [4]. These results thus delineate the range of structural and functional configurations normal in the microbial communities of a healthy population, enabling future characterization of the translational applications of the human microbiome.

A recent study proposed that a healthy gut microbiome falls within one of three distinct community types, which the authors coined as “enterotypes” [6]. More specifically, the authors calculated the relative abundance profiles of microbiota at the genus level and then performed standard cluster analysis, finding three distinct clusters (enterotypes). Each enterotype is a dominated by a particular genus (*Bacteroides*, *Prevotella*, or *Ruminococcus*) but not affected by gender, age, body mass index, or nationality of the host. These results suggest that enterotyping could be an efficient way to stratify healthy human individuals. The development of personalized microbiome-based therapies would then simplify to shifting an unhealthy microbiome to one of the distinct healthy configurations.

A meta-analysis, however, suggested that enterotypes, or in general community types, could be an artifact of the small sample size in [6] and what one should expect is a continuous gradient with dense regions rather than distinct clusters [7]. The level of discreteness or continuity of the community types remains unclear. Interestingly, samples in the dense regions of this gradient are either highly abundant or deficient in *Bacteroides*[7], indicating that community types could still emerge as the dense regions within a continuous gradient. Indeed, some recent work actually supports the notion of distinct community types [8–12].

We still lack consensus on the nature and origins of community types [13–17]. In principle the presence of community types could be explained by several different mechanisms. For instance, there may be true multi-stability, i.e. multiple stable states with the same set of microbial species present in the same environment [18]. Although this type of multi-stability has been well discussed in macro-ecological systems [19], its detection in host-associated microbial communities is rather difficult and has not been demonstrated experimentally [15]. Host heterogeneity is another possible mechanism, leading to host-specific microbial dynamics (parameterized by host-specific intra- and inter-species interactions). If those interactions, which serve as parameters of the host-associated microbial ecosystems, can be classified into distinct groups, then we can numerically demonstrate that distinct community types will naturally emerge (S1 Text Sec. 6.2 and 7.1). However, the presence of classifiable microbial dynamics has not been experimentally detected. Moreover, the overwhelming success of *Fecal Microbiota Transplantation* (FMT) in treating *recurrent Clostridium Difficile Infection* (rCDI) suggests that host heterogeneity is likely playing a minor role in terms of its effect on intra- and inter-species interactions [20–22].

Here we proposed a simple mechanism, without assuming multi-stability or host heterogeneity, to explain the origins of community types. In particular, using a dynamic systems approach, we studied compositional shift as a function of species collection and demonstrated that with heterogeneous interspecific interactions, a phenomenon often observed in macroecology [23–25], community types can naturally emerge. Interestingly, this result is independent of the topology of the underlying ecological network. To our knowledge, this is the first quantitative attempt to explore the analytical basis of community types. Furthermore, community types, even when they weakly exist, can be manipulated efficiently by controlling the *Strongly Interacting Species* (SISs) only. This provides theoretical justification for translational applications of the human microbiome. Note that in this paper we use the term species in the general context of ecology, i.e. a set of organisms adapted to a particular set of resources in the environment, rather than the lowest taxonomic rank. One could think of organizing microbes by genus or operational taxonomical units as well.

### Dynamic Model

The human microbiome is a complex and dynamic ecosystem [26]. When modeling a dynamic system we should first decide how complex the model needs to be so as to capture the phenomenon of interest. A detailed model of the intestinal microbiome would include mechanistic interactions among cells, spatial structure of the human intestinal tract, as well as host-microbiome interactions [27–30]. That level of detail however is not necessary for this study, because we are primarily interested in exploring the impact that any given species has on the abundance of other species. To achieve that, a population dynamics model such as the canonical *Generalized Lotka-Volterra* (GLV) model is sufficient [15, 31]. Indeed, GLV dynamics leveraging current metagenome data has already been used for predictive modeling of the intestinal microbiota [32–34]. Consider a collection of *n* species in a habitat with the population of species *i* at time *t* denoted as *x*_*i*_(*t*). The GLV model assumes that the species populations follow a set of ordinary differential equations

$$\begin{array}{c} {\dot{x}}_{i}\left(t\right)=r_{i}x_{i}\left(t\right)+x_{i}\left(t\right)\sum_{j=1}^{n}a_{ij}\;x_{j}\left(t\right),\;i=1,\ldots ,n \end{array}$$ (1)

where $\overset{\cdot }{\left(\;\right)}=\frac{\text{d}}{\text{d}t}\left(\;\right)$. Here *r*_*i*_ is the growth rate of species *i*, *a*_*ij*_ (when *i* ≠ *j*) accounts for the impact that species *j* has on the population change of species *i*, and the terms $a_{ii}x_{i}^{2}$ are adopted from Verhulst’s logistic growth model [35]. By collecting the individual populations *x*_*i*_(*t*) into a state vector *x*(*t*) = [*x*_1_(*t*), ⋯, *x*_*n*_(*t*)]^T^, Eq (1) can be represented in the compact form

$$\begin{array}{c} \dot{x}\left(t\right)=\text{diag}\left(x\left(t\right)\right)\left(r+Ax(t)\right), \end{array}$$ (2)

where *r* = [*r*_1_, ⋯, *r*_*n*_]^T^ is a column vector of the growth rates, *A* = (*a*_*ij*_) is the interspecific interaction matrix, and diag generates a diagonal matrix from a vector. Hereafter we drop the explicit time dependence of *x*.

Next we discuss the notion of fixed point, or equivalently steady state, in the GLV dynamics. This notion is important in the context of the human microbiome, as the measurements taken of the relative abundance of intestinal microbiota in the aforementioned studies typically represent steady behavior [4, 6]. In other words, the intestinal microbiota is a relatively resilient ecosystem [36, 37], and until the next large perturbation (e.g. antibiotic administration or dramatic change in diet) is introduced, the system remains stable for months and possibly even years [38–40]. The fixed points of system Eq (2) are those solutions *x* that satisfy $\dot{x}=0$. The solution *x* = 0 (i.e. all species have zero abundance) is a trivial steady state. The set of non-trivial steady states contains those solutions x* such that *r* + Ax* = 0. When the matrix *A* is invertible, it follows that the non-trivial steady state x* = −*A*^−1^ *r* is unique [41].

**Fig 1:**
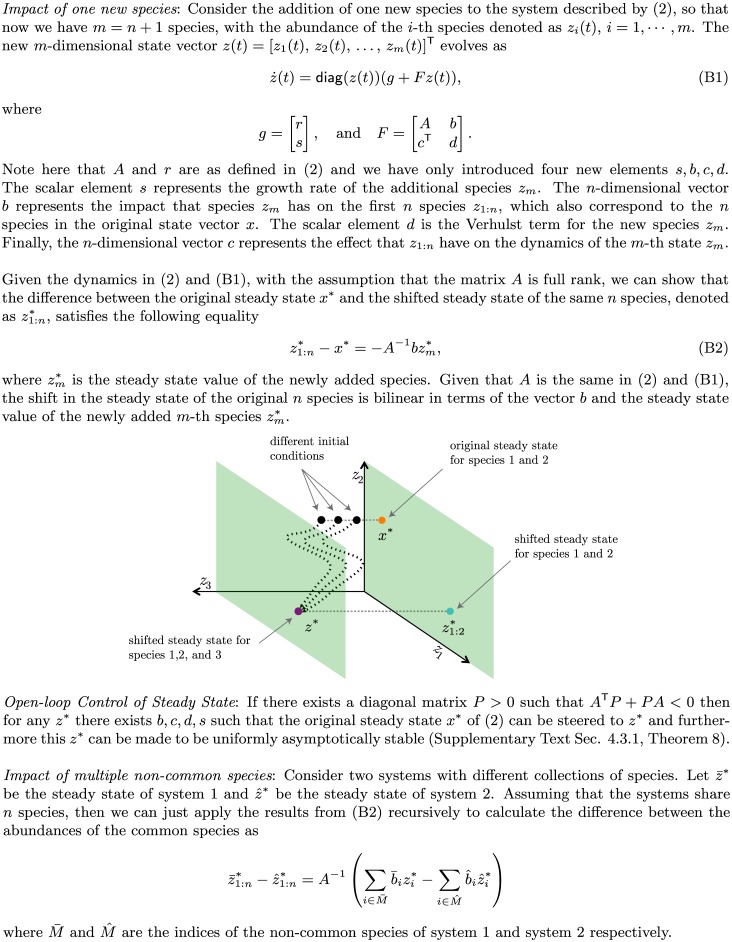
Steady state shift in the generalized Lotka-Volterra model.

Our study ultimately investigated the impact that different collections of microbial species have on their steady state abundances. In Fig 1 we presented a detailed analysis showing that if we introduce a new species into the ecosystem in Eq (2), the shift of the steady state is proportional to the interaction strengths between the newly introduced species and the previously existing ones. Similarly, if two communities with the same dynamics differ by only one species, then it is the interaction strength of that species with regard to the rest of the community that dictates how far apart the steady states of the two communities will be. This analytical result indicates that heterogeneity of interspecific interactions could lead to the clustering of steady states, and hence the emergence of community types.

To systematically investigate how changes in species collection affect the steady state shift in the GLV dynamics, we assumed that two microbial species will interact in the same fashion regardless of the host. Otherwise, if the interactions are host specific and the dynamics are classifiable, we can show that distinct community types will emerge almost trivially (S1 Text Sec. 6.2 and 7.1).

### Metacommunity and Local Communities

Consider a universal species pool, also referred to as a metacommunity [42], indexed by a set of integers **S** = {1, …, *n*}, an *n* × *n* matrix **A** representing all possible pairwise interactions between species, and a vector **r** of size *n* containing the growth rates for all the *n* species. The global parameters for the metacommunity are completely defined by the triple (**S**, **A**, **r**). We consider *q* *Local Communities* (LCs), defined by sets *S*^[*ν*]^ that are subsets of **S**, denoting the species present in LC_*ν*_ with *ν* = 1, …, *q*. This modeling procedure is inspired by the fact that alternative community assembly scenarios could give rise to the compositional variations observed in the human microbiome [42]. These LCs represent microbial communities in the same body site across different subjects. For simplicity, we assume that each LC contains only *p* species (*p* ≤ *n*), randomly selected from the metacommunity.

The GLV dynamics for each LC is given by

$$\begin{array}{c} {\text{LC}}_{\nu }:\;{\dot{x}}^{\left[\nu \right]}\left(t\right)=\text{diag}\left(x^{\left[\nu \right]}\left(t\right)\right)\left(r^{\left[\nu \right]}+A^{\left[\nu \right]}x^{\left[\nu \right]}\left(t\right)\right), \end{array}$$ (3)

where the LC specific interaction matrix and growth vector are defined as $A^{\left[\nu \right]}=A_{S^{\left[\nu \right]},S^{\left[\nu \right]}}$ and $r^{\left[\nu \right]}=r_{S^{\left[\nu \right]}}$, respectively. That is, *A*^[*ν*]^ is obtained from **A** by only taking the rows and columns of **A** that are contained in the set *S*^[*ν*]^. A similar procedure is performed in order to obtain *r*^[*ν*]^. Finally for each *x*^[*ν*]^ there is a corresponding $x^{\left[\nu \right]}\in R^{n}$ that has the abundances for species *S*^[*ν*]^ of LC_*ν*_ in the context of the metacommunity species pool **S**.

To reveal the origins of community types in the human microbiome, we decomposed the universal interaction matrix as

$$\begin{array}{c} A=NH◦Gs, \end{array}$$ (4)

which contains four components. (i) $N\in R^{n\times n}$ is the nominal interspecific interaction matrix where each element is sampled from a normal distribution with mean 0 and variance *σ*^2^, i.e. ${\left[N\right]}_{ij}∼N\left(0,\sigma^{2}\right)$. (ii) $H\in R^{n\times n}$ is a diagonal matrix that captures the overall interaction strength heterogeneity of different species. When studying the impact of interaction strength heterogeneity the diagonal elements of **H** will be drawn from a power-law distribution with exponent −*α*, i.e. ${\left[H\right]}_{ii}∼P\left(\alpha \right)$, which are subsequently normalized so that the mean of the diagonal elements is equal to 1. This is to ensure that the average interaction strength is bounded. For studies that do not involve interaction strength heterogeneity **H** is simply the identity matrix. (iii) $G\in R^{n\times n}$ is the adjacency matrix of the underlying ecological network: [**G**]_*ij*_ = 1 if species *i* is affected by the presence of species *j* and 0 otherwise. For details on the construction of **G** for different network topologies see S1 Text Sec. 3.2.2. Note that the *Hadamard product* (◦) between **H** and **G** represents element-wise matrix multiplication. (iv) The last component *s* is simply a scaling factor between 0 and 1. Finally, we set [**A**]_*ii*_ = −1. The presence of the scaling factor *s* and setting the diagonal elements of **A** to −1 are to ensure an asymptotic stability condition for the GLV dynamics (S1 Text Sec. 4.2, 4.3.3, and 4.5). The elements in the global growth rate vector **r** are taken from the uniform distribution, ${\left[r\right]}_{i}∼U\left(0,1\right)$. Details concerning the distribution N, P and U can be found in S1 Text Sec. 3.1.1.

### Origins of Community Types

**Fig 2:**
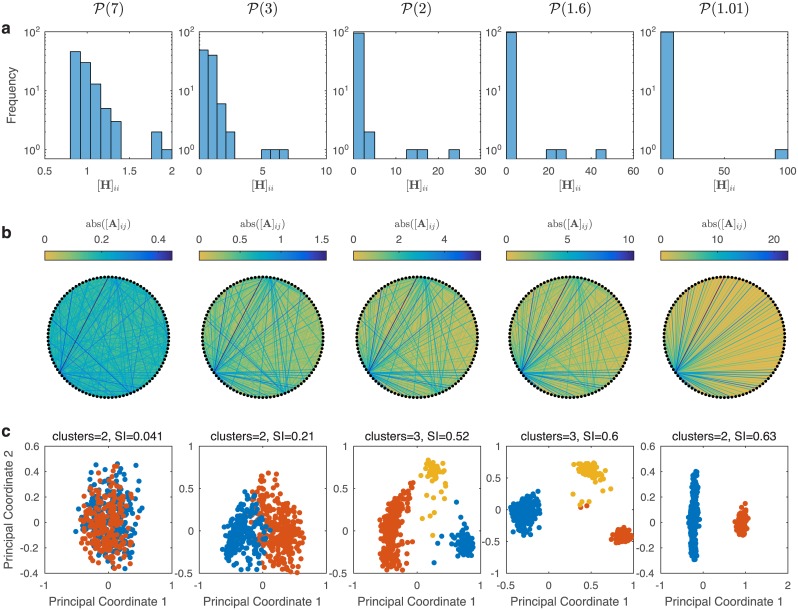
Impact of interaction strength heterogeneity on the distinctness of community types. A total of *q* = 500 local communities, each with *p* = 80 species randomly drawn from a universal pool of *n* = 100 species. The nominal components were drawn from N(0,1), the interaction heterogeneity matrix elements were taken from P(α) and *α* is varied with the set of values {7, 3, 2, 1.6, 1.01} for each column in the figure. The topology component **G** has all elements equal to 1, giving a complete graph. The scaling factor was set at *s* = 0.07. (a) Histogram of the diagonal elements of the heterogeneity matrix **H**. (b) Visualization of the universal interaction matrix **A** as a weighted adjacency matrix of a digraph. (c) Principle coordinate analyses of the normalized steady state for each local community using the Jensen-Shannon distance. The Silhouette Index and optimal number of clusters are denoted. Further details can be found in Materials and Methods.

We first studied the role of interspecific interaction strength heterogeneity on the emergence of community types. In order to achieve this, we chose the complete graph topology, i.e. each species interacts with all other species. This eliminates any *structural* heterogeneity. The nominal interaction strengths were taken from a normal distribution N(0,1), the scaling component was set to *s* = 0.7, and the interaction strength heterogeneity was varied from low heterogeneity (*α* = 7) to a high level of heterogeneity (*α* = 1.01). Fig 2 displays the distributions of the diagonal elements of the interaction heterogeneity matrix **H** at various heterogeneity levels. For each level of heterogeneity we constructed 500 LCs, each with 80 species randomly drawn from a metacommunity of 100 species. Fig 2b illustrates the global interaction matrix **A** as a weighted network. With low heterogeneity all the link weights are of the same order of magnitude. As the heterogeneity increases fewer nodes contain highly weighted links, until there is only one node with highly weighted links when *α* = 1.01. These nodes with highly weighted links correspond to SISs.

Fig 2c presents the results of *Principle Coordinates Analysis* (PCoA) of the steady states associated with the 500 different LCs as a function of *α*. For low interaction heterogeneity (*α* = 7) the classical clustering measure, Silhouette Index, is less than 0.1, suggesting a lack of clustering in the data. As the heterogeneity increases the steady states can be seen to separate in the first two principle coordinate axes. At one point (*α* = 2.0) three clusters is the optimal number of clusters. Then as *α* continues to decrease the optimal number of clusters becomes two. The fact that there are three clusters when *α* = 2.0 is not special, as a different number of optimal clusters can be observed with different model parameters or different clustering measures (see S1 Text Sec. 7.2) [7]. While the precise number of clusters is not important here, what is important is the fact that the degree of interaction strength heterogeneity controls the degree to which the clusters appear to be distinct. For low levels of interaction strength heterogeneity the clusters appear to be more like dense regions within a continuous gradient. As the heterogeneity increases, the clusters become more distinct. Indeed, having two clusters for *α* = 1.01 is to be expected, because one of the clusters is associated with all the LCs that contain the single SIS, and the other LCs that do not contain the single SIS constitute the other cluster.

The overall trend observed in Fig 2c is unaffected if the complete graph is replaced by an *Erdős-Rényi* (ER) random graph, or if the total number of LCs is increased (S1 and S2 Figs). The result is also generally unaffected by the specifics of the nominal distribution (S1 Text Sec. 7.2.1), the mean degree of the ER graph (S1 Text Sec. 7.2.2), or the number of species in the LCs (S1 Text Sec. 7.2.3). Of course, each LC can be invaded by other species that are currently absent. If this migration occurs relatively fast, then all LCs will converge to roughly the same species collection and the clustering will disappear. Hence in our modeling approach we have to assume that the migration occurs at a relatively slow time scale, and the time interval between species invasions is too long to disrupt the clustering. We also note that if heterogeneous interactions are placed at random in the network the clustering of steady states does not arise (S3 Fig). Our results are also robust (in the control theoretical sense) to stochasticity and the migration of existing species [43]. Robustness to migration is illustrated in S4 and S5 Figs, and robustness to stochastic disturbances is illustrated in S6–S8 Figs (see S1 Text Sec. 4.4 for analytical robustness results).

**Fig 3:**
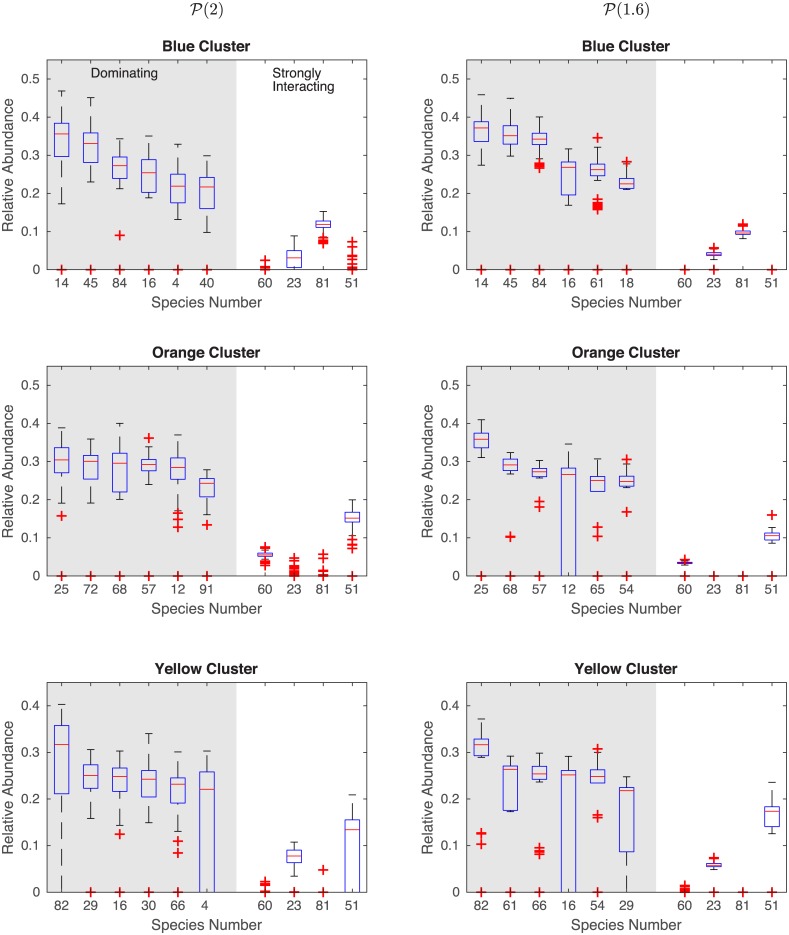
Comparison of dominating species to SISs in different community types (clusters). The relative abundances of the six most abundant species from each of the three clusters in Fig 2c for *α* = 2 and *α* = 1.6 are compared to that of the four species with the largest interaction strengths (60, 23, 81, and 51).

We can explain the above results as follows: for low interaction strength heterogeneity all of the matrices *A*^[*ν*]^ are very similar. In other words, despite containing different sets of species, all the LCs have very similar dynamics. Thus, clustering of steady states is not to be expected. As the heterogeneity of interaction strength increases, however, some of the LCs will have species that are associated with the highly weighted columns in **A**, i.e. the SISs. Fig 3 presents a detailed analysis of the most abundant (dominating) species in each of the three clusters (community types) in Fig 2c for *α* = 2 and *α* = 1.6, along with the abundances of the SISs within each cluster. It is clear that for different clusters their dominating species are different, consistent with the empirical finding that each enterotype is dominated by a different genus [6]. The SISs that are present in each cluster also vary. For instance with *α* = 1.6 all LCs in the blue cluster contain SISs number 23 and 81, and none have species 60 or 51. For the orange cluster it is the opposite scenario. All of the LCs in the orange cluster contain SISs 60 and 51, and do not contain species 23 or 81. Most of the LCs in the yellow cluster contain SISs 23 and 51. Hence, each community type is well characterized by a unique combination of SISs. Note that none of the SISs are dominating species. These findings, along with the analysis in Fig 1, suggest that heterogeneity in interaction strengths or the presence of SISs leads to the clustering of steady states, i.e. the emergence of community types.

**Fig 4:**
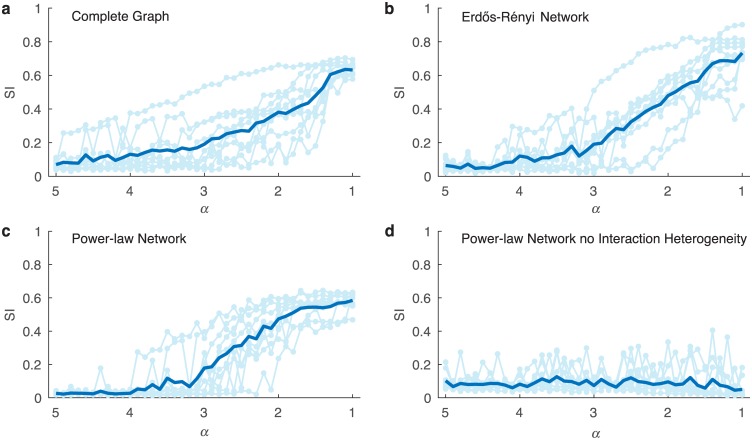
Impact of network structure on the distinctness of community types. For each type of network structure 10 different Universal Triples (**S**, **A**, **r**) with *n* = 100 species and *q* = 500 local communities of size *p* = 80 were generated with results shown in the lighter color and averaged results shown in bold. (a) *Complete graph*. Same study as in Fig 2 with *α* ∈ [5, 1). (b) *Erdős-Rényi* network (digraph) ${\left[N\right]}_{ij}∼N\left(0,1\right)$, ${\left[H\right]}_{ii}∼P\left(\alpha \right)$ where *α* ∈ (1, 5], Probability [**G**]_*ij*_ = 1 is 0.1, i.e. a mean in(out)-degree of 10, and scaling factor $s=1/\sqrt{10}$. (c) *Power-law out-degree network* ${\left[N\right]}_{ij}∼N\left(0,1\right)$, ${\left[H\right]}_{ii}∼P\left(\alpha \right)$, **G** is the adjacency matrix for a digraph with out-degree having a power-law distribution P(α). The high-degree nodes have the largest interaction scaling. (d) *Power-law out-degree network, no interactions strength heterogeneity* ${\left[N\right]}_{ij}∼N\left(0,1\right)$, **H** is the identity matrix, **G** is the adjacency matrix for a digraph with out-degree having a distribution P(α). Further details can be found in Materials and Methods.

We then studied the impact of structural heterogeneity on community types. Four different scenarios are illustrated in Fig 4: (a) a complete graph topology as in Fig 2; (b) an ER random graph as in S1 Fig; (c) a power-law out-degree network; (d) a power-law out-degree network with *no* interaction strength heterogeneity. Fig 4a, 4b and 4c support the main result shown in Fig 2, i.e. increasing interaction strength heterogeneity leads to the emergence of distinct community types. Fig 4d displays rather unexpected results as it suggests that structural heterogeneity alone does not lead to distinct community types. It is only with the inclusion of interaction strength heterogeneity that structurally heterogeneous microbial ecosystems can display strong clustering in their steady states as shown in Fig 4c. This result is rather surprising, because structural heterogeneity is observed in many real-world complex networks [44–46] and has been shown to affect many dynamical processes over complex networks [47–49].

Note that in the preparation of Fig 4 the steady state abundances were normalized to get relative abundances of the species and the Jensen-Shannon distance metric was used for clustering analysis [50]. The trends discussed above also hold when, instead of the Silhouette Index, the Variance Ratio Criterion is used as the clustering measure, or the Euclidean distance is used for clustering, or when absolute abundances are analyzed along with the Euclidean distance being used (S9, S10 and S11 Figs). S11 Fig correlates to the analytical results in Fig 1, where absolute abundances and the Euclidean distance are implicitly used.

### Control of Community Types

**Fig 5:**
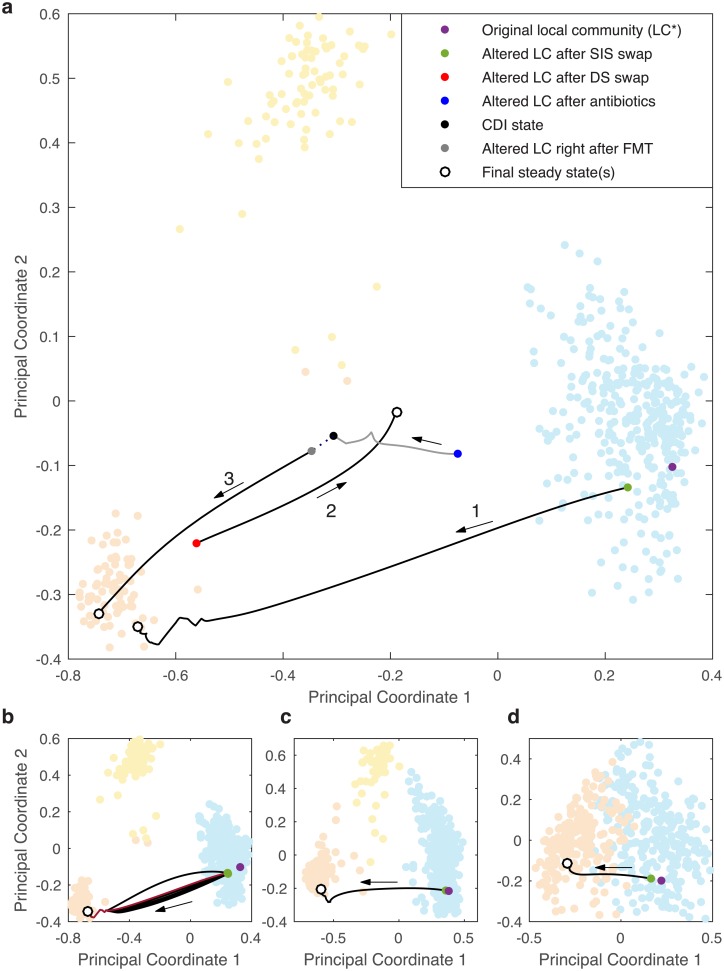
Open-loop control of the human microbiome. (a) Background of clustering analysis for *α* = 1.6 from Fig 2, but with Euclidean distance used so that a projection matrix could be found to show the trajectories in the 2D principle coordinate plane (S1 Text Sec. 5.6). We aim to steer a local community (denoted as LC*, shown in magenta) in the blue cluster to the orange cluster. Three different scenarios are presented, per the three numbers above the arrows. Scenario 1: SISs swap. The SISs (23 and 81) of LC* were replaced by the SISs present in the orange cluster (60 and 51). The initial abundances of species 60 and 51 were drawn from U(0,1), resulting in and altered community (green dot), and the GLV dynamics were simulated until the steady state was reached (white dot), which is located in the orange cluster as desired. Scenario 2: *Dominating Species* (DS) swap. The six most abundant species in LC* were removed and replaced by the six most abundant species from a local community in the orange cluster, with the initial condition after the switch of species shown as the red dot, and the dynamics were simulated until steady state was reached (white dot). Scenario 3: Fecal Microbiota Transplantation (FMT). The two SISs and 18 of the most abundant species (for a total of 20) were removed from LC* with the initial condition shown in blue (post-antibiotic state). Then the GLV dynamics were simulated (gray line) and the system converged to the black dot (CDI state). Then 1% of the steady abundances from an arbitrary LC in the orange cluster were added to the CDI state (gray dot, emulating oral capsule FMT) and the dynamics were then simulated until steady state was reached. (b) The SISs swap process was repeated ten times, each time the initial abundances of species 60 and 51 were randomly drawn from U(0,1). Nine of the simulations are shown in black and the simulation that pertains to Fig 4a is shown in maroon. (c) The same analysis as for Fig 5a, in terms of SISs swap, but for *α* = 2. (d) The same analysis as for Fig 5a, in terms of SISs swap, but for *α* = 3.

With the knowledge that each community type can be associated with a specific collection of SISs, we tested the hypothesis that a local community could be steered to a desired community type by controlling the combination of SISs only. Our results for three different scenarios are shown in Fig 5a for *α* = 1.6. The local community that was controlled in each scenario is shown in magenta and is denoted LC*, which initially belongs to the blue cluster. For Scenario 1, LC* had the SISs 23 and 81 removed, with species 60 and 51 simultaneously introduced with random initial abundances drawn from U(0,1). Recall that species 60 and 51 are the SISs present in the orange cluster. This swap of SISs shifts LC* to a slightly different state (green dot) within the blue cluster. The GLV dynamics were then simulated and the trajectory goes from the blue cluster to the orange cluster. This result was independent of the initial condition of species 60 and 51 (Fig 5b). This open-loop control of the community type by manipulating a set of SISs also works at lower levels of heterogeneity (Fig 5c and 5d). Here we use the term open-loop to contrast closed-loop control where inputs are designed with feedback so as to continuously correct the system of interest. These findings imply that the SISs, despite their low abundances, can be used to effectively control a microbial community to a desired community type.

In Scenario 2 we tested if the same result could be obtained by removing the six most abundant species from LC* and introducing the six most abundant species from the orange cluster at exactly the same abundance level as an arbitrary local community in the orange cluster. The state after this dominating species swap (red dot) starts close to the orange cluster, because the six most abundant species from a local community in that cluster were copied. The trajectory does not ultimately converge near the orange cluster, but goes toward the blue cluster instead. The trajectory, however, does not ultimately converge in the blue cluster because it does not contain any of the most abundant species present in the blue cluster.

In scenario 3 we explored how the open-loop control methodology just presented could also be used to conceptually justify the success of FMT in treating patients with rCDI [20–22]. This scenario begins by removing 20 species from LC* (the top two SISs and 18 of the most abundant spaces) so as to emulate the effect of broad-spectrum antibiotics, resulting in an altered community (blue dot). Then the GLV dynamics were simulated and the local community converged to a new steady state (black dot), representing the CDI state. To emulate an oral capsule FMT 1% of the species abundances from an arbitrary LC in the orange cluster, i.e. the donor, was added to the CDI state, resulting in a slightly altered community (gray dot). The GLV dynamics were then simulated until the final steady state was reached (white dot). As expected the post-FMT steady state is in the orange cluster, the same cluster that is associated with the donor’s LC. Note that if during the FMT the SISs in the donor’s LC were not transplanted then the patient’s post-FMT steady state does not converge in the orange cluster (S12 Fig).

The above results indicate that the presence of SISs simplifies the open-loop control design. However, the existence of community types is not a prerequisite for deploying this control methodology. The possibility for open-loop control of the human microbiome will likely be body site specific. Our work focused on the gut specifically because of the fact that this microbial community is very likely dominated by microbe-microbe and/or host-microbe interactions, rather than external disturbances. It is yet to be determined what factors drive the dynamics in other body sites.

## Discussion

In this work we studied compositional shift as a function of species collection using a dynamic systems approach, aiming to offer a possible mechanism for the origins of community types. We found that the presence of interaction strength heterogeneity or SISs is sufficient to explain the emergence of community types in the human microbiome, independent of the topology of the underlying ecological network. The presence of heterogeneity in the interspecific interaction strengths in natural communities has been well studied in macroecology [23–25, 51]. Extensive studies are still required to explore this interesting direction in the human microbiome. While preliminary analysis is promising, all existing temporal metagenomic datasets are simply not sufficiently rich to infer the interspecific interaction strengths among all of the microbes present in and on our bodies [15] even at the genus level, let alone the species level. Recent studies have tried to overcome this issue by only investigating the interactions between the most abundant species [34]. Our results, however, suggest that SISs need not be the most abundant ones and can still play an important role in shaping the steady states of microbial ecosystems. Ignoring the lack of sufficient richness, system identification analysis with regularization and cross-validation [32, 52] of the largest temporal metagenomic dataset to date [39] does not disprove the existence of SISs. To the contrary, it supports this assertion (see S13 Fig). Permutation of the time series however also results in the identification of interaction strength heterogeneity (see S14 and S15 Figs). Hence, the presence of SISs needs to be systematically studied with novel system identification methods and perhaps further validated with co-culture experiments [15]. For example, we could first use metabolic network models to predict levels of competition and complementarity among species [53], which could then be used as prior information to further improve system identification [54].

Note that our notion of SIS is fundamentally different from that of keystone species, which are typically understood as species that have a disproportionately deleterious effect (relative to their abundance) on the community upon their removal [55]. One can apply a brute-force leave-one-out strategy to evaluate the “degree of keystoneness” of any species in a given community [56]. Even without any interaction strength heterogeneity, a given community may still have a few keystone species. The SISs defined in this work are those species that have very strong impacts (either positive or negative) on the species that they directly interact with. The presence of SISs requires the presence of interaction strength heterogeneity. We emphasize that an SIS is not necessarily a keystone species. In fact, without any special structure embedded in the interaction matrix (and hence the ecological network), there is no reason why the removal of any SIS would cause a mass extinction. It does have a profound impact on the steady-state shift, which is exactly what we expected from our analytical results presented in Fig 1.

Our findings also have important implications as we move forward with developing microbiome-based therapies, whether it be through drastic diet changes, FMT, drugs, or even engineered microbes [57–63]. Indeed, our results suggest that a few strongly interacting microbes can determine the steady state landscape of the whole microbial community. Therefore, it may be possible to control the microbiome efficiently by controlling the collection of SISs present in a patient’s gut. Finer control may be possible through the engineering of microbes. This will involve a detailed mechanistic understanding of the metabolic pathways associated with the microbes of interest. As discussed in Fig 1, given a new steady state of interest, the parameters *b*, *c*, *d*, *s* could be chosen such that the new steady state is feasible and stable (S1 Text Sec. 4.3.1). Then, with the knowledge of the appropriate parameters *b*, *c*, *d*, *s* it would be possible to introduce a known microbe with those characteristics or engineer one to have the desired properties. We emphasize that the stability and control of the microbial ecosystem must be studied at the macroscopic scale using a systems and control theoretic approach. This is similar to what is carried out in aerospace applications. The design of wings and control surfaces for an aircraft incorporate sophisticated fluid dynamic models. The control algorithms for planes however are often derived from simple linearized reduced order dynamic models where linear control techniques can be easily deployed [64]. Taken together, our results indicate that the origins and control of community types in the human microbiome can be explored analytically if we combine the tools of dynamic systems and control theory, opening new avenues to translational applications of the human microbiome.

## Materials and Methods

The methods section begins with a toy example to illustrate the construction of the universal interaction matrix **A** = **NH** ◦ **G***s* in Eq (4), where

$$\begin{array}{ccc} \text{steps:}\; & \left(i\right) & N=\left[\begin{array}{cccc} 0 & 0.2 & 0.4 & -0.1\\ 0.7 & 0 & 0.3 & 0.4\\ -0.1 & 0.7 & 0 & 0.1\\ -0.3 & -0.2 & 0.4 & 0 \end{array}\right]\\ & \left(ii\right) & H=\left[\begin{array}{cccc} 10 & 0 & 0 & 0\\ 0 & 0.2 & 0 & 0\\ 0 & 0 & 0.2 & 0\\ 0 & 0 & 0 & 0.4 \end{array}\right]\\ & \left(iii\right) & G=\left[\begin{array}{cccc} 0 & 1 & 1 & 1\\ 1 & 0 & 1 & 0\\ 1 & 0 & 0 & 0\\ 0 & 0 & 1 & 0 \end{array}\right]\\ & \left(iv\right) & s=1\\ & \left(v\right) & {\left[A\right]}_{ii}=-1\\ \text{final}\;\text{result:}\; & & A=\left[\begin{array}{cccc} -1 & 0.04 & 0.08 & -0.04\\ 7 & -1 & 0.06 & 0\\ -1 & 0 & -1 & 0\\ 0 & 0 & 0.08 & -1 \end{array}\right] \end{array}$$

Given that **H** is diagonal, it scales the columns of **N**. If one thinks of **A** as the adjacency matrix of a digraph, then **H** scales all of the edges leaving a node. Thus one can consider **H** as controlling the interaction strength heterogeneity of **A**. Given the Hadamard product between **H** and **G**, the off-diagonal elements of **G** that are zero will result in the corresponding off-diagonal elements of **A** being zero as well.

In the first study (Fig 2), to explore the impact of interaction heterogeneity on steady state shift, we varied the exponent −*α* of the power-law distribution of [**H**]_*ii*_ to generate five different universal interaction matrices **A** of dimension 100 × 100. For each universal interaction matrix **A**, the nominal component **N** consists of independent and identically distributed elements sampled from a normal distribution N(0,1). The topology for this study was a complete graph and thus all the elements in **G** are equal to 1. The heterogeneity element **H** is constructed in two steps. First, five different vectors $\bar{h}\left(\alpha \right)\in R^{100}$ are constructed where each element is sampled from a power-law distribution P(α) for *α* ∈ {7, 3, 1.6, 1.2, 1.01}. Then, each of the $\bar{h}\left(\alpha \right)$ is normalized to have a mean of 1, $h=\bar{h}/\text{mean}\left(\bar{h}\right).$ Finally the heterogeneity matrix is defined as H=diag(h). For this study *s* = 0.07, ensuring uniform asymptotic stability for the case of low heterogeneity (see S1 Text Theorem 17). The final step in the construction of **A** is to set the diagonal elements to −1.

For each *α* the following simulation steps were taken. There are a total of 100 species, **S** = {1, 2, …, 100}, in the metacommunity, and each of the 500 local communities contains 80 species, randomly chosen from **S**. The MATLAB command used to perform this step is randperm. The initial condition for each of the 500 local communities, *x*^[*ν*]^(0), were sampled from U(0,1). The dynamics were then simulated for 100 seconds using the MATLAB command ode45. If any of the 500 simulations crashed due to instability or if the norm of the terminal discrete time derivative was greater than 0.01 then that local community was excluded from the rest of the study. Those simulations that finished without crashing and with small terminal discrete time derivative were deemed steady. Less than 1% of simulations were deemed unstable in the preparation of Fig 2. It is worth noting that by constructing the dynamics as described above the abundance profiles for our synthetic data do not contain the heavy-tailed abundance profile that is observed in the HMP gut data [4].

The networks presented in the second row of Fig 2 were constructed by considering **A** as the weighted adjacency matrix of the network. Note that arrows showing directionality and self loops were suppressed. The links were color coded in proportion to the absolute value of the entries in **A**.

For the last row of Fig 2 a clustering analysis was performed. For each *α* the steady state abundances of the 500 local communities were normalized so that we have 500 synthetic microbial samples. Then *k*-medoids clustering was performed for *k* ∈ {1, 2, …, 10} using the Jensen-Shannon distance metric (S1 Text Sec. 5.1). Silhouette analysis was performed to determine the optimal number of clusters and the clustering results were illustrated in the 2-dimensional principle coordinates plot. For S1 Fig the same steps as for the preparation of Fig 2 were performed, but with **G** representing the adjacency matrix of an Erdős-Rényi digraph with mean degree of 20 (mean in-degree of 10 and mean out-degree of 10) and $s=1/\sqrt{10}$. Details on the construction of an Erdős-Rényi digraph can be found in S1 Text Section 3.2.1. For S2 Fig the same steps as above were performed in Fig 2 but with *p* = 5,000 local communities.

Fig 4 is a macroscopic analysis of how network structure plays a role in the steady state shift with values of *α* ∈ (1, 5]. For each topology ten different universal matrices **A** were generated. Fig 4a shows the results of a complete graph and for each of the ten universal **A** the same steps as in the preparation of Fig 2 were carried out. Fig 4b shoes the result of an Erdős-Rényi random digraph topology and for each of the ten **A** matrices the same steps as in the preparation of S2 Fig were carried out. Fig 4c shows results for networks with a power-law out-degree distribution with a mean out-degree of 10, where the out-degree sequence uses the same $\bar{h}$ in the construction of **H**. More information on the construction of **G** for a power-law out-degree network can be found in S1 Text Sec. 3.2.2. Fig 4d shows results for networks with a power-law out-degree distribution with mean out-degree of 10 and there is no interaction strength heterogeneity, i.e. **H** is the identity matrix. For this study the Silhouette Index was constructed from normalized steady state data using the Jensen-Shannon distance. S9 Fig is the same as Fig 4, but instead of the Silhouette Index, the variance ratio criterion is used with the Jensen-Shannon distance, from normalized steady state abundance (S1 Text Sec. 5.4). In S10 Fig the Silhouette Index is determined from the Euclidean distance with normalized steady state abundance. Finally, in S11 Fig the Silhouette Index is determined by the Euclidean norm with the absolute steady state abundance.

Fig 5 contains a PCoA analysis of the results from Fig 2, but with the Euclidean distance being used instead of the Jensen-Shannon distance, making PCoA equivalent to principle component analysis. This enables us to project the open-loop control trajectories into the principle coordinates (S1 Text Sec 5.6). This procedure was also used in the preparation of S12 Fig.

S13–S15 Figs contain system identification analyses for temporal gut microbiome data of two subjects [39]. The data is publicly available from the metagenomics analysis server MG-RAST:4457768.3-4459735.3 and can also be accessed (as we did) from Qiita (http://qiita.ucsd.edu) under study ID 550. The processed data was downloaded as biom file “67_otu_table.biom” (2014-11-17 13:18:50.591389). The *Operational Taxonomic Units* (OTUs) were then grouped from the genus level and up, depending on the availability of known classifications for OTUs, and converted to a txt file using MacQIIME version 1.9.0-20140227 with the command summarize_taxa.py with the options -L 6 -a true. Data was collected over 445 days with 336 fecal samples from Subject A and 131 fecal samples from Subject B. Details on the system identification algorithm are now given. The dynamics in Eq (2) can be approximated in discrete time as [32]

$$\begin{array}{c} e_{i}\left(k\right)+\log\left(x_{i}\left(t_{k+1}\right)\right)-\log\left(x_{i}\left(t_{k}\right)\right)=r_{i}+\sum_{j=1}^{n}a_{ij}x_{j}\left(t_{k}\right) \end{array}$$ (5)

for *i* = 1, 2, …, *n* where *k* = 1, 2, …, *N* − 1 is the sample index, *N* is the total number of samples, *t*_*k*_ is the time stamp of sample *k*, and *e* is an error term that arises because of the assumption that *x*(*t*) is constant over each interval *t* ∈ [*t*_*k*_, *t*_*k*+1_). Eq (5) can be rewritten in terms of a regressor vector

$$\begin{array}{c} \phi \left(k\right)={\left[1,x_{1}\left(t_{k}\right),\;x_{2}\left(t_{k}\right),\;\ldots ,\;x_{n}\left(t_{k}\right)\right]}^{T}, \end{array}$$

the parameter vector *θ*_*i*_ = [*r*_*i*_, *a*_*i*1_, *a*_*i*2_, …, *a*_*in*_] and the log difference *y*_*i*_(*k*) = log(*x*_*i*_(*t*_*k*+1_)) − log(*x*_*i*_(*t*_*k*_)) as

$$\begin{array}{c} e_{i}\left(k\right)+y_{i}\left(k\right)=\theta_{i}\phi \left(k\right). \end{array}$$

The identification problem can then be defined as finding the parameter matrix estimate $\hat{\Theta }={\left[{\hat{\theta }}_{1}^{T},{\hat{\theta }}_{2}^{T},\cdots ,{\hat{\theta }}_{n}^{T}\right]}^{T}$ of the true parameter matrix $\Theta ={\left[\theta_{1}^{T},\theta_{2}^{T},\cdots ,\theta_{n}^{T}\right]}^{T}$. Letting

$$\begin{array}{c} y\left(k\right)={\left[y_{1}\left(k\right),\;y_{2}\left(k\right),\;\ldots ,y_{n}\left(k\right)\right]}^{T} \end{array}$$

be the log difference vector for all species and *Y* = [*y*(1), *y*(2), …, *y*(*N* − 1)] be the log difference matrix the system identification problem can be compactly presented as

$$\begin{array}{c} \underset{\hat{\Theta }}{\text{min}}{\left\|Y-\hat{\Theta }\Phi \right\|}_{F}^{2}+\lambda {\left\|\hat{\Theta }\right\|}_{F}^{2} \end{array}$$

where *Φ* = [*ϕ*(1), *ϕ*(2), …, *ϕ*(*N* − 1)] is the regressor matrix, ‖⋅‖_*F*_ denotes the Frobenius norm, *λ* ≥ 0 is the Tikhonov regularization term [65]. The minimal solution to the above problem can be given directly as

$$\begin{array}{c} \underset{\hat{\Theta }}{\text{arg min}}\left({\left\|Y-\hat{\Theta }\Phi \right\|}_{F}^{2}+\lambda {\left\|\hat{\Theta }\right\|}_{F}^{2}\right)=Y\Phi^{T}{\left(\Phi \Phi^{T}+\lambda \text{I}\right)}^{-1} \end{array}$$

where *I* is the identity matrix.

Next we discuss how missing data, zero reads, and *λ* were chosen. The difference equation in Eq (5) only uses sample data over two consecutive time samples. Therefore, in the construction of *Y* and *Φ* we only include samples that for which there is data from the next day as well. Also, given that logarithms are used, when a sample has zero reads for a given taxa, a read value of one is inserted. Then relative abundances are computed before the logarithm is taken. Finally we discuss how the regularization parameter is chosen. For S13 and S14 Figs the following cross-validation is performed. For Subjects A and B two-thirds of data was used for training and one-third for testing. More precisely, for each *λ* two-thirds of the data from Subject A and two-thirds of the data from Subject B were used to identify their corresponding dynamical constants. Then the combined error from the two test sets was used to find the optimal *λ*. The regularization value used in S15 Fig is simply the same regularization value used in S13 Fig.

## Supporting Information

S1 FigImpact of interaction strength heterogeneity on the distinctness of community types.Same as Fig 2 but with the topology component **G** chosen to be an Erdős-Rényi digraph with a link probability of 0.1 and the scaling factor was set at $\text{s}=1/\sqrt{10}$.(EPS)

S2 Fig Impact of interaction strength heterogeneity on the distinctness of community types. Same as Fig 2 but with *p* = 5,000 local communities. Note that it is rather counter-intuitive that for *α* = 1.01 the Silhouette Index suggests that there are two clusters, while PCoA suggests three clusters. We emphasize that as a typical ordination method, the PCoA just produces a spatial representation of the entities in the dataset, rather than the actual determination of cluster membership. Note that as compared to Fig 2, because there are more samples in this figure, the distinctness of the clusters when *α* = 2 has shifted to more of a continuous gradient as apposed to distinct clusters.
(EPS)

S3 Fig Impact of interaction heterogeneity disbursed randomly throughout the network. The set up is the same as that of Fig 2 but instead of **H** being a diagonal matrix, it is a full matrix, so that individual interactions are scaled randomly from a power-law distribution.
(EPS)

S4 FigImpact of low levels of migration.Same as Fig 2 but with a new term *λ*(*t*)∈[0, 1]^*n*^ added to the dynamics so that now $\dot{x}=\lambda +\text{diag}\left(x\right)\left(r+Ax\right)$. In this example $\lambda_{i}∼U\left(0,0.1\right)$. The disturbance is sampled every 0.01 seconds and held constant until the next sample is taken.(EPS)

S5 FigImpact of moderate levels of migration.Same as Fig 2 but with a new term *λ*(*t*)∈[0, 1]^*n*^ added to the dynamics so that now $\dot{x}=\lambda \left(t\right)+\text{diag}\left(x\right)\left(r+Ax\right)$. In this example $\lambda_{i}∼U\left(0,1\right)$. The disturbance is sampled every 0.01 seconds and held constant until the next sample is taken.(EPS)

S6 Fig Impact of small stochastic disturbance. Same as Fig 2 but with stochastic Itô dynamics d*x* = diag(*x*)(*r* d*t* + *Ax* d*t* + *c* d*w*) where *w* is a *n*-dimensional Brownian motion and *c* represents the stochastic disturbance strength. Dynamics were simulated with a discrete time step of 0.01 seconds and *c* = 0.1.
(EPS)

S7 Fig Impact of moderate stochastic disturbance. Same as Fig 2 but with stochastic Itô dynamics d*x* = diag(*x*)(*r* d*t* + *Ax* d*t* + *c* d*w*) where *w* is a *n*-dimensional Brownian motion and *c* represents the stochastic disturbance strength. Dynamics were simulated with a discrete time step of 0.01 seconds and *c* = 0.5.
(EPS)

S8 Fig Impact of large stochastic disturbance. Same as Fig 2 but with stochastic Itô dynamics d*x* = diag(*x*)(*r* d*t* + *Ax* d*t* + *c* d*w*) where *w* is a *n*-dimensional Brownian motion and *c* represents the stochastic disturbance strength. Dynamics were simulated with a discrete time step of 0.01 seconds and *c* = 1.
(EPS)

S9 Fig Impact of network structure on the distinctness of community types. The same as Fig 4 with the *Variance Ratio Criterion* (VRC) used as apposed to the Silhouette Index for the clustering measure. See S1 Text Sec. 5.4 for details on the VRC.
(EPS)

S10 Fig Impact of network structure on the distinctness of community types. The same as Fig 4 with the Euclidean distance metric used instead of the Jensen-Shannon distance metric.
(EPS)

S11 Fig Impact of network structure on the distinctness of community types. The same as Fig 4 with the Euclidean distance metric used instead of the Jensen-Shannon distance metric and absolute abundance used instead of relative abundance.
(EPS)

S12 Fig Unsuccessful fecal microbiota transplantation. Similar to Scenario 3 shown in Fig 5a, but during the FMT, the SISs (60 and 51) of the donor’s local community in the orange cluster were not transplanted to the CDI state (black dot). This FMT resulted in a slightly altered community (gray dot) and the system eventually evolved to a steady state (white dot) thats is not in the orange cluster. Hence the FMT failed.
(EPS)

S13 Fig System identification, Tikhonov regularization *λ* = 0.0423. System identification was performed on the stool samples from the longitudinal data in [39] for two subjects as described in S1 Text where *λ* was determined by cross-validation. (a) Visualization of microbial taxa in terms of relative abundances versus day sample was taken. (b) Heat map of the interaction matrix for top 100 SISs. (c) Histogram of *Standard Deviation* (SD) of the columns of the interaction matrix. (d) List of top ten SISs in descending interaction strength (defined by the SD of each column in the interaction matrix) with relative abundances over all samples shown as a box plot. The banded structure shown in the heat map supports the assertion that SISs do exist in the gut microbiome. However this banded structure is also seen when the dates of the sample collections are permuted, see S14 and S15 Figs.
(EPS)

S14 Fig System identification, day swap, Tikhonov regularization *λ* = 0.0057. System identification was performed on the stool samples from the longitudinal data in [39], but with the collection dates permuted, *λ* was determined by cross-validation on the permuted data. (a) Visualization of microbial taxa in terms of relative abundances versus day sample was taken (not permuted samples). (b) Heat map of the interaction matrix for top 100 SISs. (c) Histogram of *Standard Deviation* (SD) of the columns of the interaction matrix. (d) List of top ten SISs in descending interaction strength (defined by the SD of each column in the interaction matrix) with relative abundances over all samples shown as a box plot. Even though the sample days have been permuted the banded structure still persists.
(EPS)

S15 Fig System identification, day swap, Tikhonov regularization *λ* = 0.0423. System identification was performed on the stool samples from the longitudinal data in [39], but with the collection dates permuted, *λ* was selected to be the same as in S13 Fig. (a) Visualization of microbial taxa in terms of relative abundances versus day sample was taken (not permuted samples). (b) Heat map of the interaction matrix for top 100 SISs. (c) Histogram of *Standard Deviation* (SD) of the columns of the interaction matrix. (d) List of top ten SISs in descending interaction strength (defined by the SD of each column in the interaction matrix) with relative abundances over all samples shown as a box plot. For the permuted data when *λ* is larger than the optimal value from the cross-validation the identification method biases towards making the most abundant species also the SISs.
(EPS)

S1 Text Detailed treatment of necessary mathematical components. S1 Text contains discussions on: random variables, random matrices, dynamic stability, clustering, ordination, modeling, and more simulation results.
(PDF)
