# Supplementary material for: On the Origins and Control of Community Types in the Human Microbiome
Source: PLoS Comput Biol. 2016 Feb 11;12(2):e1004688. doi: 10.1371/journal.pcbi.1004688 (PMC4750989; doi:10.1371/journal.pcbi.1004688)
Supplement: S1 Text — contains discussions on: random variables, random matrices, dynamic stability, clustering, ordination, modeling, and more simulation results. (PDF) [file pcbi.1004688.s016.pdf]

Supplementary Text

1 Introduction

The purpose of this supplement is to give a self-contained, rigorous account of the topics needed to fully understand the main text. The supplement is organized as follows. Section 2 contains some remarks on notation. Section 3 covers the basics of random variables, gives details on how one constructs a well defined random variable from a power-law distribution, and covers some basic results in random matrix theory, Wigner's circle and semi-circle laws. Section 4 contains details on the definition of asymptotic stability, how this relates to the generalized Lotka-Volterra dynamics, some resent results on diagonal stability are discussed, and new results regarding the stochastic stability of Lotka-Volterra dynamics is discussed. Section 5 gives the details of the multi-dimensional scaling analysis and clustering algorithms employed in this study. Section 6 gives the details of our modeling approach. Section 7 contains simulation results that are intended to complement the figures in the main text and the supplementary figures.

Much of the foundational material is well known in each of their respective areas. The diagonal stability result for random matrices has never been discussed in the context of Lotka-Volterra dynamics. The discussions regarding stable steady state shift for Lotka-Volterra dynamics are the first of their kind as well.

Those familiar with probability theory need only read §3.3 for a refresher on Wigner random matrices and the rest of §3 can be skipped. Those familiar with stability need only visit §4.3 and §4.5. That being said, even the seasoned stability theorist may find new things in the stochastic subsection of §4.4. Section 5 can be skipped for those familiar with the commonly used tools in clustering analysis.

2 Notation

Throughout we denote the real numbers as $\mathbb{R} = (-\infty, \infty)$, and the complex numbers as $\mathbb{C}$. A number $z \in \mathbb{C}$ if $z = x + iy$ where $x, y \in \mathbb{R}$ and $i \triangleq \sqrt{-1}$. The positive real numbers are denoted as $\mathbb{R}_{>0} \triangleq (0, \infty)$ and the non-negative reals as $\mathbb{R}_{\geq 0} \triangleq [0, \infty)$. The n-dimensional reals are denoted as $\mathbb{R}^n$, $\mathbb{R}^n_{>0}$ denotes the n-dimensional space of positive vectors which we will refer to as the *positive orthant*, and $\mathbb{R}^n_{\geq 0}$ as the *non-negative orthant*. An $m \times n$ matrix A of values in the reals is denoted as $A \in \mathbb{R}^{m \times n}$. The element of A in the i-th row and the j-th column will often be denoted with lower case letters as $a_{ij} = [A]_{ij}$. If there is an issue with formatting and it is not clear that both elements i and j are subscripts in the previous notation then it is

equivalent to denote a_{ij} as $a_{i,j}$. The dual notation for an $n \times n$ matrix constructed from elements a_{ij} is denoted as $A = (a_{ij})_{1 \le i,j \le n}$.

The superscript $(\cdot)^\mathsf{T}$ is used to denote transpose. The components of an n dimensional vector x are defined as follows $x = [x_1,\ x_2,\ \ldots,\ x_n]^\mathsf{T}$. We will often make use of the following nonstandard subscript notation, $x_{j:k}$ to denote the vector obtained from taking the j-th element to the k-th element of x.[1] As an example, consider $y = [1,\ 3,\ 5]^\mathsf{T}$, then $y_{2:3} = [3,\ 5]^\mathsf{T}$. The Euclidean norm of a vector $x \in \mathbb{R}^n$ is defined as $\|x\| \triangleq \left(\sum_{i=1}^n x_i^2\right)^{1/2}$. When applied to a matrix $A \in \mathbb{R}^{m\times n}$ the norm is an induced norm $\|A\| \triangleq \sup_{\|x\|=1}\|Ax\|$. For a square symmetric matrix $P = P^\mathsf{T} \in \mathbb{R}^{n\times n}$ the inequality $<\ (\le)$ is used as $P < 0\,(P \le 0)$ if and only if $x^\mathsf{T} P x < 0\,(x^\mathsf{T} P x \le 0)$ for all $x \in \mathbb{R}^n$.

The notation for union and intersection of sets is $\cup$ and $\cap$ respectively. For a set $A \subset U$ the complement of A is defined as $A^\mathsf{c} = \{y \in U\ | y \notin A\}$. The set minus notation is defined as $A \setminus B = A \cap B^\mathsf{c}$. Let A be a set, then $|A|$ denotes the cardinality of that set. If for instance $A = \{1, 2, 4\}$, then $|A| = 3$.

Given that x will primarily be defined as the state variable, we will use Y when discussing a generic random variable. The probability distribution for Y is denoted as μ_Y for which we will generically denote its probability density function as $f(y)$ where $\mu_Y = \int f(y)\,dy$. When given a probability density function $f(y)$, the notation $Y \sim f(y)$ reads as the random variable Y is drawn from the probability density function f. Also, if we generically denote the standard normal distribution as $\mathcal{N}(0, 1)$, then with a slight abuse of notation we can write $Y \sim \mathcal{N}(0, 1)$ to denote that Y is drawn from the standard normal distribution. The notation $X \equiv Y$ is used to denote when two random variables are drawn from the same distribution.

We will also make use of the following asymptotic notation. $f(n) = O(g(n))$ if there exists a C independent of n such that $\|f(n)\| \le C\|g(n)\|$ for n sufficiently large. Another form of asymptotic notation that will be borrowed is the following, $f(n) = o(g(n))$ if there exists a $c(n) \ge 0$ where $\lim_{n\to\infty} c(n) = 0$ and $\|f(n)\| \le c(n)\|g(n)\|$.

Results from dynamics and control, to probability and random matrix theory will be called upon in this work. If the notation of a particular section seems to overlap, it is assumed that the convention from the field of origin overrides. For instance, when discussing dynamics a capital letter will denote a matrix, however capital letters are used in probability theory when denoting generic random variables.

3 Random Variables and Random Matrices

3.1 Primer on Random Variables

We will use the following three distributions in constructing the random interaction matrices for the microbial communities: the uniform distribution taking values in the interval $[0, 1]$, the normal (Gaussian) distribution of mean 0 and variance σ^2 and a power-law distribution with minimum value 1 and exponent $-\alpha$. One can define a random variable just with a variable space and ignore the underlying sample space [42, §1.1.2]. We however introduce the full probabilistic machinery into the discussion. Formality in this section is two fold. First, to give the reader confidence

[1] This notation was motivated by the index notation used in Matlab.

that the random variables generated from the power-law distribution are indeed well defined random variables [5]. Second, the Wigner circle law and semi-circle law can not be defined without this machinery, and these two laws are fundamental to understanding stability results that are presented in the next section. The following is a nearly verbatim presentation of probability spaces following [41, 42].

Let Ω be a sample space. When given a measure and a σ-algebra, Ω becomes a probability space $\Omega = (\Omega, \mathcal{F}, \mathbf{P})$, where $\mathcal{F}$ is a σ-algebra of subsets of Ω and $\mathbf{P}$ is a probability measure. The probability measure along with the sample space satisfy the following equality $\mathbf{P}(\Omega) = 1$. Events E are then taken from the σ-algebra and will have a probability of occurring, i.e. $E \in \mathcal{F}$ and $E \mapsto \mathbf{P}(E)$ where $\mathbf{P}(E) \in [0, 1]$. A random variable Y takes values in a measurable space $R = (R, \mathcal{R})$ where $\mathcal{R}$ is a σ-algebra of subsets of R. We will also refer to R as the variable space. The formal definition of a random variable is a map $Y : \Omega \to R$ where Y is measurable. When one asks for the probability that event Y is in $S \in \mathcal{R}$, we are interested in the probability of event $E = Y^{-1}(S)$ occurring. From the definition of our probability space Ω, this is simply $\mathbf{P}(Y^{-1}(S))$. Note that this is equivalent to $\mathbf{P}(\{\omega \in \Omega : Y(\omega) \in S\})$ which we can unambiguously denote in shorthand as $\mathbf{P}(Y \in S)$ [42, §1.1.2]. This notation makes no reference to the original sample space and thus can be used without ambiguity when discussing the probability of an event occurring. All of our probability spaces and variable spaces will be subsets of $\mathbb{R}$ or $\mathbb{C}$. Thus we will implicitly use the Borel σ-algebra. Consequently, the specific σ-algebra of interest will no longer be denoted.

We now give rigorous definitions for the probability measure μ_Y of a random variable $Y \in R = (R, \mathcal{R})$ and the corresponding probability density function f associated with μ_Y. The distribution of Y is defined as

$$\mu_Y(S) \triangleq \mathbf{P}(Y \in S).$$

Given the above definition we define the probability density function as

$$\mu_Y(S) = \int_S f(y)\, dy.$$

The cumulative distribution function is defined as

$$F_Y(y) \triangleq \mathbf{P}(Y \leq y) = \mu_Y((-\infty, x]).$$

The expected value of a random variable $Y \sim f(y)$ (taking values from the probability density function f) is defined as

$$\begin{aligned} \mathbf{E}Y &\triangleq \int_R y\, d_Y(y) \\ &= \int_R y f(y)\, dy \qquad \text{(T1)} \\ &= \int_R \mathbf{P}(Y \geq \lambda)\, d\lambda. \end{aligned}$$

Sometimes parentheses are used, i.e. $\mathbf{E}(Y)$, for clarity. The variance of a distribution is defined as

$$\mathbf{Var}(Y) \triangleq \mathbf{E}\,|Y - \mathbf{E}(Y)|^2\,. \qquad \text{(T2)}$$

Definition 1 ([Almost Surely)**.** An event E occurs *almost surely* if $\mathbf{P}(E) = 1$.

3.1.1 Distributions of interest

The uniform distribution generates random variables on the measure space $R = [0,1]$ with the following probability density function

$$u(y) = \begin{cases} 1 \text{ if } y \in [0,1] \\ 0 \text{ otherwise} \end{cases}.$$

A pseudorandom variable in $[0,1]$ can be generated uniformly using the MATLAB command `random('unif',0,1)`. We will use the shorthand notation $\mathcal{U}(0,1)$ to denote a uniform distribution taking values in $[0,1]$.

The normal distribution with mean 0 and standard deviation σ generates random variables on the measure space $\mathbb{R}$ and satisfies the well known probability density function

$$n(y) = \frac{1}{\sigma\sqrt{2\pi}} e^{-\frac{y^2}{2\sigma^2}}.$$

A pseudorandom variable with mean 0 and variance σ^2 can be generated using the MATLAB command `random('norm',0,sigma)`. We will use the shorthand notation $\mathcal{N}(0,\sigma^2)$ to denote a normal distribution with mean 0 and standard deviation σ. A log-normal distributed random variable is simply one in which $Y = \mathbf{e}^X$ where $X \sim \mathcal{N}(0,\sigma^2)$.

The generic power-law used in this work generates random variables on the measure space $[1,\infty)$ with the following probability density function

$$p(y) = (\alpha - 1)y^{-\alpha} \tag{T3}$$

where $\alpha > 1$ [5, Equation (2.2)]. We will use the following short hand notation $\mathcal{P}(\alpha)$ to denote a power-law distribution with exponent $-\alpha$.

Later we will generate power-law distributions from uniform distributions, and thus we have the following simple result. Let U be a uniform random variable from the set $[0,1)$, then we can generate a random variable P with a power-law distribution in $[1,\infty)$ using the following measurable monotonic function $r : [0,1) \to [1,\infty)$,

$$r(U) = (1 - U)^{\frac{1}{1-\alpha}}. \tag{T4}$$

Now, letting $P \triangleq r(U)$ we can see that P indeed satisfies all of the requirements to be a well defined random variable. P is a measurable function defined from a probability space (the variable space of U with a probability measure $\mathbf{P}$) to an event space by a measurable function r. This mapping for the generation of a random variable P with powerlaw distribution is illustrated with the following commutative diagram.

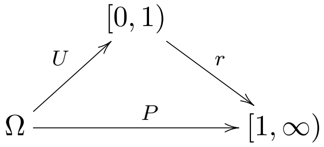

Random variables satisfying a power-law probability density function can be generated in MATLAB using the following line `(1-random('unif',0,1))^(1/(1-alpha))`.

The nice trick in (T4) is introduced in the literature without proof, and is simply denoted as following from the fundamental transformation law of probabilities [35,

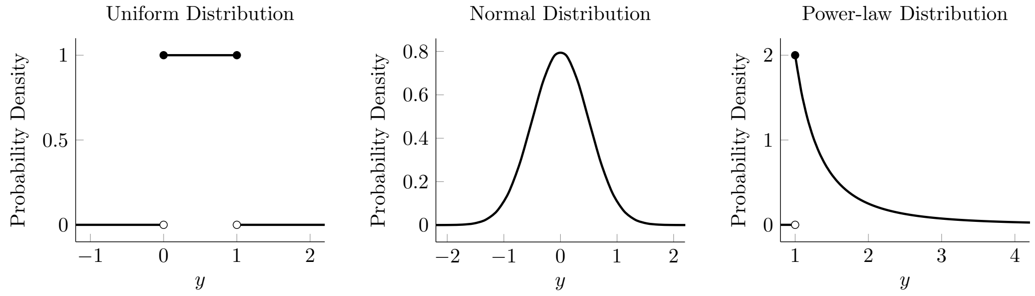

Supplementary Text Figure T1: Illustration of the distributions for the three types of probability functions used in this work, from left to right: uniform, normal, and power-law.

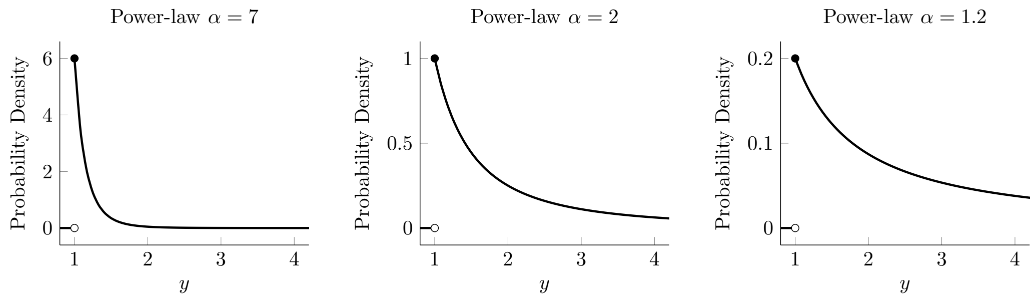

Supplementary Text Figure T2: Illustration of the power-law distribution from low heterogeneity to high heterogeneity.

§7.3]. Given the rigorous introduction of probability theory at the beginning of this section, we can however directly derive this result by analyzing the probability that $P > y$ under the assumption that there exists a monotonically increasing bijection $r : [0, 1) \to [1, \infty)$, which yields

$$\begin{aligned}\int_y^\infty p(x)dx &= \mathbf{P}(P > y) \\ &= \mathbf{P}(r(U) > y) \\ &= \mathbf{P}(U > r^{-1}(y)) \\ &= \int_{r^{-1}(y)}^\infty u(x)dx.\end{aligned} \tag{T5}$$

The transition from line 2 to line 3 in the above equality follows from the fact that r is monotonically increasing and thus when the inverse is taken the sign of the inequality is preserved. Integrating the above equality it follows that

$$y^{1-\alpha} = 1 - r^{-1}(y).$$

Substituting $z = r^{-1}(y)$ and solving for y in the above equality it follows that

$$y = (1 - z)^{\frac{1}{1-\alpha}}$$

which proves the relation in (T4).

3.2 Random Network Models

Formally a digraph $\mathcal{G}$ is defined by the double $(\mathcal{V}, \mathcal{E})$ where $\mathcal{V} = \{1, 2, \ldots, n\}$ is the vertex set and the directed edges are defined by the ordered pairs $(i, j) \in \mathcal{E} \subset \mathcal{V} \times \mathcal{V}$. An element $(i, j) \in \mathcal{E}$ if and only if there is a directed edge from vertex i to vertex j. We are primarily interested in the adjacency matrix $\mathcal{A}$ of the digraph which is defined as

$$[\mathcal{A}]_{ij} = \begin{cases} 1 & \text{if } (j, i) \in \mathcal{E} \\ 0 & \text{otherwise} \end{cases}.$$

When $[\mathcal{A}]_{ij} = 1$ for all i and j the digraph is said to be *complete*. Note that we allow for self-loops in our construction. Two other digraph topologies to be discussed shortly are the Erdős-Rényi (Gilbert) random digraph and the power-law degree digraph.

3.2.1 Erdős-Rényi (Gilbert) Digraph

An Erdős-Rényi (Gilbert) digraph is a digraph $\mathcal{G}(n, p)$ of n nodes, where the probability of a directed edge from node i to node j is p for any $i, j \in \mathcal{V}$. The adjacency matrix for this model is constructed as follows. Let $G \in [0, 1]^{n \times n}$ be a matrix with elements independently sampled from the uniform distribution between 0 and 1, $[G]_{ij} \sim \mathcal{U}(0, 1)$. Then let $\mathcal{A}$ be defined as follows

$$[\mathcal{A}]_{ij} = \begin{cases} 1 & \text{if } [G]_{ij} < p \\ 0 & \text{otherwise} \end{cases}.$$

If one is interested in defining an Erdős-Rényi model for a 100 node digraph with an expected mean in-degree (or out-degree) of 10, then simply set $p = 10/100$ in this construction.

While the above model is often credited to Erdős and Rényi [12, 13], it was actually first presented by Gilbert in [15]. We will follow convention however and simply refer to this random network model as the *Erdős-Rényi* (ER) model. The adjacency matrix for this model can be generated in MATLAB using the following boolean expression `rand(n,n)<p`.

3.2.2 Power-law Out-degree Digraph

In this section we outline how one can generate the adjacency matrix for a power-law out-degree digraph. Let $h = [h_1,\ h_2,\ \ldots,\ h_n]^\mathsf{T}$ be the column vector of out-degrees for nodes $\{1,\ 2,\ \ldots,\ n\}$. If one is interested in having a digraph with a power-law out-degree of exponent $-\alpha$ with the mean out-degree approximately d, then setting

$$h_i = \min\left\{ \left\lceil d \frac{[\bar{h}]_i}{\text{mean}(\bar{h})} \right\rceil, n \right\} \tag{T6}$$

is sufficient, where $[\bar{h}]_i \sim \mathcal{P}(\alpha)$, $i = 1, 2, \ldots, n$. Note that $\lceil \cdot \rceil$ is the ceiling operator. We also note that this will not guarantee that the mean out-degree is d for any finite sized digraph. Finally the adjacency matrix is constructed by selecting h_i random elements in column i of $\mathcal{A}$ and setting them to 1.

This method of generating power-law degree distributions leaves much to be desired. It is not based on any known theory for power-law degree graphs [3, 28]. So as to be able to compare Erdős-Rényi digraphs with the above power-law digraphs we have normalized by degree, which is not a common practice in the literature either. We note also that few authors have rigorously analyzed power-law digraphs with the exception of [3, §11] and [2].

3.3 Spectrum of Random Matrices

Two classic results from Wigner [44–46] are now discussed. First we define the *Empirical Spectral Distribution* (ESD) of an $n \times n$ real valued matrix A as $\mu_A : \mathbb{C} \to \mathbb{N}$

$$\mu_A(z) \triangleq \frac{1}{n} \left|\{1 \le i \le n : \operatorname{Re}\lambda_i(A) \le \operatorname{Re} z,\ \operatorname{Im}\lambda_i(A) \le \operatorname{Im} z\}\right|.$$

Recall that when applied to a finite set $|\cdot|$ denotes the cardinality of that set. The ESD simply counts the number of eigenvalues of A within radius $|z|$ of the origin. A weaker version of [43, Theorem 1.10] is now stated.

Theorem 1. *Let B_n be a real $n \times n$ matrix whose elements are independently and identically distributed random variables with mean 0 and variance 1. Then it follows that $\mu_{B_n/\sqrt{n}}$ converges almost surely to the uniform disk in the complex plane, $\mathbf{1}_{|z|\le 1}$, with probability 1. Let R denote the variable space for the i.i.d. random variables, then*

$$\mathbf{P}\left(\limsup_{n\to\infty}\left|\int_{\mathbb{C}} h(z)\, d\mu_{B_n/\sqrt{n}}(z) - \int_{\mathbb{C}} h(z)\mathbf{1}_{|z|\le 1}\, dz\right| \le \epsilon\right) = 1$$

for all $\epsilon > 0$ and every bounded continuous function $h : R \to \mathbb{C}$.

The semi-circle distribution is defined as $\mu_{\mathrm{sc}}(S) \triangleq \int_S \rho_{\mathrm{sc}}(y)dy$ where the probability density function is defined as

$$\rho_{sc}(y) = \begin{cases} \frac{1}{2\pi}(4-y^2)_+^{1/2}, & |y| \le 2 \\ 0, & |y| > 2 \end{cases}$$

We now state a more conservative version of [40, Theorem 5].

Theorem 2. *Let M_n be a symmetric $n \times n$ matrix whose diagonal and upper right elements are independently and identically distributed random variables with mean 0 and variance 1. Then it follows that $\mu_{M_n/\sqrt{n}}$ converges in the sense of probability to μ_{sc}. Let R denote the variable space for the i.i.d. random variables, then*

$$\liminf_{n\to\infty} \mathbf{P}\left(\left|\int_{-2}^{x} h(y)\, d\mu_{B_n/\sqrt{n}}(y) - \int_{-2}^{x} h(y) d\mu_{\mathrm{sc}}(y)\right| \le \epsilon\right) = 1$$

for all $\epsilon > 0$ and every bounded continuous function $h : R \to \mathbb{R}$.

A conservative version of [1, Theorem A] is now stated

Theorem 3. *Let M_n be a symmetric $n \times n$ matrix whose upper right elements are independently and identically distributed random variables with mean 0 and variance σ^2, and whose diagonal elements are independently and identically distributed with*

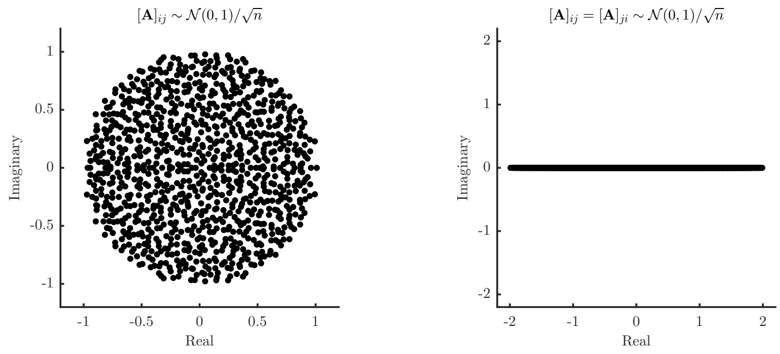

Supplementary Text Figure T3: Eigenvalues for (left) random matrix and (right) symmetric random matrix of dimension $n \times n$ with $n = 1000$ where elements are drawn from the distribution $\mathcal{N}(0,1)/\sqrt{n}$.

mean 0 and finite variance. Then it follows that $\sup_{1\le i\le n} |\lambda_i(M_n)| = 2\sigma\sqrt{n}(1+o(1))$ *asymptotically almost surely. Stated another way,*

$$\liminf_{n\to\infty} \mathbf{P}\left(\sup_{1\le i\le n} |\lambda_i(M_n)| = 2\sigma\sqrt{n}(1+o(1))\right) = 1.$$

Theorem 1 states that the spectral distribution of a random matrix with elements drawn from a normal distribution with mean 0 and variance $1/n$ converges to the unit disk centered at the origin of the complex plain. For the same random matrix but with the added assumption of symmetry, the spectrum converges to the line segment $[-2, 2]$ on the real line, see Figure T3. The spreading of the spectrum from a diameter of 2 to a diameter of 4 can be explained by the fact that all 2-cycles in the matrix now have a positive loop game. Positive feedback for 2-cycles always repels the eigenvalues along the real axis [14].

4 Stability

4.1 Primer on Stability

Consider the time-varying dynamical system defined by

$$\begin{aligned} \dot{x}(t) &= f(x(t), t) \\ x(t_0) &= x_0 \end{aligned} \tag{T7}$$

where $x : \mathbb{R} \to \mathbb{R}^n$ is the state vector, t is time, t_0 is the initial time, and $\dot{(\ )} \triangleq \frac{\mathrm{d}}{\mathrm{d}t}(\ )$. Let $x^* \in \mathbb{R}^n$ be the equilibrium solution so that $f(x^*, t) = 0$ for all t. The solution to the ordinary differential equation in (T7) is a transition function $\phi(t; x_0, t_0)$ such that $\phi(t_0; x_0, t_0) = x_0$ and $\dot{\phi}(t; x_0, t_0) = f(\phi(t; x_0, t_0), t)$. For existence and uniqueness conditions see [6]. Below we give the various definitions of stability as defined in [17, 23, 30, 34].

Definition 2 (Stability)**.** Let $t_0 \ge 0$, the equilibrium x^* is

(i) *Stable*, if for all $\epsilon > 0$ there exists a $\delta(\epsilon, t_0) > 0$ such that $\|x_0 - x^*\| \le \delta$ implies $\|\phi(t_0; t_0, x_0) - x^*\| \le \epsilon$ for all $t \ge t_0$.

(ii) *Attracting*, if there exists a $\rho(t_0) > 0$ such that for all $\eta > 0$ there exists a $T(\eta, x_0, t_0)$ such that $\|x_0 - x^*\| \leq \rho$ implies $\|\phi(t; x_0, t_0) - x^*\| \leq \eta$ for all $t \geq t_0 + T$.

(iii) *Uniformly Stable*, if the δ in (i) is uniform in t_0, thus taking the form $\delta(\epsilon)$.

(iv) *Uniformly Attracting*, if it is attracting where ρ does not depend on t_0 and $T(\eta, \rho)$ does not depend on x_0 or t_0.

(v) *Uniformly Asymptotically Stable* (UAS), if it is uniformly stable and uniformly attracting.

(vi) *Uniformly Bounded*, if for all $r > 0$ there exists a $B(r)$ such that $\|x_0 - x^*\| \leq r$ implies that $\|s(t; t_0, x_0) - x^*\| \leq B$ for all $t \geq t_0$.

(vii) *Uniformly Attracting in the Large*, if for all $\rho > 0$ and $\eta > 0$ there exists a $T(\eta, \rho)$ such that $\|x_0 - x^*\| \leq \rho$ implies $\|s(t; x_0, t_0) - x^*\| \leq \eta$ for all $t \geq t_0 + T$.

(viii) *Uniformly Asymptotically Stable in the Large* (UASL), if it is uniformly stable, uniformly bounded, and uniformly attracting in the large.

(ix) *UAS in the Positive Orthant*, if it is uniformly stable, uniformly bounded, and uniformly attracting in the positive orthant.

The precise definition of UAS is important in the context of dynamical systems. If one is able to show that a given system is UAS, then it follows that the dynamics are stable in the presence of bounded disturbances as well. That is, if the dynamics in (T7) are UAS then for $\|d(t)\| \leq \gamma$ where $\gamma > 0$ is sufficiently small, the dynamics

$$\dot{x}(t) = f(x(t)) + d(t)$$

are uniformly stable. If the dynamics in (T7) are UASL then $x(t)$ is bounded for all bounded $d(t)$ and γ can be arbitrarily large. A detailed discussion regarding this fact can be found in [17, Definition 56.1 and Theorem 56.4] and a practical example in the context of adaptive systems can be found in [33].

For a linear dynamical system $\dot{x}(t) = Ax(t)$ the uniform asymptotic stability (which is actually exponential) is verified if all of the eigenvalues of A have real parts less than zero. A well known theorem regarding the stability of linear systems, due to Lyapunov, is now stated.

Theorem 4 (Lyapunov)**.** *The eigenvalues of a real matrix A have all real parts less than zero if and only if there exists a $P = P^\mathsf{T} > 0$ such that $A^\mathsf{T} P + PA < 0$.*

A stronger version of Thereom 4 will be needed when discussing the stability of Lotka-Volterra dynamics.

Definition 3. If there exists a diagonal positive matrix P such that $A^\mathsf{T} P + PA < 0$ then A is said to be *Diagonally Stable*.

4.2 Stability of Generalized Lotka Volterra Dynamics

Consider dynamics of the form

$$\dot{x}_i(t) = r_i x_i(t) + x_i(t) \sum_{j=1}^{n} a_{ij} x_j(t), \quad i = 1, \ldots, n \tag{T8}$$

where $t \in [t_0, \infty)$ is time with t_0 the initial time. The state vector is denoted $x \in \mathbb{R}^n$ and defined as $x = [x_1,\, x_2,\, \ldots,\, x_n]^\mathsf{T}$. The linear terms are collected in the column vector $r = [r_1,\, x_2,\, \ldots,\, r_n]^\mathsf{T}$ and $A = (a_{ij})_{1 \geq i,j \geq n}$ captures the pair-wise interactions in the generalized Lotka-Volterra dynamics presented in (T8). The dynamics in (T8) can be compactly represented as

$$\dot{x}(t) = \mathsf{diag}(x(t))(r + Ax(t)). \tag{T9}$$

A discussion regarding the invertability of A is in order. This will become important in determining whether the system in (T8) has a unique non-trivial steady state. As discussed in the main text, the existence of the Verhulst terms $a_{ii}x_i^2$ increases the likelihood that A is full rank. First consider the case where all $a_{ij} = 0$ for all $i \neq j$, then a necessary and sufficient condition for A to be full rank is that all diagonal elements are non-zero.

If A is invertible then there exists a unique non-trivial steady state solution of the dynamics in (T8), denoted as $x^* = -A^{-1}r$ [16]. We are interested in answering the following question. Given an ecological system of n species, is it possible to introduce another species and drive the system to any non-trivial steady state of our choosing? We will show that the answer is yes, and furthermore, if the original n-dimensional ecological system satisfies a diagonal stability condition, then we can design the interaction strengths for the introduced species so that the new $n+1$ dimensional ecological system is asymptotically stable for all initial conditions in the positive orthant. We then discuss equivalent results regarding steady state shift and stability when an arbitrary number of species is added.

Consider the following set of assumptions.

Assumption 1. A is invertible.

Assumption 2. For the dynamics in (T8) the steady state solution $x^* \in \mathbb{R}^n_{>0}$.

Assumption 3. The matrix A is diagonally stable, see Definition 3.

Theorem 5 ([16, Theorem 1]). *If the system in (T8) satisfies Assumptions 1-3, then the steady state x^* is uniformly asymptotically stable for all initial conditions $x_0 \in \mathbb{R}^n_{>0}$.*

Proof. Let $V(x,t) = 2\sum_{i=1}^{n} p_i(x_i - x_i^* - x_i^* \log(x_i/x_i^*))$ be the Lyapunov candidate where p_i is the i-th diagonal element of a diagonal positive matrix P such that

$A^{\mathsf{T}}P + PA < 0$. Differentiating the Lyapunov candidate it follows that

$$\begin{aligned}\dot{V}(x,t) &= 2\sum_{i=1}^{n} p_i \left(\dot{x}_i - x_i^* \frac{\dot{x}_i}{x_i}\right) \\ &= 2\sum_{i=1}^{n} p_i (x_i - x_i^*) \frac{\dot{x}_i}{x_i} \\ &= 2\sum_{i=1}^{n} p_i (x_i - x_i^*) \left(b_i + \sum_{j=1}^{n} a_{ij} x_j\right) \\ &= 2\sum_{i=1}^{n} p_i (x_i - x_i^*) \sum_{j=1}^{n} a_{ij}(x_j - x_j^*) \\ &= (x - x^*)^{\mathsf{T}}(A^{\mathsf{T}}P + PA)(x - x^*).\end{aligned}$$

Thus the Lyapunov candidate is positive definite in $x - x^*$ and its derivative is negative definite in $x - x^*$. □

Remark 1. Note that in the original work of Goh [16], stability in the positive orthant is denoted, but in fact what he proved is *uniform asymptotic* stability in the positive orthant. It is important to distinguish the two as stability only implies boundedness of trajectories and uniform asymptotic stability implies convergence to the equilibrium as well as robustness to bounded persistent disturbances.

4.3 Stability in the presence of new species

4.3.1 Adding one new species

For the following examples we are interested in the $m = n+1$ dimensional dynamics

$$\dot{z}(t) = \mathsf{diag}(z(t))(g + Fz(t)) \tag{T10}$$

where

$$g = \begin{bmatrix} r \\ s \end{bmatrix}, \quad \text{and} \quad F = \begin{bmatrix} A & b \\ c^{\mathsf{T}} & d \end{bmatrix},$$

with A and r are as defined in (T8), and we have introduced the new elements $s, d \in \mathbb{R}$, and $b, c \in \mathbb{R}^n$. These dynamics represent the addition of one new species to the ecological system in (T8).

We now introduce an important property of diagonally stable matrices.

Theorem 6 ([38, Theorem 3.1]). *Let $A \in \mathbb{R}^{m\times m}$ be partitioned as*

$$A = \begin{bmatrix} A_{11} & A_{12} \\ A_{21} & A_{22} \end{bmatrix}$$

where $A_{11} \in \mathbb{R}^{(m-1)\times(m-1)}$, $A_{12}, A_{21}^{\mathsf{T}} \in \mathbb{R}^{m-1}$, and $A_{22} < 0$. Then A is diagonally stable if and only if A_{11} and the quantity $(A_{11} - A_{12}A_{21}/A_{22})$ have a common diagonal Lyapunov function, i.e., there exists a positive diagonal P such that $A_{11}^{\mathsf{T}}P + PA_{11} < 0$ and $(A_{11} - A_{12}A_{21}/A_{22})^{\mathsf{T}}P + P(A_{11} - A_{12}A_{21}/A_{22}) < 0$.

Lemma 1. *For any steady state solution x^* of (T8) satisfying $-Ax^* = r$ there exists b, c, d, s such that any $z^* \in \mathbb{R}^m_{>0}$ can be made to be a steady state solution of (T10).*

Proof. Any steady solution of (T10) satisfies the relation $g = -Fz^*$, which when expanded denotes the following relation

$$\begin{bmatrix} r \\ s \end{bmatrix} = -\begin{bmatrix} A & b \\ c^\mathsf{T} & d \end{bmatrix} \begin{bmatrix} z^*_{1:n} \\ z^*_m \end{bmatrix}.$$

There are $2n+2$ degrees of freedom in the variables b, c, d, s and there are only $n+1$ constraints. The variable b is fixed by the top row of the above equation and can be expressed in closed form as

$$b = -\frac{r + Az^*_{1:n}}{z^*_m}. \tag{T11}$$

Then for any c and d, s can be chosen as

$$s = -c^\mathsf{T} z^*_{1:n} - dz^*_m. \qquad \square \tag{T12}$$

Corollary 7. *For any steady state solution x^* of (T8) satisfying $-Ax^* = r$, and given any $c \in \mathbb{R}^n$ and $d \in \mathbb{R}$ there exists b, s such that any $z^* \in \mathbb{R}^m_{>0}$ can be made to be a steady state solution of (T10).*

Theorem 8. *For the dynamics in (T8) with $p = 1$ satisfying Assumption 3 there exists b, c, d, s such that any $z^* \in \mathbb{R}^m_{>0}$ can be made to be asymptotically stable for all initial conditions in the positive orthant.*

Proof. From Lemma 1 it follows that for any z^*, c and d the elements s, b are fixed. Now we will show that for any c there exists a d such that z^* is asymptotically stable. We begin by assuming that b and s are fixed by (T11) and (T12). From Assumption 3 we know that A is diagonally stable, and thus there exists an $\epsilon > 0$ and diagonal matrix $P > 0$ such that $A^\mathsf{T} P + PA \leq -\epsilon I$, where I is an appropriately dimensioned identity matrix. Choosing $d < 0$ and $|d| > 2\lambda_{\max}(P)\|bc^\mathsf{T}\|/\epsilon$, and noting that

$$(A - bc^\mathsf{T}/d)^\mathsf{T} P + P(A - bc^\mathsf{T}/d) \leq -\epsilon I + 2\lambda_{\max}(P)\|bc^\mathsf{T}\|/d$$

we can deduce that $(A - bc^\mathsf{T}/d)^\mathsf{T} P + P(A - bc^\mathsf{T}/d) < 0$. Thus $(A - bc^\mathsf{T}/d)$ and A have a common diagonal Lyapunov function. This, in addition with the fact that $d < 0$, the conditions of Theorem 6 are satisfied and thus F is diagonally stable. Now, applying Theorem 5 to the dynamics in (T10) we deduce that $z(t)$ can be made to be asymptotically stable for all $z(t_0) \in \mathbb{R}^m_{>0}$. $\square$

4.3.2 Adding an arbitrary number of species

For the following examples we are interested in the $m = n+p$ dimensional dynamics

$$\dot{z}(t) = \mathsf{diag}(z(t))(g + Fz(t)) \tag{T13}$$

where

$$g = \begin{bmatrix} r \\ s \end{bmatrix}, \quad \text{and} \quad F = \begin{bmatrix} A & B \\ C^\mathsf{T} & D \end{bmatrix},$$

with A and r are as defined in (T8), and we have introduced the new elements $s \in \mathbb{R}^p$, $D \in \mathbb{R}^{p\times p}$ and $B, C \in \mathbb{R}^{n\times p}$. These dynamics represent the addition of p new species to the ecological system in (T8).

Theorem 9. *For any steady state solution x^* of (T8) satisfying $-Ax^* = r$ there exists B, C, D, s such that any $z^* \in \mathbb{R}^m_{>0}$ can be made to be a steady state solution of (T13). Furthermore, if A is diagonally stable, then B, C, D, s can be chosen such that the system in (T13) is uniformly asymptotically stable in the positive orthant.*

Proof. Any steady solution of of (T13) satisfies the relation $g = -Fz^*$ which when expanded denotes the following relation

$$\begin{bmatrix} r \\ s \end{bmatrix} = - \begin{bmatrix} A & B \\ C^{\mathsf{T}} & D \end{bmatrix} \begin{bmatrix} z^*_{1:n} \\ z^*_{(n+1):m} \end{bmatrix}.$$

There are $2np + p^2 + p$ degrees of freedom in the variables B, C, D, s. The variable B is fixed by the top row of the above equation and must satisfy the following relation

$$Bz^*_{(n+1):m} = -(r + Az^*_{1:n}).$$

There are $n \times p$ degrees of freedom in the selection of B and only n constraints in the above equation. Thus such a B always exists for any r, A, and z^*. For any C and D, s can be chosen as

$$s = -C^{\mathsf{T}} z^*_{1:n} - Dz^*_{(n+1):m}.$$

Finally, we show that with the extra degrees of freedom in C and D there always exists a diagonal $P_1 > 0$ and $P_2 > 0$ such

$$\begin{bmatrix} A & B \\ C^{\mathsf{T}} & D \end{bmatrix}^{\mathsf{T}} \begin{bmatrix} P_1 & 0 \\ 0 & P_2 \end{bmatrix} + \begin{bmatrix} P_1 & 0 \\ 0 & P_2 \end{bmatrix} \begin{bmatrix} A & B \\ C^{\mathsf{T}} & D \end{bmatrix} < 0. \tag{T14}$$

Given that by assumption there exists a diagonal $P_1 > 0$ such $\tilde{A} \triangleq A^{\mathsf{T}} P_1 + P_1 A$, then by the Schur complement the inequality in (T14) holds if an only if

$$D^{\mathsf{T}} P_2 + 2P_2 D - (B^{\mathsf{T}} P_1 + P_2 C^{\mathsf{T}}) \tilde{A}^{-1} (CP_2 + P_1 B) < 0.$$

Given any A, B, C and positive diagonal P_1, P_2 there always exists a D such that the above inequality holds. □

4.3.3 Removing an arbitrary number of species.

Diagonally stable matrices have a very special property that every principle minor is also diagonally stable, giving us the following definition and theorem.

Definition 4. Let L be a proper subset of $N \triangleq \{1, 2, \ldots, n\}$ then a *principle minor* of $A \in \mathbb{R}^{n \times n}$ is the matrix obtained by omitting the columns and rows of A whose index appear in L.

Theorem 10 ([8, Theorem 1])**.** *If $A \in \mathbb{R}^{n \times n}$ is diagonally stable, then all principle minors of A are also diagonally stable.*

In the context of Lotka-Volterra dynamics this implies that if a given systems is diagonally stable, then even if an arbitrary numbers of species are removed from the system, the resulting system is still uniformly asymptotically stable in the positive orthant.

Corollary 11. *A necessary condition that A is diagonally stable is that all of its diagonal elements must be strictly negative.*

4.4 Robustness to disturbances.

We now consider three classes of Lotka-Volterra dynamics in the presence of disturbances, the first two are determinstic and the second is stochastic.

4.4.1 Deterministic Dynamics

In this section we will analyze the following two dynamical systems

$$\dot{x}(t) = \mathsf{diag}(x(t))(r + Ax(t) + w(t)) \tag{T15}$$

and

$$\dot{x}(t) = d(t) + \mathsf{diag}(x(t))(r + Ax(t)) \tag{T16}$$

where $d(t)$ and $w(t)$ are known to be a priori bounded. In (T15) the term $w(t)$ represents uncertainty in the growth rates of the species. While in (T15) the term w is deterministic, in the since that it is bounded, in terms of the ecology literature this term is sometimes referred to as the stochastic effect. Note that we will treat this term in a purely stochastic since shortly, complete with Itô calculus. The term $d(t)$ is a migration term. The following theorems show that in the presence of these disturbances, the systems are bounded for all initial conditions (in the positive orthant).

Theorem 12. *If the system in (T15) satisfies Assumptions 1-3 with* $\|d(t)\| \leq \alpha$ *then the state* x *is uniformly bounded for all initial conditions* $x_0 \in \mathbb{R}^n_{>0}$.

Proof. The proof is given for two different scenarios. The first scenario (when the disturbance is small) uses the same Lyapunov function as was used in the analysis of the disturbance free dynamics. In the second scenario a new Lyapunov candidate is introduced. A few definitions are needed before the different scenarios are analyzed. Let P be a diagonal positive solution to the Lyapunov equation $A^\mathsf{T} P + PA = -Q$, where $Q = Q^\mathsf{T} > 0$, and p_i denotes the i-th diagonal element of P, just as in the disturbance free case. Let $q_{\min}$ denote the minimum eigenvalue of Q and $p_{\max}$ denote the maximum diagonal element in P. Scenario: (a) the compact set

$$\{x : \|x - x^*\| \leq 2p_{\max}\alpha/q_{\min}\}$$

does not intersect any of the n-axes; and (b) when the above compact set does intersect at least one of the n-axes.

We now address the stability of Scenario (a). Let

$$V(x) = 2\sum_{i=1}^{n} p_i(x_i - x_i^* - x_i^* \log(x_i/x_i^*)) \tag{T17}$$

be a Lyapunov candidate. Differentiating the Lyapunov candidate it follows that

$$\begin{aligned}\dot{V}(x) &= 2\sum_{i=1}^{n} p_i\left(\dot{x}_i - x_i^*\frac{\dot{x}_i}{x_i}\right)\\ &= 2\sum_{i=1}^{n} p_i(x_i - x_i^*)\frac{\dot{x}_i}{x_i}\\ &= 2\sum_{i=1}^{n} p_i(x_i - x_i^*)\left(b_i + d_i(t) + \sum_{j=1}^{n} a_{ij}x_j\right)\\ &= 2\sum_{i=1}^{n} p_i(x_i - x_i^*)\left(d_i(t) + \sum_{j=1}^{n} a_{ij}(x_j - x_j^*)\right)\\ &= (x - x^*)^{\mathsf{T}}(A^T P + PA)(x - x^*) + 2(x - x^*)^{\mathsf{T}} Pd.\end{aligned}$$

Recall that $q_{\min}$ denotes the minimum eigenvalue of Q and $p_{\max}$ denotes the maximum diagonal element in P, then it follows that

$$\dot{V} \leq -q_{\min}\|x - x^*\|^2 + 2p_{\max}\|x - x^*\|\alpha.$$

Thus for all $\|x - x^*\| > 2p_{\max}\alpha/q_{\min}$, $\dot{V} < 0$ and thus $V(x(t)) < \infty$ for all $t \geq t_0$. It follows that for all $x_0 \in \mathbb{R}^n_{>0}$ the state $x(t)$ is bounded. Note that $x(t)$ can never be negative due to the fact that the disturbance appears as $x_i(t)d_i(t)$. This completes the proof for Scenario (a).

Scenario (b) has to be treated differently do to the fact that if any of the $x_i = 0$, then it follows that for the Lyapunov candidate in (T17) $V = \infty$. This was not possible in Scenario (a), but is possible in Scenario (b). We define a new Lyapunov candidate

$$V(x) = 2\sum_{i=1}^{n} v_i(x_i) \tag{T18}$$

where

$$v_i(x) = \begin{cases} p_i(x_i - x_i^* - x_i^*\log(x_i/x_i^*)) & x_i^* \leq x_i \\ 0 & 0 \leq x_i < x_i^*. \end{cases}$$

For a general set of dynamics the above candidate function could not be a Lyapunov candidate. However, do to the special form of the Lotka-Volterra dynamics $x(t) \in R^n_{\geq 0}$ for all time regardless of the specific coefficients. Any population dynamic model should have this property, as negative abundances would make no sense. Differentiating V in (T18) we have that

$$\dot{V}(x) = 2\sum_{i=1}^{n} \dot{v}_i(x_i)$$

where

$$\dot{v}_i(x) = \begin{cases} p_i(x_i - x_i^*)\left(d_i(t) + \sum_{j=1}^{n} a_{ij}(x_j - x_j^*)\right) & x_i^* \leq x_i \\ 0 & 0 \leq x_i < x_i^*, \end{cases}$$

which is continuous in x, note that $p_i(x_i - x_i^*)\left(d_i(t) + \sum_{j=1}^n a_{ij}(x_j - x_j^*)\right) = 0$ when $x_i = x_i^*$.

We now define the index set for all species as

$$\mathcal{I} = \{i : 0 \leq i \leq n, i \in \mathbb{N}\} \tag{T19}$$

and the set of indices for species abundances that are greater than x_i^* is defined as

$$\mathcal{S} = \{i : x_i > x_i^*\} \subset \mathcal{I}. \tag{T20}$$

Using this notation we can write the derivative of the Lyapunov function as

$$\dot{V}(x) = 2\sum_{i\in\mathcal{S}} p_i(x_i - x_i^*)\left(d_i + \sum_{j\in\mathcal{S}} a_{ij}(x_j - x_j^*) + \sum_{j\in\mathcal{S}^c} a_{ij}(x_j - x_j^*)\right).$$

The above equation can be rewritten in a more compact matrix-vector form as

$$\begin{aligned}\dot{V}(x) = {} & (x_{\mathcal{S}} - x_{\mathcal{S}}^*)^\mathsf{T}\left(A_{\mathcal{S},\mathcal{S}}^\mathsf{T} P_{\mathcal{S},\mathcal{S}} + P_{\mathcal{S},\mathcal{S}} A_{\mathcal{S},\mathcal{S}}\right)(x_{\mathcal{S}} - x_{\mathcal{S}}^*) + 2(x_{\mathcal{S}} - x_{\mathcal{S}}^*)^\mathsf{T} P_{\mathcal{S},\mathcal{S}} d_{\mathcal{S}} \\ & + 2\left(x_{\mathcal{S}} - x_{\mathcal{S}}^*\right)^\mathsf{T} P_{\mathcal{S},\mathcal{S}} A_{\mathcal{S},\mathcal{S}^c}\left(x_{\mathcal{S}^c} - x_{\mathcal{S}^c}^*\right).\end{aligned} \tag{T21}$$

Using Theorem 10 we can exploit a very unique property of diagonal Lyapunov functions, and that is the diagonal stability of all principle submatrices. It is also easy to confirm that the same P matrix can be used for the sub-diagonal Lyapunov equations. Therefore, it follows that $A_{\mathcal{S},\mathcal{S}}^\mathsf{T} P_{\mathcal{S},\mathcal{S}} + P_{\mathcal{S},\mathcal{S}} A_{\mathcal{S},\mathcal{S}} = -Q_{\mathcal{S},\mathcal{S}} < 0$ for any $\mathcal{S}$. Using the following definitions

$$\begin{aligned} q'_{\min}(\mathcal{S}) &= \min_i \lambda_i(Q_{\mathcal{S},\mathcal{S}}) \\ p'_{\max}(\mathcal{S}) &= \max_i \lambda_i(P_{\mathcal{S},\mathcal{S}}) \\ \beta'(\mathcal{S}) &= \|A_{\mathcal{S},\mathcal{S}^c}\|, \end{aligned} \tag{T22}$$

the equality in (T21) can be bounded as

$$\dot{V}(x) \leq -q'_{\min}(\mathcal{S})\|x_{\mathcal{S}} - x_{\mathcal{S}}^*\|^2 + 2p'_{\max}(\mathcal{S})\|x_{\mathcal{S}} - x_{\mathcal{S}}^*\|\left(\alpha + \beta'(\mathcal{S})\|x_{\mathcal{S}^c} - x_{\mathcal{S}^c}^*\|\right).$$

Given our definition of $\mathcal{S}$ it follows that $\|x_{\mathcal{S}^c} - x_{\mathcal{S}^c}^*\| \leq \|x_{\mathcal{S}^c}^*\| \leq \|x^*\|$. This allows the above inequality to be further simplified as

$$\dot{V}(x) \leq -q'_{\min}(\mathcal{S})\|x_{\mathcal{S}} - x_{\mathcal{S}}^*\|^2 + 2p'_{\max}(\mathcal{S})\|x_{\mathcal{S}} - x_{\mathcal{S}}^*\|\left(\alpha + \beta'(\mathcal{S})\|x^*\|\right). \tag{T23}$$

The bound in (T23) is still not sufficient to address stability as the bounds depend on the set of indices in $\mathcal{S}$. In a two step process we obtain uniform constants and then introduce a distance function that is independent of $\mathcal{S}$. We will now give uniform analogs to the bounds $q'_{\min}(\mathcal{S}), p'_{\max}(\mathcal{S}), \beta'(\mathcal{S})$ by taking the appropriate supremum or infimum over the powerset $2^{\mathcal{I}}$ of all subsets $\mathcal{S} \subset \mathcal{I}$. The powerset of interest is finite and thus the following are well defined

$$\begin{aligned} \bar{q} &= \inf_{\mathcal{S}\subset\mathcal{I}\setminus\emptyset} q'_{\min}(\mathcal{S}) \\ \bar{p} &= \sup_{\mathcal{S}\subset\mathcal{I}} p'_{\max}(\mathcal{S}) \\ \bar{\beta} &= \sup_{\mathcal{S}\subset\mathcal{I}} \beta'(\mathcal{S}). \end{aligned} \tag{T24}$$

Using the uniform bounds above the inequality in (T23) can be replaced by

$$\dot{V}(x) \leq -\bar{q}\|x_{\mathcal{S}} - x^*_{\mathcal{S}}\|^2 + 2\bar{p}\|x_{\mathcal{S}} - x^*_{\mathcal{S}}\| \left(\alpha + \bar{\beta}\|x^*\|\right). \tag{T25}$$

We now define a distance metric between an element $y \in \mathbb{R}^n$ and a compact set $\mathcal{A} \in \mathbb{R}^n$ as

$$d(y, \mathcal{A}) \triangleq \inf\{\|y - z\| : z \in \mathcal{A}\}. \tag{T26}$$

Given that $\mathcal{A}$ is compact, such a $z \in \mathcal{A}$ always exists. Our compact set of interest is defined as

$$\mathcal{X} = \{x : 0 \leq x_i \leq x^*_i\}. \tag{T27}$$

Using the compact set defined just above, the inequality in (T25) can be equivalently rewritten as

$$\dot{V}(x) \leq -\bar{q}d(x, \mathcal{X})^2 + 2\bar{p}d(x, \mathcal{X}) \left(\alpha + \bar{\beta}\|x^*\|\right).$$

Thus for all $d(x, \mathcal{X}) > 2\bar{p}\left(\alpha + \bar{\beta}\|x^*\|\right)/\bar{q}$, $\dot{V} < 0$. Therefore $V(x(t))$ is bounded for all $t \geq t_0$. □

Theorem 13. *If the system in (T16) satisfies Assumptions 1-3 with $\|d(t)\| \leq \alpha$ and furthermore we exclude the possibility for the disturbance to generate negative state values[2], then the state x is uniformly bounded for all initial conditions $x_0 \in \mathbb{R}^n_{>0}$.*

Proof. Consider the Lyapunov candidate in (T18) and differentiating along the system dynamics in (T16) it follows that

$$\dot{V}(x) = 2\sum_{i \in S} p_i(x_i - x^*_i)\left(d_i/x_i + \sum_{j \in \mathcal{S}} a_{ij}(x_j - x^*_j) + \sum_{j \in \mathcal{S}^c} a_{ij}(x_j - x^*_j)\right).$$

where $\mathcal{S} \subset I$ was defined in (T19) and (T20). Rearranging terms slightly with regard to d_i/x_i it follows that

$$\dot{V}(x) = 2\sum_{i \in S} p_i(x_i - x^*_i)\left(\sum_{j \in \mathcal{S}} a_{ij}(x_j - x^*_j) + \sum_{j \in \mathcal{S}^c} a_{ij}(x_j - x^*_j)\right) + 2\sum_{i \in S} d_i p_i(1 - x^*_i/x_i).$$

Given our definition of $\mathcal{S}$ it follows that $(1 - x^*_i/x_i) \leq 1$ when $i \in \mathcal{S}$. Therefore it follows that

$$\dot{V}(x) \leq 2\sum_{i \in S} p_i(x_i - x^*_i)\left(\sum_{j \in \mathcal{S}} a_{ij}(x_j - x^*_j) + \sum_{j \in \mathcal{S}^c} a_{ij}(x_j - x^*_j)\right) + 2\sum_{i \in S} |d_i|\, p_i.$$

The above inequality can be rewritten in a more compact matrix-vector form as

$$\begin{aligned}\dot{V}(x) \leq{}& (x_{\mathcal{S}} - x^*_{\mathcal{S}})^{\mathsf{T}} \left(A^{\mathsf{T}}_{\mathcal{S},\mathcal{S}} P_{\mathcal{S},\mathcal{S}} + P_{\mathcal{S},\mathcal{S}} A_{\mathcal{S},\mathcal{S}}\right)(x_{\mathcal{S}} - x^*_{\mathcal{S}}) + 2\|P_{\mathcal{S},\mathcal{S}}\|\|d_{\mathcal{S}}\| \\ &+ 2\left(x_{\mathcal{S}} - x^*_{\mathcal{S}}\right)^{\mathsf{T}} P_{\mathcal{S},\mathcal{S}} A_{\mathcal{S},\mathcal{S}^c}\left(x_{\mathcal{S}^c} - x^*_{\mathcal{S}^c}\right).\end{aligned}$$

Using the bounds in (T22) the above inequality can be reduced to

$$\dot{V}(x) \leq -q'_{\min}(\mathcal{S})\|x_{\mathcal{S}} - x^*_{\mathcal{S}}\|^2 + 2p'_{\max}(\mathcal{S})\|x_{\mathcal{S}} - x^*_{\mathcal{S}}\|\beta'(\mathcal{S})\|x_{\mathcal{S}^c} - x^*_{\mathcal{S}^c}\| + 2p'_{\max}(\mathcal{S})\alpha.$$

[2] This is not a restrictive assumption in the context of ecology as a disturbance can never result in the creation of a negative population.

Given our definition of $\mathcal{S}$ it follows that $\|x_{\mathcal{S}^c} - x^*_{\mathcal{S}^c}\| \leq \|x^*_{\mathcal{S}^c}\| \leq \|x^*\|$. This allows the above inequality to be further simplified as

$$\dot{V}(x) \leq -q'_{\min}(\mathcal{S})\|x_{\mathcal{S}} - x^*_{\mathcal{S}}\|^2 + 2p'_{\max}(\mathcal{S})\|x_{\mathcal{S}} - x^*_{\mathcal{S}}\|\beta'(\mathcal{S})\|x^*\| + 2p'_{\max}(\mathcal{S})\alpha. \quad \text{(T28)}$$

Using the definitions in (T24) and the set distance function in (T26) with the compact set $\mathcal{X}$ defined in (T27) the above inequality can be written as

$$\dot{V}(x) \leq -\bar{q}d(x,\mathcal{X})^2 + 2\bar{p}d(x,\mathcal{X})\bar{\beta}\|x^*\| + 2\bar{p}\alpha$$

Rewriting the above inequality as

$$\dot{V}(x) \leq -\frac{\bar{q}}{2}d(x,\mathcal{X})^2 - \frac{\bar{q}}{2}\left(d(x,\mathcal{X})^2 - 4\frac{\bar{p}}{\bar{q}}d(x,\mathcal{X})\bar{\beta}\|x^*\|\right) + 2\bar{p}\alpha$$

and completing the square with respect to the middle term $\frac{\bar{q}}{2}\left(d(x,\mathcal{X})^2 - 4\frac{\bar{p}}{\bar{q}}d(x,\mathcal{X})\bar{\beta}\|x^*\|\right)$ the following inequality holds

$$\dot{V}(x) \leq -\frac{\bar{q}}{2}d(x,\mathcal{X})^2 - \frac{\bar{q}}{2}\left(d(x,\mathcal{X}) - 2\frac{\bar{p}}{\bar{q}}\bar{\beta}\|x^*\|\right)^2 + 2\bar{p}\alpha + 2\frac{\bar{p}^2\bar{\beta}^2\|x^*\|^2}{\bar{q}}$$

Noting that the term $-\frac{\bar{q}}{2}\left(d(x,\mathcal{X}) - 2\frac{\bar{p}}{\bar{q}}\bar{\beta}\|x^*\|\right)^2 \leq 0$ it follows that

$$\dot{V}(x) \leq -\frac{\bar{q}}{2}d(x,\mathcal{X})^2 + 2\bar{p}\alpha + 2\frac{\bar{p}^2\bar{\beta}^2\|x^*\|^2}{\bar{q}} \quad \text{(T29)}$$

From the inequality in (T29) it follows that

$$d(x,\mathcal{X}) > 2\sqrt{\frac{\bar{p}\alpha}{\bar{q}} + \frac{\bar{p}^2\bar{\beta}^2\|x^*\|^2}{\bar{q}^2}}$$

implies $\dot{V} < 0$. Therefore, $x(t)$ is uniformly bounded and asymptotically converges to the compact set

$$d(x,\mathcal{X}) \leq 2\sqrt{\frac{\bar{p}\alpha}{\bar{q}} + \frac{\bar{p}^2\bar{\beta}^2\|x^*\|^2}{\bar{q}^2}}. \qquad \square$$

4.4.2 Stochastic Dynamics

In this section we wish to analyze a stochastic differential equation that is similar to (T15), but we no longer make the assumption that the disturbance is bounded, instead we assume that the disturbance appears as a Brownian motion $w(t) \in \mathbb{R}^n$, resulting in the following differential equation,

$$\mathrm{d}x = \mathsf{diag}(x)(r + Ax)\,\mathrm{d}t + c\,\mathsf{diag}(x)\,\mathrm{d}w. \quad \text{(T30)}$$

The variable $c \in \mathbb{R}$ will be a constant used to scale the square root of the variance of the brownian motion. It will be shown that $x(t)$ converges in a probabilistic since to a compact set which is proportional to c. The analysis of stochastic differentials is significantly more challenging than their deterministic counterparts. Before stating the stochastic version of the stability result, we first need to formally define filtration, Brownian motion, and give a key result of Itô. In this work the linear ordered set of interest will always be time $t \in [t_0, \infty)$. All of the following definitions are given in a less general context compared to their original presentation [11].

Definition 5 (Stochastic Process, [11, Part 2 §I.8])**.** Let $(\Omega, \mathcal{F}, \mathbf{P})$ be a probability space. A *stochastic process* on $(\Omega, \mathcal{F}, \mathbf{P}, [t_0, \infty))$ is a family of random variables $\{y(t), t \in [t_0, \infty)\}$ with map $(t, \omega) \mapsto y(t, \omega)$ from $[t_0, \infty) \times \Omega$ to R, the variable space of interest. Given that each instance in time $y(t)$ is a random variable, the variable space $R = (R, \mathcal{R})$ is necessarily a measurable space.

Definition 6 (Filtration, [11, Part 2 §I.1])**.** Let $(\Omega, \mathcal{F})$ be a measurable space and $t \in [t_0, \infty)$ is time, then a *filtration* of that measurable space is a map $t \mapsto \mathcal{F}(t)$ of increasing sub σ algebras such that $\mathcal{F}(s) \subset \mathcal{F}(t) \subset \mathcal{F}$ when $s \leq t$.

Definition 7 (Adaptation, [11, Part 2 §I.1])**.** Let $\{y(t), t \in [t_0, \infty)\}$ be a stochastic process from a *filtered measure space* $\{\Omega, \mathcal{F}, \mathcal{F}(\cdot)\}$ into the measurable space $\{R, \mathcal{R}\}$. The process is *adapted* to $\mathcal{F}(\cdot)$ if for each t, $y(t) : (\Omega, \mathcal{F}(t)) \to (R, \mathcal{R})$ is measurable.

Definition 8 (Progressively Measurable, [11, Part 2 §I.2])**.** Let $\{y(t), \mathcal{F}(t), t \in [t_0, \infty)\}$ be an adapted stochastic process. The function $y : [t_0, \infty) \times \Omega \to R$, where $(R, \mathcal{R})$ is a measurable space, is deemed *progressively measurable* if

$$y : ([t_0, t] \times \Omega, \mathsf{Borel}([t_0, t]) \times \mathcal{F}(t)) \to (R, \mathcal{R})$$

is measurable for any $t \geq t_0$.

Let us pause now and discuss these definitions. Defining a stochastic processes is rather obvious. We extended the definition of a random variable to incorporate time. At each fixed instance a stochastic process is nothing but a random variable. The extra definitions and the progression from filtrations, adaptations, and progressive measurability, are needed so that we can better understand what is needed to perform the following integration [39, Remark 7.1.1]. Let $\{y(\cdot), \mathcal{F}(\cdot)\}$ be an adapted stochastic process defined as before where $y : [t_0, \infty) \times \Omega \to R$. Let $f : R \to \mathbb{R}$ be bounded and $\mathcal{R}$ measurable, then the map $(t, \omega) \mapsto z = \int_{t_0}^{t} f(y(\tau, \omega))\, \mathrm{d}\tau$ need not be adapted. However if in addition to being adapted the stochastic process is progressively measurable, then z will be progressively measurable as well. This process is a key necessary ingredient when studying stochastic differential equations as it can be inherited. Next we formally define a Brownian motion and give a sufficient condition for the adaptation so that the progressive measurability condition is satisfied.

Definition 9 (Brownian, [11, Part 2 §VII.2])**.** A *Brownian* motion is an adapted stochastic process $\{w(\cdot), \mathcal{F}(\cdot)\}$ from the filtered probability space $(\Omega, \mathcal{F}, \mathbf{P}, \mathcal{F}(t), t \in [t_0, \infty))$ into the measurable space $(\mathbb{R}^n, \mathsf{Borel}(\mathbb{R}^n))$

- that is *Markovian*, i.e. when $s < t$ and $\mathcal{A} \in \mathsf{Borel}(\mathbb{R}^n)$ it follows that

  $$\mathbf{P}(w(t) \in \mathcal{A} | \mathcal{F}(s)) = \mathbf{P}(w(t) \in \mathcal{A} | w(s))$$

  almost surely.

- with a stationary stochastic transition function (the transition function is independent of the current time of the process) with density $\rho(t, \psi - \xi)$ defined on $\mathbb{R} \times \mathbb{R}^n$ relative to the n-dimensional Lebesgue measure, where ρ is defined as

  $$\rho(t, \psi; \sigma, t_0) = \begin{cases} (2\pi\sigma^2 t)^{-n/2} \exp \frac{-|\psi|^2}{2\sigma t} & \text{if } t > t_0 \\ 0 & \text{if } t \leq t_0, \end{cases}$$

  where $t \in \mathbb{R}$ is time and $\psi \in \mathbb{R}^n$ is the space variable.

- that is almost surely continuous

We are almost ready to address the problem at hand, however we still need to show how we can ensure that the Brownian motion is progressively measurable. Unfortunately we need a few more definitions and then we will show that Brownian motions can be naturally adapted so as to have the progressive measurability property.

Definition 10 (Right Continuous Filtration, [11, Part 2 §I.1])**.** Let $(\Omega, \mathcal{F}, \mathcal{F}(t), t \in [t_0, \infty))$ be a filtered measurable space, and define $\mathcal{F}^+(t) = \bigcap_{s>t} \mathcal{F}(s)$ for all $t \in [t_0, \infty)$. The filtration is *right-continuous* if $\mathcal{F}(\cdot) = \mathcal{F}^+(\cdot)$.

Theorem 14. *Let $\{y(\cdot), \mathcal{F}(\cdot)\}$ be a Brownian motion into $\mathbb{R}^n$ with respect to time $t \in [t_0, \infty)$, and if $\mathcal{F}(t)$ is generated by the null sets and $\sigma(y(s); s \leq t)$, the smallest sigma algebra for which all $y(s)$ are measureable for all $s \in [t_0, t]$, then $\mathcal{F}(\cdot) = \mathcal{F}^+(\cdot)$*

Proof. see [11, Part 2, §VI, Theorem 8] □

This theorem implies that one can assume without loss of generality that the filtration is right-continuous. That is, there is a natural way to construct them for any brownian motion.

Theorem 15. *Let $\{y(\cdot), \mathcal{F}(\cdot)\}$ be a Brownian motion with right-continuous filtration containing the null sets, then the Brownian motion is progressively measurable.*

Proof. see [31, Part A Theorem 47] □

As stated before, this progressive measurability is necessary when discussing the solutions to stochastic differential equations as it allows for integrals containing stochastic processes to inherit the progressively measurable property. We now state a classic result do to Itô [18]. Our version follows from [11, Part 2 §VIII.12].

Lemma 2. *Let $\{w(\cdot), \mathcal{F}(\cdot)\}$ be a Brownian motion into $\mathbb{R}^n$ with a right-continuous filtration containing the null sets. Consider the dynamics $x(t) \in \mathbb{R}^n$ given by*

$$\mathrm{d}x = \mu \mathrm{d}t + \sigma \mathrm{d}w$$

where $t \in [t_0, \infty)$, $\mu(x) \in \mathbb{R}^n$ is locally Lipschitz in x, and $\sigma(x) \in \mathbb{R}^{n \times n}$ is globally Lipschitz in x. Assuming $(x, t) \mapsto f(x, t) \in \mathbb{R}$ is twice differentiable and continuous in terms of x (class C^2 with respect to x) and once differentiable and continuous in terms of t (class C^1 with respect to t), then

$$\mathrm{d}f = \frac{\partial f}{\partial t}\mathrm{d}t + (\nabla_x f)^{\mathsf{T}} \mu \, \mathrm{d}t + \frac{1}{2}\mathsf{tr}(\sigma \mathsf{Hess}_x(f)\sigma) \, \mathrm{d}t + (\nabla_x f)^{\mathsf{T}} \sigma \, \mathrm{d}w.$$

Remark 2. In most constructions it is assumed that μ is globally Lipschitz. Indeed this assumption was made in one of Itô's original papers [19]. A detailed discussion regarding the existence of solutions when only locally Lipschitz conditions are assumed can be found in [36]. Exploiting the stability of our dynamics the existence and uniqueness of solutions can be deduced directly from the original work of Itô however. In our analysis we will show that for the dynamics of interest the state is

uniformly bounded almost surely, a formal definition is given just below this remark. Therefore, we can define equivalent dynamics

$$\mathrm{d}\bar{x} = \bar{\mu}\mathrm{d}t + \sigma \mathrm{d}w$$

where $\bar{\mu} = \mu$ on the compact set of interest, and zero outside this compact set. Given that μ is locally Lipschitz it follows that the function $\bar{\mu}$ with compact support is by definition globally Lipschitz. Solutions $\bar{x}$ exist, are unique, and are uniformly bounded almost surely. This then implies the existence and uniqueness of x for the dynamics $\mathrm{d}x = \mu \mathrm{d}t + \sigma \mathrm{d}w$ on the same compact set which $x(\cdot)$ remains in, almost surely.

Definition 11. The state $x(t; t_0, x_0)$ as a solution to the difference equation in (T30) is *uniformly bounded with probability one* if for ever $r > 0$ there exists a $B(r) > 0$ such that $\|x(t_0)\| \leq r$ implies

$$\mathbf{P}\left(\sup_t \|x(t)\| \leq B(r)\right) = 1$$

for all $t \geq t_0$. An equivalent statements would be $x(t)$ is *uniformly bounded almost surely.*

Theorem 16. *Consider the dynamics in (T30) for $x(t) \in \mathbb{R}^n$. Let assumptions 1-3 hold with $\{w(\cdot), \mathcal{F}(\cdot)\}$ a Brownian motion into $\mathbb{R}^n$ with a right-continuous filtration containing the null set. The following then hold*

1. *The state variable $x(\cdot)$ is uniformly bounded in expectation,*
2. *$x(\cdot)$ is uniformly bounded with probability one, and*
3. *$x(\cdot)$ asymptotically converges to the compact set*

$$\mathcal{D} \triangleq \left\{x : \|x - x^*\| \leq c\sqrt{\frac{n x^*_{\max} p_{\max}}{q_{\min}}}\right\} \tag{T31}$$

with probability one. Stated more precisely,

$$\mathbf{P}\left(\lim_{t\to\infty} x(t) \in \mathcal{D}\right) = 1. \tag{T32}$$

Sketch of the proof. This proof only outlines the analysis when the compact set $\mathcal{D}$ does not intersect any of the n-axes. The more complicated scenario when this is not the case can be handled just as it was in the proof of Theorem 12. Consider the Lyapunov candidate $V(x) = 2\sum_{i=1}^n p_i(x_i - x_i^* - x_i^* \log(x_i/x_i^*))$. Taking the differential along the lines of Lemma 2 (Itô) results in

$$\mathrm{d}V = (\nabla_x V)^\mathsf{T} \mathsf{diag}(x)(r + Ax)\,\mathrm{d}t + \frac{c^2}{2}\,\mathsf{tr}(\mathsf{diag}(x)\mathsf{Hess}_x(V)\,\mathsf{diag}(x))\,\mathrm{d}t \\ + (\nabla_x V)^\mathsf{T} c\ \mathsf{diag}(x)\,\mathrm{d}w \tag{T33}$$

Recall from the steps in the proof of Theorem 12 that

$$(\nabla_x V)^\mathsf{T}\,\mathsf{diag}(x)(r + Ax) = -(x - x^*)^\mathsf{T} Q(x - x^*)$$

where $A^{\mathsf{T}}P + PA = -Q$. Thus giving

$$\mathrm{d}V = -(x - x^*)^{\mathsf{T}}Q(x - x^*)\,\mathrm{d}t + \frac{c^2}{2}\,\mathsf{tr}(\mathsf{diag}(x)\mathsf{Hess}_x(V)\,\mathsf{diag}(x))\,\mathrm{d}t + (\nabla_x V)^{\mathsf{T}} c\;\mathsf{diag}(x)\,\mathrm{d}w \quad \text{(T34)}$$

Next, note that $[\mathsf{Hess}_x(V)]_{ii} = 2\frac{p_i x_i^*}{x_i^2}$ and $[\mathsf{Hess}_x(V)]_{ij} = 0$ when $i \neq j$. Substitution into the above equation results in

$$\mathrm{d}V = -(x - x^*)^{\mathsf{T}}Q(x - x^*)\,\mathrm{d}t + c^2\sum_i p_i\,\mathrm{d}t + (\nabla_x V)^{\mathsf{T}} c\;\mathsf{diag}(x)\,\mathrm{d}w \quad \text{(T35)}$$

Note that $x(t)$ and $\mathrm{d}w(t)$ are independent, $x(t)$ is only dependent on $\mathrm{d}w(s)$ when $s < t$. Therefore $\mathbf{E}((\nabla_x V)^{\mathsf{T}} c\;\mathsf{diag}(x)\,\mathrm{d}w) = 0$, given that for a Brownian motion $\mathbf{E}\,\mathrm{d}w = 0$.[3] Therefore

$$\frac{\mathbf{E}\,\mathrm{d}V}{\mathrm{d}t} \leq -q_{\mathrm{min}}\|x - x^*\|^2 + c^2 n x^*_{\mathrm{max}} p_{\mathrm{max}} \quad \text{(T36)}$$

where q_{min} is the minimum eigenvalue of Q, p_{max} is the maximum diagonal element in P, and x^*_{max} is the value in the vector x^*. Therefore,

$$\|x - x^*\| > c\sqrt{\frac{n x^*_{\mathrm{max}} p_{\mathrm{max}}}{q_{\mathrm{min}}}} \implies \frac{\mathbf{E}\,\mathrm{d}V}{\mathrm{d}t} < 0. \quad \text{(T37)}$$

Given that $\mathbf{E}\mathrm{d}V/\mathrm{d}t \leq 0$ outside a compact set it follows that $\mathbf{E}V$ is uniformly bounded in terms of the initial condition $x(t_0)$ [25, Lemma 5.4]. Given that $V(x) \geq 0$ is continuous in x and convex it follows from Jensen's inequality that $\mathbf{E}x$ is uniformly bounded as well [22]. Jensen's inequality will be used without reference from this point forward. Claim 1 has been proven.

We now move on to the second claim, namely that the dynamics are uniformly bounded with probability one. We address this claim with sub scenarios: (a) $x(t_0) \notin \mathcal{D}$, and (b) $x(t_0) \in \mathcal{D}$. Under scenario (a) we will now show that $\mathbf{P}(\sup V(x(t)) - V(x(t_0) \leq \delta_1) = 1$ for any $\delta_1 \in (0, \infty)$. The previous statement is equivalent to $\mathbf{P}(\sup V(x(t)) - V(x(t_0) > \delta_1) = 0$. This statement will be proved by contradiction. Assume that there exists a $\delta_2 \in (0, 1]$ such that $\mathbf{P}(\sup V(x(t)) - V(x(t_0) > \delta_1) \geq \delta_2$. Using Markov's inequality it follows that

$$\mathbf{E}\sup V(x(t)) - V(x(t_0)) \geq \delta_1\delta_2.$$

This statement however contradicts (T37) which states that outside the compact set of interest the expected value of the Lyapunov candidate is strictly decreasing. Therefore it follows that $\mathbf{P}(\sup V(x(t)) - V(x(t_0) > \delta_1) = 0$ which is equivalent to the claim that $\mathbf{P}(\sup V(x(t)) - V(x(t_0) \leq \delta_1) = 1$. Application of Jensen's inequality then implies that x is uniformly bounded with probability one for sub-scenario (a).

[3]In order to address this rigorously we would rewrite the expression in (T35) in integral form. Then we would integrate only up to a stopping time which we designed so that $(\nabla_x V)^{\mathsf{T}} c\;\mathsf{diag}(x) < \infty$ up to the time of interest. Then we would use the fact that $\mathbf{E}gw = 0$ for any bounded g. The bound used to generate the stopping time would then be relaxed and the desired result would be obtained as $t \to \infty$ using Fatou's Lemma and monotone convergence. A similar procedure is carried out in [9, (4.3)-(4.5)]

We now address sub-scenario (b) where it is assumed that $x(t_0) \in \mathcal{D}$. If $x(\cdot)$ remains in $\mathcal{D}$ for all time, then we are done and the state is uniformly bounded. Therefore, assume at some time t_1 the state $x(t_1) \notin \mathcal{D}$. This sub-scenario is now equivalent to sub-scenario (a) with time shifted. Therefore, it follows that x is uniformly bounded with probability 1 in sub-scenario (b) as well. This completes the proof of claim 2.

We approach claim 3 following a method proposed in the proof of [9, Theorem 2.1]. Consider the probability of three mutually exclusive scenarios, just as in [9, Theorem 2.1]

$$
\begin{aligned}
p_1 &= \mathbf{P}\left(\limsup_{t\to\infty} d(x(t),\mathcal{D}) = 0\right) \\
p_2 &= \mathbf{P}\left(\liminf_{t\to\infty} d(x(t),\mathcal{D}) > 0\right) \\
p_3 &= \mathbf{P}\left(\liminf_{t\to\infty} d(x(t),\mathcal{D}) = 0 \text{ and } \limsup_{t\to\infty} d(x(t),\mathcal{D}) > 0\right)
\end{aligned}
$$

We wish to prove that $p_1 = 1$ and $p_2, p_3 = 0$.

We first prove that $p_2 = 0$. This will be proved by contradiction. Assume $p_2 = \epsilon_1$ where $\epsilon_1 \in (0, 1]$, then there exists an ϵ_2 such that $\mathbf{P}\left(\liminf_{t\to\infty} d(x(t),\mathcal{D}) \geq \epsilon_2\right) = \epsilon_1$. Using Markov's inequality it follows that

$$\frac{1}{\epsilon_2}\mathbf{E}\liminf_{t\to\infty} d(x(t),\mathcal{D}) \geq \epsilon_1.$$

Multiplying both sides by ϵ_2 it follows that

$$\mathbf{E}\liminf_{t\to\infty} d(x(t),\mathcal{D}) \geq \epsilon_1\epsilon_2.$$

From the definition of lim inf it follows that for any $\epsilon_3 \in (0, \epsilon_1\epsilon_2)$ there exist a finite $T_1 \in [t_0, \infty)$ such that

$$\mathbf{E}\, d(x(t),\mathcal{D}) \geq \epsilon_3 \text{ for all } t \geq T_1. \tag{T38}$$

Therefore, for $t \geq T_1$ it follows that $\mathbf{E}\mathrm{d}V/\mathrm{d}t < 0$. Given that $V(x^*) = 0$ and $V(x)$ is strictly increasing away from $x = x^*$ and tends to infinity for large x in the positive orthant it follows that $\mathbf{E}\|x - x^*\|$ is strictly decreasing in time. Given that $\mathcal{D}$ is centered at x^*, there exists T_2 such that

$$\mathbf{E}\, d(x(t),\mathcal{D}) < \epsilon_3 \text{ for all } t \geq T_2.$$

which contradicts (T38). Therefore $p_2 = 0$. That is

$$\mathbf{P}\left(\liminf_{t\to\infty} d(x(t),\mathcal{D}) > 0\right) = 0.$$

We now establish that $p_3 = 0$. As before we achieve this by contradiction. For any $\epsilon_5 > 0$ assume

$$\mathbf{P}\left(\liminf_{t\to\infty} d(x(t),\mathcal{D}) = 0 \text{ and } \limsup_{t\to\infty} d(x(t),\mathcal{D}) \geq \epsilon_5\right) \neq 0. \tag{T39}$$

For any ϵ_6 and ϵ_7 such that $0 < \epsilon_7 < \epsilon_6 < \epsilon_5$, consider the *stopping times*[4] $T'_i : \Omega \to [t_0, \infty)$, $T''_i : \Omega \to [t_0, \infty)$ with $i \in \mathbb{N}_{>0}$, where

$$T'_i = \inf\left\{t \geq T''_{i-1} : d(x(t), \mathcal{D}) \leq \epsilon_7\right\}$$
$$T''_i = \inf\left\{t \geq T'_i : d(x(t), \mathcal{D}) \geq \epsilon_6\right\}.$$

From (T39) and the stopping times defined above, it follows that

$$\lim_{i\to\infty} T'_i = \infty \text{ and } \lim_{i\to\infty} T''_i = \infty$$

In order to continue with this proof by contradiction we need to obtain a lower bound on the expectation $T'_{i+1} - T''_i$. Indeed if this can be done, then we expect the solutions to the differential equation to spend a non negligible amount of time within a domain of the state space where the Lyapunov function will be decreasing. Thus we can see how this may lead to a contradiction, for how can trajectories be expected to spend an infinite amount of time in a domain where the Lyapunov function is always decreasing. Following the same procedures as in the proof of the bound in [9, (2.27)] it can be shown that there exists an $\epsilon_8 > 0$ such that

$$\mathbf{E}\left(T'_{i+1} - T''_i \mid \mathcal{F}(T''_i)\right) \geq \epsilon_8.$$

Using the bound above and the definition of $\mathcal{D}$ in (T31) it follows that

$$\mathbf{E}\left(\int_{T''_i}^{T'_{i+1}} q_{\min}\|x(\tau) - x^*\|^2 - c^2 n x^*_{\max} p_{\max}\, d\tau \;\middle|\; \mathcal{F}(T''_i)\right)$$
$$\geq \left(q_{\min} c\sqrt{\frac{n x^*_{\max} p_{\max}}{q_{\min}}}\epsilon_6 + q_{\min}\epsilon_6^2\right)\epsilon_8$$

From the bound in (T36) it follows that

$$\begin{aligned} V(x(t_0)) \geq& \mathbf{E}\int_{t_0}^{\infty} q_{\min}\|x(\tau) - x^*\|^2 - c^2 n x^*_{\max} p_{\max}\, d\tau \\ \geq& \sum_{i=1}^{\infty}\left(q_{\min} c\sqrt{\frac{n x^*_{\max} p_{\max}}{q_{\min}}}\epsilon_6 + q_{\min}\epsilon_6^2\right)\epsilon_8 \mathbf{P}(T''_i < \infty). \end{aligned}$$

Noting that $V(x(t_0)) < \infty$, it then follows from the Borel-Cantelli Lemma [10, Chapter III, Theorem 1.2] that $\mathbf{P}(\exists N < \infty \text{ s.t. } \forall i \geq N, \quad T''_i < \infty) = 0$ [9, (2.28)-(2.31)]. This implies that $\mathbf{P}\left(\limsup_{t\to\infty} d(x(t), \mathcal{D}) \geq \epsilon_5\right) = 0$ which contradicts (T39). Therefore $p_3 = 0$, that is

$$\mathbf{P}\left(\liminf_{t\to\infty} d(x(t), \mathcal{D}) = 0 \text{ and } \limsup_{t\to\infty} d(x(t), \mathcal{D}(0)) > 0\right) = 0.$$

Finally, given that the events associated with p_1, p_2, and p_3 are nonintersecting, it follows that $p_1 = 1$. Summarizing, we have shown that

$$p_1 = \mathbf{P}\left(\limsup_{t\to\infty} d(x(t), \mathcal{D}) = 0\right) = 1$$
$$p_2 = \mathbf{P}\left(\liminf_{t\to\infty} d(x(t), \mathcal{D}) > 0\right) = 0$$
$$p_3 = \mathbf{P}\left(\liminf_{t\to\infty} d(x(t), \mathcal{D}) = 0 \text{ and } \limsup_{t\to\infty} d(x(t), \mathcal{D}(0)) > 0\right) = 0,$$

[4] For more detail on stopping times see [11, Part 2, Chapter II].

and thus claim 2 of the theorem has been proven. We wish to reiterate the fact that this sketch barrows heavily from [9]. In most instances intermediate steps where stopping times are needed were glossed over. It might be worthwhile to revisit this analysis in the future, seeing as there does not seem to be much literature along this direction other than the references here in. ☐

Remark 3. The definition given for uniformly bounded in probability in this work is stronger than any other definitions that could be found in the literature and follows the classic definition, see Definition 2 and compare it to Definition 11. A trajectory is uniformly bounded in probability, if for all $r > 0$ there exists a $B(r) > 0$ such that $\|x(t_0)\| \leq r$ implies

$$\mathbf{P}\left(\sup_t \|x(t)\| \leq B(r)\right) = 1$$

for all $t \geq t_0$. In [9, Definition 2.2] the following definition is given, for all $r > 0$ and any $\epsilon > 0$ there exists a $B(r, \epsilon) > 0$ such that $\|x(t_0)\| \leq r$ implies

$$\mathbf{P}\left(\sup_t \|x(t)\| \leq B(r)\right) = 1 - \epsilon.$$

In [25, §1.4] the definition of uniformly bounded is defined as follows,

$$\sup_t \mathbf{P}(\|x(t)\| > R) \to 0 \text{ as } R \to \infty.$$

Our definition of asymptotic attractivity follows that of Deng and Kristić [9] as it is allready as strong as the classic definition. Again however the often cited work by Khasminskii [25] gives a much weaker definition. Compare the following definitions for asymptotically attracting

$$\mathbf{P}\left(\lim_{t\to\infty} \|x(t)\| = 0\right) = 1 \qquad \text{([9, Definition 2.2])}$$

$$\lim_{x_0\to 0} \mathbf{P}\left(\lim_{t\to\infty} \|x(t)\| = 0\right) = 1. \qquad \text{([25, Equation (5.15)])}$$

It is imperative when discussing stability that uniform bounds are achieved.

4.5 Diagonal Stability of Random Matrices

Theorem 17. *If $A_n \in \mathbb{R}^{n\times n}$ is chosen as*

$$[A_n]_{ij} \sim \frac{1}{\sqrt{(2+\delta)n}} \mathcal{N}(0,1), \quad i \neq j$$

for any $\delta > 0$, and $[A_n]_{ii} = -1$, then A_n is asymptotically almost surely diagonally stable.

Proof. Consider the random matrix $\bar{A}_n \in \mathbb{R}^{n\times n}$ defined as

$$[\bar{A}_n]_{ij} \sim \frac{1}{\sqrt{(2+\delta)n}} \mathcal{N}(0,1), \quad i \neq j,$$

and $[\bar{A}_n]_{ii} = 0$. Then it follows from (T2) that $\mathbf{Var}[\bar{A}_n]_{ij} = \frac{1}{(2+\delta)n}$ for $i \neq j$. Let $\bar{B}_n = \bar{A}_n + \bar{A}_n^\top$, then it follows that $\mathbf{Var}[\bar{B}_n]_{ij} = \frac{2}{(2+\delta)n}$ when $i \neq j$ and 0 otherwise. From Theorem 3 we have that

$$\begin{aligned} \sup_{1 \le i \le n} \lambda_i(\bar{B}_n) &= 2\sqrt{\frac{2n}{(2+\delta)n}}(1+o(1)) \\ &< 2(1+o(1)) \end{aligned}$$

as $n \to \infty$ asymptotically almost surely. Noting that $A_n^\top + A_n \equiv \bar{B}_n - 2I_{n\times n}$ it follows that $A_n^\top + A_n < 0$ asymptotically almost surely. □

Corollary 18. *If $A_n \in \mathbb{R}^{n\times n}$ i is chosen as*

$$[A_n]_{ij} \sim \mathcal{N}\left(0, \frac{1}{(2+\delta)n}\right), \quad i \neq j,$$

for any $\delta > 0$, and $[A_n]_{ii} = -1$, then A_n is asymptotically almost surely diagonally stable.

5 Clustering Analysis and Ordination Methods

In the following section clustering techniques are explored in detail as well as ordination techniques for visualizing data.

5.1 Distance and Metrics

Let $x = [x_1, x_2, \ldots, x_n]^\top \in \mathbb{R}^n$, then the Euclidian norm is defined as

$$\|x\| = \left(\sum_{i=1}^{n} x_i^2\right)^{1/2}.$$

For a row vector the euclidian norm is similary defined. If no subscript is given with $\|\cdot\|$ then we assume the Euclidian norm is being used. The following is the Euclidian distance function $d(x, y) = \|x - y\|$.

A common distance metric used in ecology is the *Jensen-Shannon Distance* (JSD) metric [21, 27, 29]

$$\mathrm{JSD}(y, z) \triangleq \sqrt{\tfrac{1}{2}\mathrm{JS}(y, z)}, \quad \text{(T40)}$$

where the Jensen-Shanon divergence, $\mathrm{JS}(y, z) \triangleq \mathrm{KL}(y, \frac{1}{2}(y+z)) + \mathrm{KL}(z, \frac{1}{2}(z+y))$, is simply the symetrized version of the Kulback-Liebler directed divergence $\mathrm{KL}(y, z) \triangleq \sum_{i=1}^{p} y_i \log \frac{y_i}{z_i}$. So as to not divide by zero, a pseudo count of 1e-10 is added to zero elements before performing the JSD.

5.2 k-Medoids

Assume that one has a collection of samples $X \in \mathbb{R}^{n\times p}$ where n is the total number of samples and p is the dimension of each sample. Let $X_i \in \mathbb{R}^{1\times p}$, $i = 1, 2, \ldots, n$,

be the row vectors of X as defined below

$$X = \begin{bmatrix} X_1 \\ X_2 \\ \vdots \\ X_n \end{bmatrix}. \tag{T41}$$

We would like each X_j to belong to a unique cluster within a collection of k possible clusters $\mathcal{C}_1, \mathcal{C}_2, \ldots, \mathcal{C}_k$. The unique cluster that contains sample X_j is denoted $\mathcal{C}(j;k)$. If sample X_j is contained in cluster 3 then $\mathcal{C}(j) = \mathcal{C}_3$. If one is given an a-priori number of clusters then it is possible to perform this task using the popular paradigm of k-medoids. The paradigm works as follows. Initially, k samples are chosen at random as representative *medoids*. k clusters are then constructed by associating other samples to the nearest medoid. Within each cluster all elements are tested so as to see if a different sample has a smaller within cluster sum of distances. The element with the smallest within cluster sum of distances is chosen as the new medoid for that cluster. This process is performed for each cluster. New clusters are constructed with the k new medoids and the algorithm repeats again. The MATLAB command `kmedoids` performs k-medoids with a variety of different methods depending on the number of samples. When the number of samples is less than 3000 MATLAB implements k-medoids using the *Partitioning Around Medoids* (PAM) algorithm [24].

5.3 Silhouette Value

For each sample X_j there is a corresponding silhouette value $s_j \in [-1, 1]$ which is defined as follows

$$s_j(k) \triangleq \frac{b_j(k) - a_j(k)}{\max\{a_j(k), b_j(k)\}}$$

where a_j is the average dissimilarity between sample j and all other samples within its own cluster, b_j is the average dissimilarity between X_j and the elements of the nearest cluster, and k is the total number of apriori designated clusters. These two quantities are now formally defined as follows

$$a_j(k) \triangleq \frac{1}{|\mathcal{C}(j;k)| - 1} \sum_{\substack{X_i \in \mathcal{C}(j;k) \\ i \neq j}} d(X_i, X_j).$$

Note that we have used $|\mathcal{C}(j)|$ to denote the cardinality of $\mathcal{C}(j)$, the total number of samples in $\mathcal{C}(j)$. We similarly define the b_j as

$$b_j(k) \triangleq \min_{\mathcal{C}_m \neq \mathcal{C}(j;k)} \frac{1}{|\mathcal{C}_m|} \sum_{X_i \in \mathcal{C}_m} d(X_i, X_j).$$

For a given sample set and a given number of clusters $k \geq 2$ there is a corresponding $s_j(k)$. The Silhouette Index for a sample data set is then the maximum of the mean silhouette value for each total number of clusters.

$$\mathrm{SI}(X) \triangleq \max_k \frac{1}{n} \sum_{j=1}^{n} s_j(k)$$

The optimal number of clusters is then $\arg\max_k \frac{1}{n} \sum_{j=1}^{n} s_j(k)$. Silhouette analysis is performed in MATALAB using the command `silhouette`.

5.4 Variance Ratio Criterion and the Caliński-Harabasz Index

We now define the *Variance Ratio Criterion* (VRC) which holds for any distance function. When the Euclidian metric is used the VRC is referred to as the *Caliński-Harabasz* (CH) index [4]. As before, assume that a collection of samples has already been grouped into k clusters. The VRC is defined as

$$\mathrm{VRC}(k) \triangleq \frac{\mathrm{BG}(k)}{\mathrm{WG}(k)} \frac{n-k}{k-1}$$

where BG is the *Between Group* variance and WG is the *Within Group* variance defined below,

$$\mathrm{BG}(k) \triangleq \sum_{j=1}^{k} \frac{1}{|\mathcal{C}_j| \sum_{m>j}^{k} |\mathcal{C}_m|} \sum_{X_i \in \mathcal{C}_j} \sum_{\substack{X_\ell \in \mathcal{C}_m \\ m>j}} d(X_i, X_\ell)^2$$

$$\mathrm{WG}(k) \triangleq \sum_{j=1}^{k} \frac{2}{|\mathcal{C}_j| \left(|\mathcal{C}_j| - 1\right)} \sum_{\substack{i=1 \\ X_i \in \mathcal{C}_j}}^{n} \sum_{\substack{\ell>i \\ X_\ell \in \mathcal{C}_j}}^{n} d(X_i, X_\ell)^2.$$

5.5 Principle Coordinate Analysis

The purpose of *Principle Coordinates Analysis* (PCoA) is to represent a collection of high dimensional data in a lower dimension. Once again assume that one has a collection of samples $X \in \mathbb{R}^{n \times p}$ where n is total number of samples and p is the dimension of each sample. Let $X_i \in \mathbb{R}^{1 \times p}$, $i = 1, 2, \ldots, n$ be the row vectors of X as defined in (T41). The question answered in this section is how one obtains a $Y \in \mathbb{R}^{n \times k}$, $k \leq n$ with $Y_i \in \mathbb{R}^{1 \times k}$, $i = 1, 2, \ldots, n$ defined as follows

$$Y = \begin{bmatrix} Y_1 \\ Y_2 \\ \vdots \\ Y_n \end{bmatrix}$$

that is a faithful representation of X. We begin by defining the dissimilarity between samples i and j as $\delta_{ij} = d(X_i, X_j)$. Then the goal of this method is to find Y such that $d(Y_i, Y_j)$ is similar to $d(X_i, X_j)$ for the distance measure of interest. Let $D = -\frac{1}{2}(\delta_{ij}^2)_{1 \leq i,j \leq n}$ and

$$B = (I_{n \times n} - n^{-1} \mathbf{1}_n \mathbf{1}_n^\mathsf{T}) D (I_{n \times n} - n^{-1} \mathbf{1}_n \mathbf{1}_n^\mathsf{T}).$$

where $\mathbf{1}_n$ is an n-dimensional column vector with each entry equal to 1. The $n \times k$ dimensional representation of the $n \times p$ sample data is then [37, Chapter 5]

$$Y = [q_1 \sqrt{\lambda_1},\ q_2 \sqrt{\lambda_2},\ \ldots,\ q_k \sqrt{\lambda_k}].$$

where $B = Q \Lambda Q^{-1}$ is the eigenvalue decomposition with eigenvalues $\lambda_i \in \mathbb{R}$, and normalized eigenvectors $q_i \in \mathbb{R}^n$ for $i = 1, 2, \ldots, n$ with $Q = [q_1,\ q_2,\ \ldots,\ q_n]$ and $[\Lambda]_{ii} = \lambda_i$ the diagonal eigenvalue matrix. Due to the fact that B is symmetric all eigenvalues and eigenvectors will be real valued. It is furthermore assumed that

the eigenvalues are arranged such that $\lambda_1 \geq \lambda_2 \geq \cdots \geq \lambda_n$. When the Euclidian distance function is used B is always positive semi-definite. However, it may be possible to have negative eigenvalues when other distance functions are used and thus it is necessary to check and make sure that that λ_i for each $i \in \{1, 2, \ldots, k\}$ is positive for the k-dimensional space of interest. The command that performs this task in MATLAB is `cmdscale`. MATLAB also rotates the data, but that has no affect on the dissimilarity measure, as rotation is distance preserving in Euclidian space.

5.6 Principle Component Analysis

Principle Component Analysis (PCA) is similar in spirit to PCoA in that one wishes to represent a set of data in a lower dimension. Assume that one has a collection of samples $X \in \mathbb{R}^{n \times p}$ where n is total number of samples and p is the dimension of each sample. As before the goal is to find a $Y \in \mathbb{R}^{n \times k}$, $k \leq p$ that is representative of the original data. Assume without loss of generality that the columns of X have mean zero. Let

$$X = U \Sigma W^T$$

be the singular value decomposition of X where $\Sigma \in \mathbb{R}^{n \times p}$ is a diagonal matrix with the singular values $\sigma_i = [\Sigma]_{ii}$ where $\sigma_i \geq \sigma_j$ for $i < j$. The columns of $U \in \mathbb{R}^{n \times n}$ are the left singular vectors and the columns of $W \in \mathbb{R}^{p \times p}$ are the right singular vectors. The reduced order representation is then defined as

$$Y = X W_{1:n,1:k}$$

where $W_{1:n,1:k}$ contains only the first k columns of W. Note that $W_{1:n,1:k}$ can be thought of as a right hand side projection operator and can be used to project subsequent measurements into pre-existing principle components. Also, note that when the Euclidean distance is used in PCoA it is equivalent to PCA. The command that performs this task in MATLAB is `pca`.

6 Modeling

Two approaches for modeling individual human microbial communities are now presented. The first model is the same as that presented in the main text and from this point forward is referred to as the *universal model.* This model assumes that all species interact in a universal manner independent of the host. The second model allows for there to be multiple possible interaction strengths and growth rates for species. This model will be referred to as the *multiple set model.* We use Figure T4 as a visual reference when discussing the modeling paradigms.

6.1 Universal Model

Consider a universal species pool indexed by a set of integers $\mathbf{S} = \{1, \ldots, n\}$, a global $n \times n$ matrix $\mathbf{A}$ representing all possible pairwise interactions between species, and a universal vector $\mathbf{r}$ of size n containing the growth rates for all the n species. The global variables for our ecological system are completely defined by the triple $(\mathbf{S}, \mathbf{A}, \mathbf{r})$. Then q *Local Communities* (LCs) are defined by sets $S^{[\nu]}$,

which are subsets of $\mathbf{S}$ denoting the specific microbes present in LC_ν, $\nu = 1, \ldots, q$. For simplicity we assume that each LC contains only p species ($p \le n$), randomly selected from the universal species pool. The GLV dynamics for each LC is given by

$$\mathrm{LC}_\nu : \quad \dot{x}^{[\nu]}(t) = \mathsf{diag}\left(x^{[\nu]}(t)\right)\left(r^{[\nu]} + A^{[\nu]}x^{[\nu]}(t)\right) \tag{T42}$$

where the LC specific interaction matrix and growth vector are defined as $A^{[\nu]} \triangleq \mathbf{A}_{S^{[\nu]},S^{[\nu]}}$ and $r^{[\nu]} \triangleq \mathbf{r}_{S^{[\nu]}}$, respectively. That is, $A^{[\nu]}$ is obtained from $\mathbf{A}$ by only taking the rows and columns of $\mathbf{A}$ that are contained in the set $S^{[\nu]}$. A similar

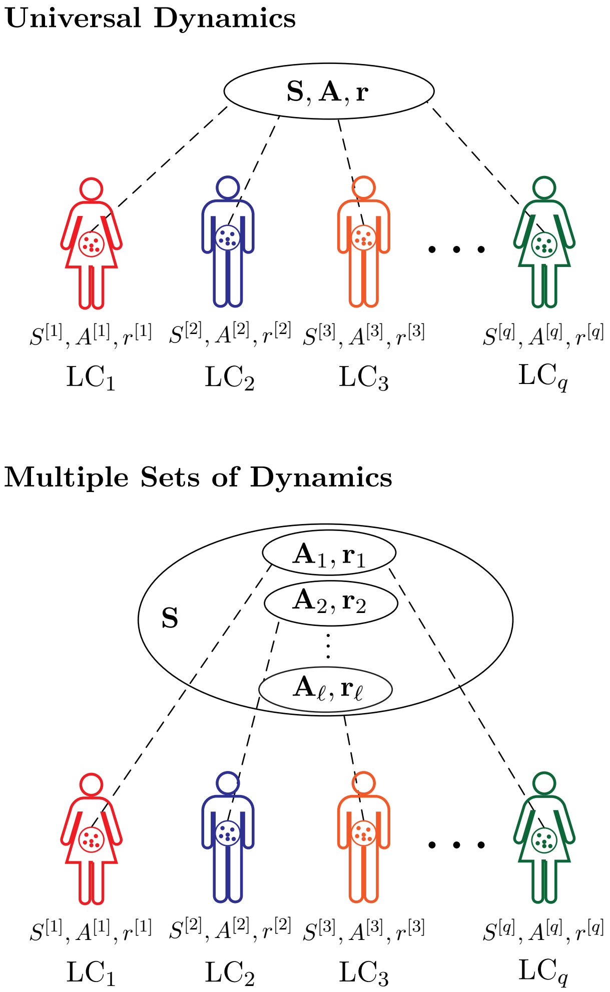

Supplementary Text Figure T4: *Modeling Paradigms.* Two different modeling paradigms are presented. In the *universal* paradigm there is a universal list of species $\mathbf{S}$, a universal interaction matrix $\mathbf{A}$, containing all the pairwise interaction strengths, and a universal growth rate vector $\mathbf{r}$. Then the dynamics of each Local Community LC is determined by the collection of species in that community. In the *multiple set* paradigm the dynamics are not only determined by the collection of species present but the dynamics are not universal and come from a collection of possible dynamics. In the example above LC_1 dynamics are derived from the first dynamic pair $(\mathbf{A}_1, \mathbf{r}_1)$, LC_2 dynamics are derived from $(\mathbf{A}_2, \mathbf{r}_2)$, LC_3 dynamics are derived from $(\mathbf{A}_\ell, \mathbf{r}_\ell)$, and LC_q dynamics are derived from the dynamic pair $(\mathbf{A}_1, \mathbf{r}_1)$.

procedure is performed in order to obtain $r^{[\nu]}$. Finally there is a map m_ν that takes the abundances $x^{[\nu]}$ in the index $S^{[\nu]}$ and carries them to the universal index in $\mathbf{S}$ giving the vector $\mathbf{x}^{[\nu]} = m_\nu\left(x^{[\nu]}\right)$ which results in $\mathbf{x}^{[\nu]}_{S^{[\nu]}_j} = x^{[\nu]}_j$ for $j = 1, \ldots, p$ and $\mathbf{x}^{[\nu]}_j = 0$ for j in $\mathbf{S} \setminus S^{[\nu]}$. This modeling procedure is inspired by [7]. A toy example of how this model is used to construct a single local community is now given

Example 1. Consider a global set of 4 species and thus $\mathbf{S} = \{1, 2, 3, 4\}$. Let

$$\mathbf{A} = \begin{bmatrix} -1 & 0.1 & 0.4 & -0.1 \\ 0.7 & -1 & 0 & 0.4 \\ -0.1 & 0.7 & -1 & 0 \\ -0.8 & -0.2 & 0.4 & -1 \end{bmatrix} \quad \text{and} \quad \mathbf{r} = \begin{bmatrix} 0.2 \\ 0.4 \\ 0.5 \\ 0.4 \end{bmatrix}.$$

Then, if the first local community has the species list $S^{[1]} = \{1, 3, 4\}$ then it follows that

$$A^{[1]} = \begin{bmatrix} -1 & 0.4 & -0.1 \\ -0.1 & -1 & 0 \\ -0.8 & 0.4 & -1 \end{bmatrix} \quad \text{and} \quad r^{[1]} = \begin{bmatrix} 0.2 \\ 0.5 \\ 0.4 \end{bmatrix}.$$

6.2 Mutiple Set Model

As before there is a universal set of species $\mathbf{S} = \{1, \ldots, n\}$, but now there are ℓ pairs of possible global dynamics $\{(\mathbf{A}_1, \mathbf{r}_1), (\mathbf{A}_2, \mathbf{r}_2), \ldots, (\mathbf{A}_\ell, \mathbf{r}_\ell)\}$. The q LCs are defined by sets $S^{[\nu]}$, $\nu = 1, \ldots, q$ and a map $w : \{1, 2, \ldots, q\} \to \{1, 2, \ldots, \ell\}$ which determines which model pair the LC is derived from. The GLV dynamics for each LC are given by (T42) where $A^{[\nu]} \triangleq [\mathbf{A}_{w(\nu)}]_{S^{[\nu]}, S^{[\nu]}}$ and $r^{[\nu]} \triangleq [\mathbf{r}_{w(\nu)}]_{S^{[\nu]}}$, respectively. That is, $A^{[\nu]}$ is obtained from $\mathbf{A}_{w(\nu)}$ by only taking the rows and columns of $\mathbf{A}_{w(\nu)}$ that are contained in the set $S^{[\nu]}$. A similar procedure is performed in order to obtain $r^{[\nu]}$. The map m_ν that takes the abundances $x^{[\nu]}$ in the index $S^{[\nu]}$ and carries them to the universal index $\mathbf{S}$ is defined as before $\mathbf{x}^{[\nu]} = m_\nu(x^{[\nu]})$. It is worth noting that results from this modeling paradigm are not presented in the main text, because the emergence of community types in this case is trivial (see §7.1) [26].

7 Simulation Results and Analysis

We are finally in a position to address the main topic of this work. Revealing properties that allow for community types to arise. Four case studies are now presented. The first case study will use the multiple set model paradigm in Seciton 6.2, and it will be shown that under this paradigm that clustering of the steady states occurs trivially. The subsequent three case studies use the universal model from Section 6.1. The case studies explore heterogeneity (in terms of interaction strength and network topology), mean degree of the network, and community overlap and how these affect the existence of community types for our dynamics of interest.

7.1 Multiple Set Models

For the multiple set model study three universal pairs $\{(\mathbf{A}_1, \mathbf{r}_1), (\mathbf{A}_2, \mathbf{r}_2), (\mathbf{A}_3, \mathbf{r}_3)\}$ were considered. For this study there was a total of 100 global species. Each $\mathbf{A}_i$

was a 100 by 100 matrix with values drawn from a normal distribution with mean 0 and variance 0.0049. Then the diagonal elements of each matrix are set to -1, that is $[\mathbf{A}_i]_{jj} = -1$, $i = 1, 2, 3$, $j = 1, 2, \ldots, 100$. Note that with the above selection, the variance of the off diagonal elements of $\mathbf{A}_i$ satisfy the following bound $0.0049 < \frac{1}{2 \cdot 100}$, which from Corollary 18 is the sufficient condition on the variance for asymptotic almost sure diagonal stability when $n = 100$. Also, note the interaction network is implicitly a complete graph without any structural heterogeneity. Finally, each $\mathbf{r}_i$ had elements drawn from a uniform distribution between 0 and 1.

From the three pairs above 600 local communities were constructed, 200 from each universal pair. Each of the local communities contained 80 species. The dynamics were then simulated for 100 seconds with initial conditions drawn from $\mathcal{U}(0, 1)$. The abundances of the species in the communities were then normalized, and the relative abundances of the 600 local communities were clustered using k-medoids from Section 5.2 and silhouette indexed as defined in Section 5.3, each using the Jensen-Shannon metric in (T40). A principle coordinate plot of the results is given in Figure T5 with the elements colored according to cluster assignment from k-medoids. It is clear that there are three clusters. Furthermore each cluster exactly coincides with the universal $\mathbf{A}_i$ that the local community dynamics were obtained from.

7.2 Universal Model

For the three universal case studies the global interaction matrix is defined as

$$\mathbf{A} = \mathbf{N}\mathbf{H} \circ \mathbf{G}s,$$

which contains four components.

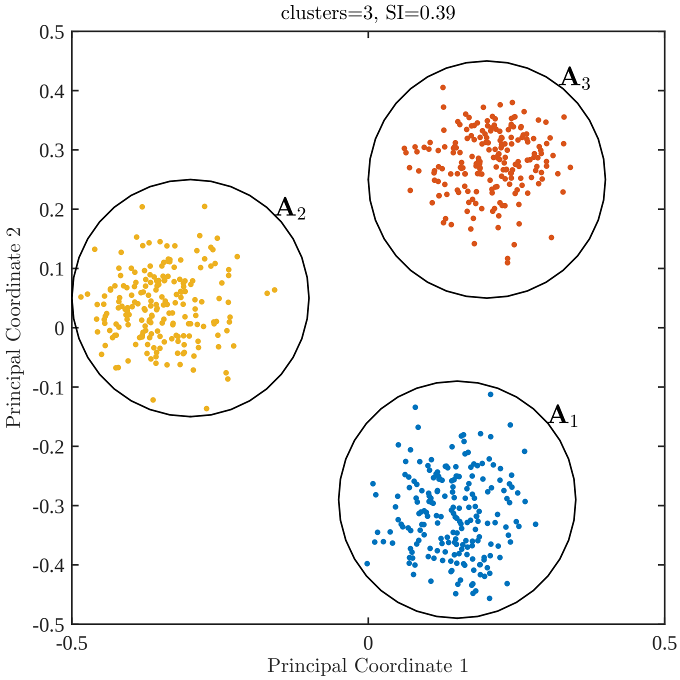

Supplementary Text Figure T5: Principle coordinates for multiple model case study.

(i) $\mathbf{N} \in \mathbb{R}^{n\times n}$ is the nominal component where each element is sampled from either a normal distribution or a uniform distribution.

(ii) The matrix $\mathbf{H}$ is a diagonal matrix that captures the overall interaction heterogeneity of different species. When interaction strength heterogeneity is employed the diagonal elements of $\mathbf{H}$ are drawn from a power-law distribution (T3) with exponent $-\alpha$, $[\mathbf{H}]_{ii} \sim \mathcal{P}(\alpha)$, which are subsequently normalized so that the mean of the diagonal is equal to 1. Without interaction heterogeneity $\mathbf{H}$ is simply the identity matrix.

(iii) The matrix $\mathbf{G}$ is the adjacency matrix of the underlying ecological network: $[\mathbf{G}]_{ij} = 1$ if species i is affected by the presence of species j and 0 otherwise. When the underlying network is a complete digraph all elements in $\mathbf{G}$ are equal to 1. For details on the construction of $\mathbf{G}$ when the underlying network is Erdős-Rényi or a power-law digraph see Sections 3.2.1 and 3.2.2 respectively.

(iv) The last element s is simply a scaling factor between 0 and 1.

As before we set $[\mathbf{A}]_{ii} = -1$ to ensure one of the necessary conditions for diagonal stability of a matrix is satisfied (Theorem 10 and Corollary 11). Finally, the elements in the global growth rate vector are defined as

$$\mathbf{r} = \mathbf{h} \circ \mathbf{n}$$

where $\mathbf{n}$ is the nominal component taken from a uniform distribution, and $\mathbf{h}$ captures the growth rate heterogeneity. When there is growth rate heterogeneity $\mathbf{h}$ is drawn from a power-law distribution with exponent $-\alpha$ and subsequently normalized to have a mean of 1. Without growth rate heterogeneity $\mathbf{h}$ is simply a column vector of ones. Note that $\mathbf{h}$ is not included in the main text, and is only used in one scenario of one case study within this section.

7.2.1 Universal Model: Heterogeneity Study

Next is a systematic study of heterogeneity and its effects on the dynamics in the universal model. Eight different scenarios were tested in this study. For each of the following scenarios the number of global species is $n = 100$. Table T1 outlines the differences between the scenarios.

Scenario 1. $[\mathbf{N}]_{ij} \sim \mathcal{U}(-0.5, 0.5)$, $[\mathbf{H}]_{ii} \sim \mathcal{P}(\alpha)$ where $\alpha \in [1.2, 7]$ and subsequently normalized to have a mean of 1, $\mathbf{G}$ is the adjacency matrix for a complete digraph and thus all entries are equal to 1, and the scaling factor is set to $s = 0.07$. There is no growth rate heterogeneity and thus the column vector $\mathbf{h}$ is all ones and finally $[\mathbf{n}]_i \sim \mathcal{U}(0, 1)$.

Scenario 2. The same as Scenario 1 but with $[\mathbf{N}]_{ij} \sim \mathcal{N}(0, 1)$ and $s = 0.07$.

Scenario 3. $[\mathbf{N}]_{ij} \sim \mathcal{U}(-0.5, 0.5)$, $[\mathbf{H}]_{ii} \sim \mathcal{P}(\alpha)$ where $\alpha \in [1.2, 7]$ and subsequently normalized to have a mean of 1, $\mathbf{G}$ is the adjacency matrix for an Erdős-Rényi digraph with a mean out-degree of 10, and the scaling factor is set to $s = 0.5$. There is no growth rate heterogeneity and thus the column vector $\mathbf{h}$ is all ones and finally $[\mathbf{n}]_i \sim \mathcal{U}(0, 1)$. For more details on Erdős-Rényi digraphs see §3.2.1.

Scenario 4. The same as Scenario 3 but with $[\mathbf{N}]_{ij} \sim \mathcal{N}(0,1)$ and $s = 0.1$.

Scenario 5. $[\mathbf{N}]_{ij} \sim \mathcal{N}(0,1)$, $\mathbf{H}$ is the identity matrix, $\mathbf{G}$ is the adjacency matrix for a digraph with the out-degree drawn from a power-law distribution $\mathcal{P}(\alpha)$ with a mean out-degree of 10 where $\alpha \in [1.2, 7]$, and the scaling factor is set to $s = 0.2$. There is no growth rate heterogeneity and thus the column vector $\mathbf{h}$ is all ones and $[\mathbf{n}]_i \sim \mathcal{U}(0,1)$. For more details on power-law out-degree digraphs see §3.2.2.

Scenario 6. $[\mathbf{N}]_{ij} \sim \mathcal{N}(0,1)$, $[\mathbf{H}]_{ii} \sim \mathcal{P}(\alpha)$ where $\alpha \in [1.2, 7]$ and subsequently normalized to have a mean of 1, $\mathbf{G}$ is the adjacency matrix for a digraph with the out-degree drawn from a power-law distribution $\mathcal{P}(\alpha)$ with a mean out-degree of 10 where $\alpha \in [1.2, 7]$, and the scaling factor is set to $s = 0.1$. The power-law degree distribution and interaction strength heterogeneity are uncoupled. There is no growth rate heterogeneity and thus the column vector $\mathbf{h}$ is all ones and $[\mathbf{n}]_i \sim \mathcal{U}(0,1)$. For more details on power-law out-degree digraphs see §3.2.2.

Scenario 7. $[\mathbf{N}]_{ij} \sim \mathcal{N}(0,1)$, $[\mathbf{H}]_{ii} \sim \mathcal{P}(\alpha)$ where $\alpha \in [1.2, 7]$ and subsequently normalized to have a mean of 1, $\mathbf{G}$ is the adjacency matrix for a digraph with the out-degree drawn from a power-law distribution $\mathcal{P}(\alpha)$ with a mean out-degree of 10 where $\alpha \in [1.2, 7]$, and the scaling factor is set to $s = 0.02(\alpha + 1)$. The power-law degree distribution and interaction strength heterogeneity are coupled so that the node with the highest out-degree is also the node with the largest interaction strength scaling. There is no growth rate heterogeneity and thus the column vector $\mathbf{h}$ is all ones and $[\mathbf{n}]_i \sim \mathcal{U}(0,1)$. For more details on power-law out-degree digraphs see §3.2.2.

Scenario 8. $[\mathbf{N}]_{ij} \sim \mathcal{N}(0,1)$, $\mathbf{H}$ is the identity matrix, $\mathbf{G}$ is the adjacency matrix for an Erdős-Rényi digraph with a mean out-degree of 10, and the scaling factor is set to $s = 0.1$. There is growth rate heterogeneity and thus the column vector $\mathbf{h}$ has elements drawn from a power-law distribution $\mathcal{P}(\alpha)$ and subsequently normalized to have a mean of 1 and $[\mathbf{n}]_i \sim \mathcal{U}(0,1)$. For more details on Erdős-Rényi digraphs see §3.2.1.

For each of the eight scenarios above and for every α, $q = 500$ local communities were generated each with $p = 80$ species selected at random from the $n = 100$ global species. The dynamics as described above following the modeling paradigm in Section 6.1 were then simulated for 100 seconds with initial conditions drawn from $\mathcal{U}(0,1)$. If any of the 500 simulations crashed due to instability or if the norm of the terminal discrete time derivative was greater than 0.01 then that local community was excluded from the rest of the study. Those simulations that finished without crashing and with small terminal discrete time derivative were deemed steady. Less than 1% of simulations were deemed unstable. The abundances of the species in the communities were then normalized, and the relative abundances of the 500 local communities were clustered using k-medoids from Section 5.2 and silhouette indexed as defined in Section 5.3, each using the Jensen-Shannon distance metric in (T40).

The results from the above scenarios are presented in Figures T6-T13. The first plot is a comprehensive clustering analysis of the steady state values obtained from the simulations. The x-axis denotes the heterogeneity value α. The box plots are the silhouette values pertaining to the number of clusters for which the silhouette index was defined. The total number of clusters pertaining to the silhouette index is denoted on the top x-axis. The second row is a principle coordinate analysis of the steady state values obtained at three different heterogeneity values. Clusters are color coded to match the optimal clustering from k-medoids. The third row of the figure plots

$$\max_{i,j} \text{real}\left(\lambda_i(A^{[j]})\right)$$

as a function of α. The fourth row is a plot of $\lambda_i(A^{[j]})$ for $i \in \{1, 2, \ldots, 80\}$ and $j \in \{1, 2, \ldots, 500\}$ at three values of α.

Figures T6 and T7 show that regardless of whether the nominal component is drawn from a uniform or a normal distribution the increase in interaction strength heterogeneity (decreasing α) leads to steady state clustering in the data. Figures T8 and T9 illustrate that the same phenomenon also holds for Erdős-Rényi digraphs as well. When the underlying graph topology follows a power-law degree distribution, but there is no interaction strength heterogeneity, clustering of steady states is not observed, see Figure T10. Figure T11 illustrates the fact that when there is interaction strength heterogeneity and network degree heterogeneity it is possible to have clustering of steady states when α is in the range $[3, 1]$. However the trend is not smooth and is inconsistent, as compared to Figures T6-T9. This is due to the fact that when the underlying interaction network is being constructed, there is no guarantee that the high-degree node will also have a large interaction strength. For instance, if a node with no out edges is randomly selected to have high interaction strength scaling, then the impact of that node on the rest of the nodes is still zero. When the interaction strength heterogeneity is coupled with the out-degree for a power-law out-degree digraph then the trends from Figures T6-T9 are recovered. Finally, if the growth rates of the species are derived from a power-law distribution, then clustering of steady states also occurs as α decreases.

Rows three and four illustrate the spectrum of $A^{[j]}$, and are included so that we can infer the asymptotic stability of the system for certain paradigms. Note that regardless of whether the nominal interactions are drawn from a normal distribution or a uniform distribution when α is large the spectrum represents a uniform disk in the complex plain as predicted by Theorem 1. Furthermore, for scenario 2 with $s = 0.07$ and $[\mathbf{N}]_{ij} \sim \mathcal{N}(0, 1)$ it follows that $\mathbf{Var}[\mathbf{A}]_{ij} < \frac{1}{\sqrt{2n}}$ for $n = 100$. From Theorem 17 it follows that $\mathbf{A}$ is diagonally stable, in a probabilistic sense. Then invoking Theorem 10 we know that any principle minor of $\mathbf{A}$ is diagonally stable as well. Therefore, in Scenario 2 for large α each $A^{[j]}$ is diagonally stable, in a probabilistic sense.

Also, for all of the scenarios with interaction heterogeneity all of the eigenvalues of $\mathbf{A}$, and consequently $A^{[j]}$, converge to -1 as α tends to 1. In the limit of α tending to 1, only one of the columns of $\mathbf{A}$ has non-zero values off the diagonal. Therefore, in the limit of α tending to 1 the following inequality holds $\mathbf{A}^\mathsf{T} P + P\mathbf{A} < 0$ where $P = [1, \epsilon, \ldots, \epsilon]^\mathsf{T}$ and ϵ is sufficiently small.[5] Thus $\mathbf{A}$ is diagonally stable in

[5] Without loss of generality we have assumed that the first column of $\mathbf{A}$ is the highly weighted

the limit of α tending to 1. Therefore, for low interaction strength heterogeneity and for high interaction strength heterogeneity one of the necessary conditions for uniform asymptotic stability in the positive orthant is satisfied, see Theorem 5. Note that even when the $A^{[j]}$ are not diagonally stable it does not imply that the state trajectory $x^{[j]}(t)$ is unstable.

7.2.2 Universal Model: Sparsity Study

Next is a study where the mean degree of the Erdős-Rényi digraph along with the interaction strength heterogeneity is varied. Table T2 outlines the differences between the scenarios. The details of the study are as follows: $[\mathbf{N}]_{ij} \sim \mathcal{U}(-0.5, 0.5)$, $[\mathbf{H}]_{ii} \sim \mathcal{P}(\alpha)$ where $\alpha \in [1.2, 7]$ and subsequently normalized to have a mean of 1, $\mathbf{G}$ is the adjacency matrix for an Erdős-Rényi digraph with a mean out-degree of

$$d \in \{1,\ 3,\ 5,\ 7,\ 9,\ 11,\ 13,\ 15,\ 17,\ 19\},$$

and the scaling factor is set to $s = 1/\sqrt{d}$. There is no growth heterogeneity and thus the column vector $\mathbf{h}$ is all ones and finally $[\mathbf{n}]_i \sim \mathcal{U}(0, 1)$. For each of the ten scenarios above and for every α the same procedure as in §7.2.1 was carried out and the results are shown in Figures T14-T23.

From Figures T14-T23 it can be concluded that so long as the mean out-degree of the ER digraph is greater than 2 the steady states of the GLV model increase in SI as α decreases. That is, the same trends as observed in the previous study hold, so long as the ER digraph is connected. When the mean degree is 1 for an ER graph it is very unlikely that the graph will be connected [13]. Thus for a digraph with mean out-degree 1, it is even more unlikely that it will be connected. When the underlying digraph $\mathbf{G}$ has many isolated nodes then the scaling of interaction strengths does not influence as many other species in the GLV system and thus clustering is not observed.

7.2.3 Universal Model: Community Size Study

In all previous studies each local community contained 80 species. For this study the size of the local communities take on values in the following set

$$p \in \{100,\ 99,\ 95,\ 90,\ 80,\ 70,\ 60,\ 50\}.$$

Details of the study are as follows: $[\mathbf{N}]_{ij} \sim \mathcal{U}(-0.5, 0.5)$, $[\mathbf{H}]_{ii} \sim \mathcal{P}(\alpha)$ where $\alpha \in [1.2, 7]$ and subsequently normalized to have a mean of 1, $\mathbf{G}$ is the adjacency matrix for an Erdős-Rényi digraph with a mean out-degree of 10, and the scaling factor is set to $s = 0.5$. There is no growth heterogeneity and thus the column vector $\mathbf{h}$ is all ones and finally $[\mathbf{n}]_i \sim \mathcal{U}(0, 1)$. Table T3 outlines the differences between the scenarios. For each of the eight scenarios above and for every α the same procedure as in §7.2.1 was carried out and the results are shown in Figures T24-T31.

These results show that the trends observed in the earlier studies still hold when the community sizes are varied between 95 and 50 species, Figures T26-T31. As the community sizes approach 50 the trend of increased clustering with increased heterogeneity is less significant. When the number of species in the LCs approaches

column.

the number of species in the meta-community, 100, the results do not follow the same trends as before and the Silhouette Indices are near 1, independent of the interaction strength heterogeneity. In Figure T24 all the simulations are identical, each LC has the same 100 species but with different initial conditions. Due to asymptotic stability all of the simulations converge to the same steady state (only those simulations for α between 2.2 and 3 have the potential to be unstable). Even though the Silhouette Indices are near 1 across the heterogeneity spectrum, all of the steady state values are within 10^{-3} to 10^{-2} in terms of the first two principle coordinates. When all of the LCs differ by only one species, Figure T25, once again the Silhouette Indices are near 1, yet the PCoA illustrates that most of the samples are very close together with just a few outliers. Figures T24 and T25 illustrate the sometimes confounding results when clustering analysis and PCoA are performed [20, 32].

Table T1: Parameter settings for heterogeneity case study.

Scenario	Growth Rate Distribution	Nominal Interaction Distribution	Interaction Structure	Mean Degree	Interaction Strength Heterogeneity	Network Structure Heterogeneity	Growth Rate Heterogeneity
1	uniform	uniform	complete	100	$\alpha \in [1.2,\ 7]$	none	none
2	uniform	normal	complete	100	$\alpha \in [1.2,\ 7]$	none	none
3	uniform	uniform	Erdős-Rényi	10	$\alpha \in [1.2,\ 7]$	none	none
4	uniform	normal	Erdős-Rényi	10	$\alpha \in [1.2,\ 7]$	none	none
5	uniform	normal	power-law	10	none	$\alpha \in [1.2,\ 7]$	none
6	uniform	normal	power-law	10	$\alpha \in [1.2,\ 7]$	$\alpha \in [1.2,\ 7]$	none
7	uniform	normal	power-law	10	$\alpha \in [1.2,\ 7]$	$\alpha \in [1.2,\ 7]$(coupled)	none
8	uniform	normal	Erdős-Rényi	100	none	none	$\alpha \in [1.2,\ 7]$

Table T2: Parameter settings for sparsity study.

Scenario	Growth Rate Distribution	Nominal Interaction Distribution	Interaction Structure	Mean Degree	Interaction Strength Heterogeneity	Network Structure Heterogeneity	Growth Rate Heterogeneity
1	uniform	uniform	Erdős-Rényi	1	$\alpha \in [1.2,\ 7]$	none	none
2	uniform	uniform	Erdős-Rényi	3	$\alpha \in [1.2,\ 7]$	none	none
3	uniform	uniform	Erdős-Rényi	5	$\alpha \in [1.2,\ 7]$	none	none
4	uniform	uniform	Erdős-Rényi	7	$\alpha \in [1.2,\ 7]$	none	none
5	uniform	uniform	Erdős-Rényi	9	$\alpha \in [1.2,\ 7]$	none	none
6	uniform	uniform	Erdős-Rényi	11	$\alpha \in [1.2,\ 7]$	none	none
7	uniform	uniform	Erdős-Rényi	13	$\alpha \in [1.2,\ 7]$	none	none
8	uniform	uniform	Erdős-Rényi	15	$\alpha \in [1.2,\ 7]$	none	none
9	uniform	uniform	Erdős-Rényi	17	$\alpha \in [1.2,\ 7]$	none	none
10	uniform	uniform	Erdős-Rényi	19	$\alpha \in [1.2,\ 7]$	none	none

Table T3: Parameter settings for community size study.

Scenario	Growth Rate Distribution	Nominal Interaction Distribution	Interaction Structure	Mean Degree	Interaction Strength Heterogeneity	Network/Growth Heterogeneity	Community Size
1	uniform	uniform	Erdős-Rényi	10	$\alpha \in [1.2,\ 7]$	none	100
2	uniform	uniform	Erdős-Rényi	10	$\alpha \in [1.2,\ 7]$	none	99
3	uniform	uniform	Erdős-Rényi	10	$\alpha \in [1.2,\ 7]$	none	95
4	uniform	uniform	Erdős-Rényi	10	$\alpha \in [1.2,\ 7]$	none	90
5	uniform	uniform	Erdős-Rényi	10	$\alpha \in [1.2,\ 7]$	none	80
6	uniform	uniform	Erdős-Rényi	10	$\alpha \in [1.2,\ 7]$	none	70
7	uniform	uniform	Erdős-Rényi	10	$\alpha \in [1.2,\ 7]$	none	60
8	uniform	uniform	Erdős-Rényi	10	$\alpha \in [1.2,\ 7]$	none	50

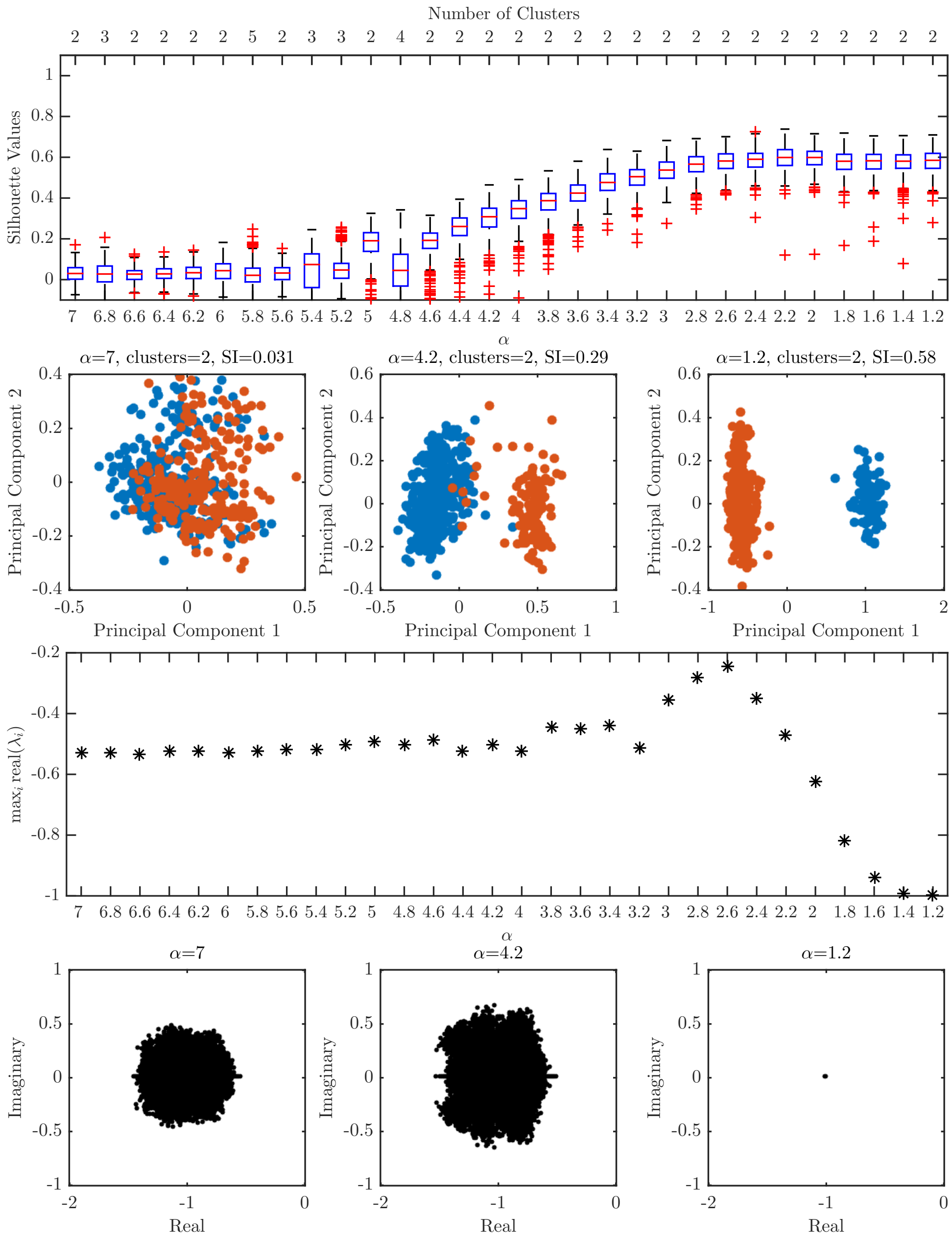

Supplementary Text Figure T6: Universal Model Heterogeneity Study Scenario 1 in Table T1. The first plot is a comprehensive clustering analysis of the steady state values obtained from the Lotka-Volterra simulations. The x-axis denotes the heterogeneity value α. The box plots are the silhouette values pertaining to the number of clusters for which the silhouette index (maximum over the number of clusters of the mean silhouette value for each given total number of cluster) was defined. The total number of clusters pertaining to the silhouette index is denoted on the top x-axis. The second row is a principle coordinate analysis of the steady state values obtained at three different heterogeneity values. Clusters are color coded to match the optimal clustering from k-medoids. The third row of the figure plots $\max_{i,j} \text{real}(\lambda_i(A^{[j]}))$ as a function of α. The fourth row is a plot of $\lambda_i(A^{[j]})$for $i \in \{1, 2, \ldots, 80\}$ and $j \in \{1, 2, \ldots, 500\}$ at three values of α.

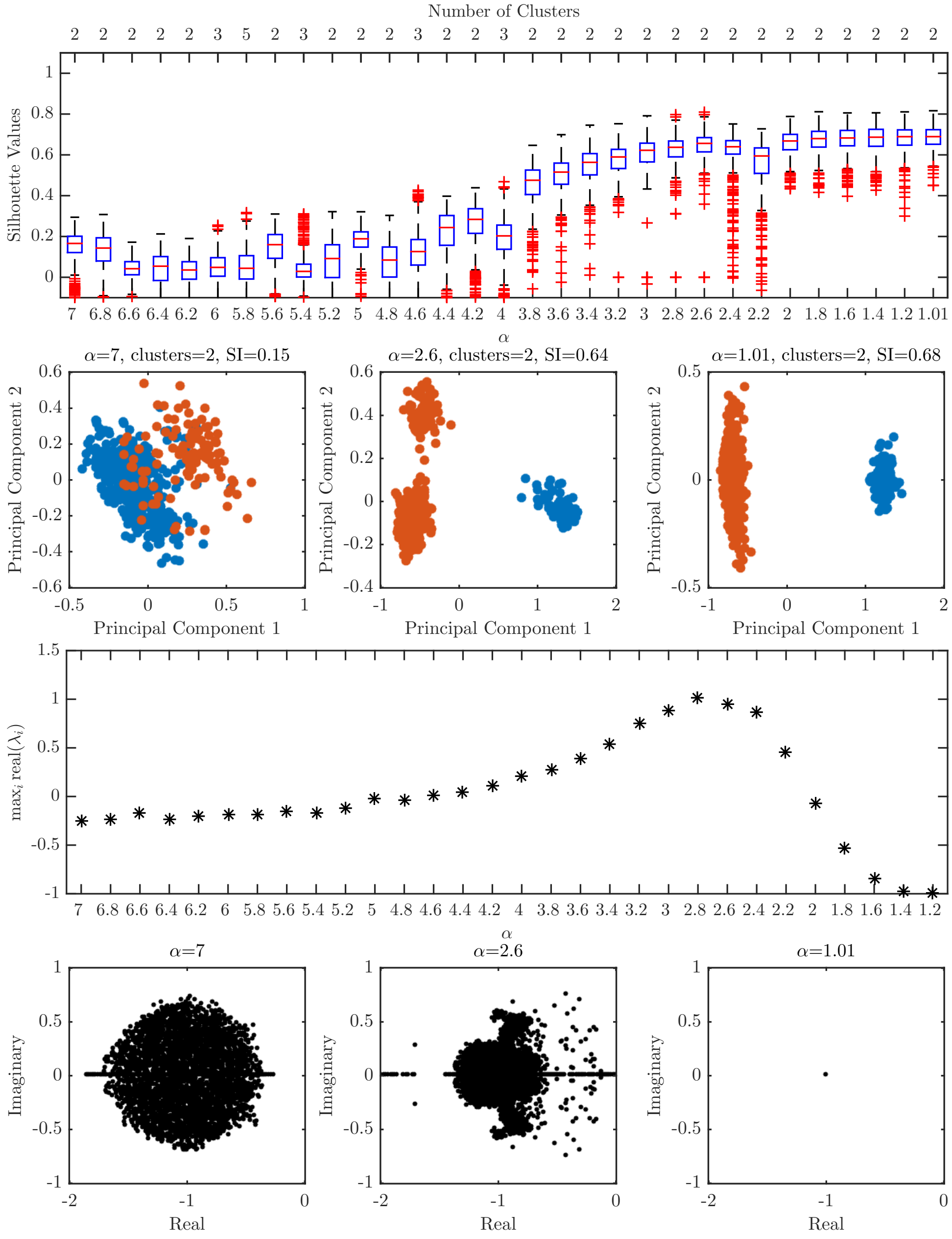

Supplementary Text Figure T7: Universal Model Heterogeneity Study Scenario 2 in Table T1. The first plot is a comprehensive clustering analysis of the steady state values obtained from the Lotka-Volterra simulations. The x-axis denotes the heterogeneity value α. The box plots are the silhouette values pertaining to the number of clusters for which the silhouette index (maximum over the number of clusters of the mean silhouette value for each given total number of cluster) was defined. The total number of clusters pertaining to the silhouette index is denoted on the top x-axis. The second row is a principle coordinate analysis of the steady state values obtained at three different heterogeneity values. Clusters are color coded to match the optimal clustering from k-medoids. The third row of the figure plots $\max_{i,j}$ real$(\lambda_i(A^{[j]}))$ as a function of α. The fourth row is a plot of $\lambda_i(A^{[j]})$for $i \in \{1, 2, \ldots, 80\}$ and $j \in \{1, 2, \ldots, 500\}$ at three values of α.

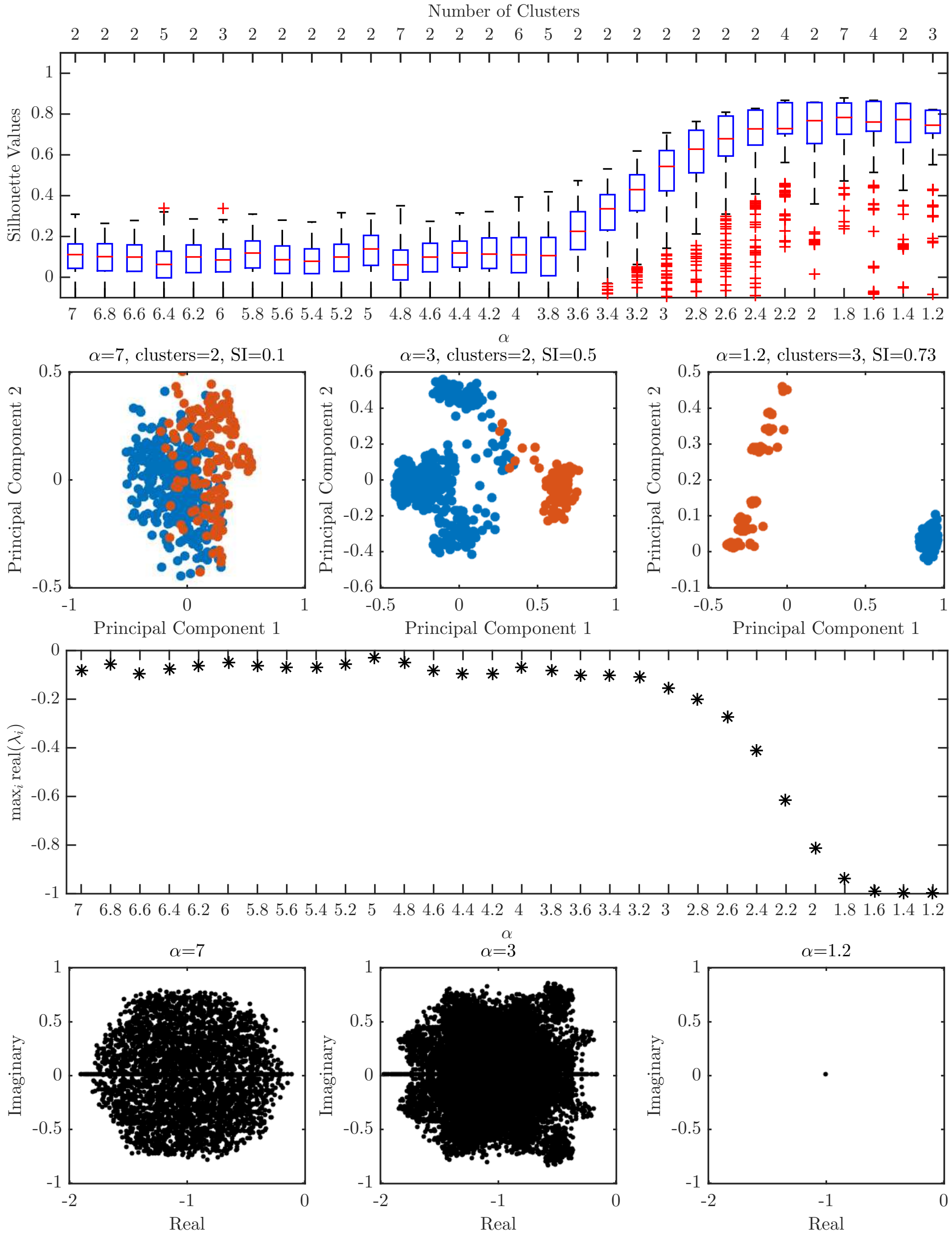

Supplementary Text Figure T8: Universal Model Heterogeneity Study Scenario 3 in Table T1. The first plot is a comprehensive clustering analysis of the steady state values obtained from the Lotka-Volterra simulations. The x-axis denotes the heterogeneity value α. The box plots are the silhouette values pertaining to the number of clusters for which the silhouette index (maximum over the number of clusters of the mean silhouette value for each given total number of cluster) was defined. The total number of clusters pertaining to the silhouette index is denoted on the top x-axis. The second row is a principle coordinate analysis of the steady state values obtained at three different heterogeneity values. Clusters are color coded to match the optimal clustering from k-medoids. The third row of the figure plots $\max_{i,j} \text{real}(\lambda_i(A^{[j]}))$ as a function of α. The fourth row is a plot of $\lambda_i(A^{[j]})$for $i \in \{1, 2, \ldots, 80\}$ and $j \in \{1, 2, \ldots, 500\}$ at three values of α.

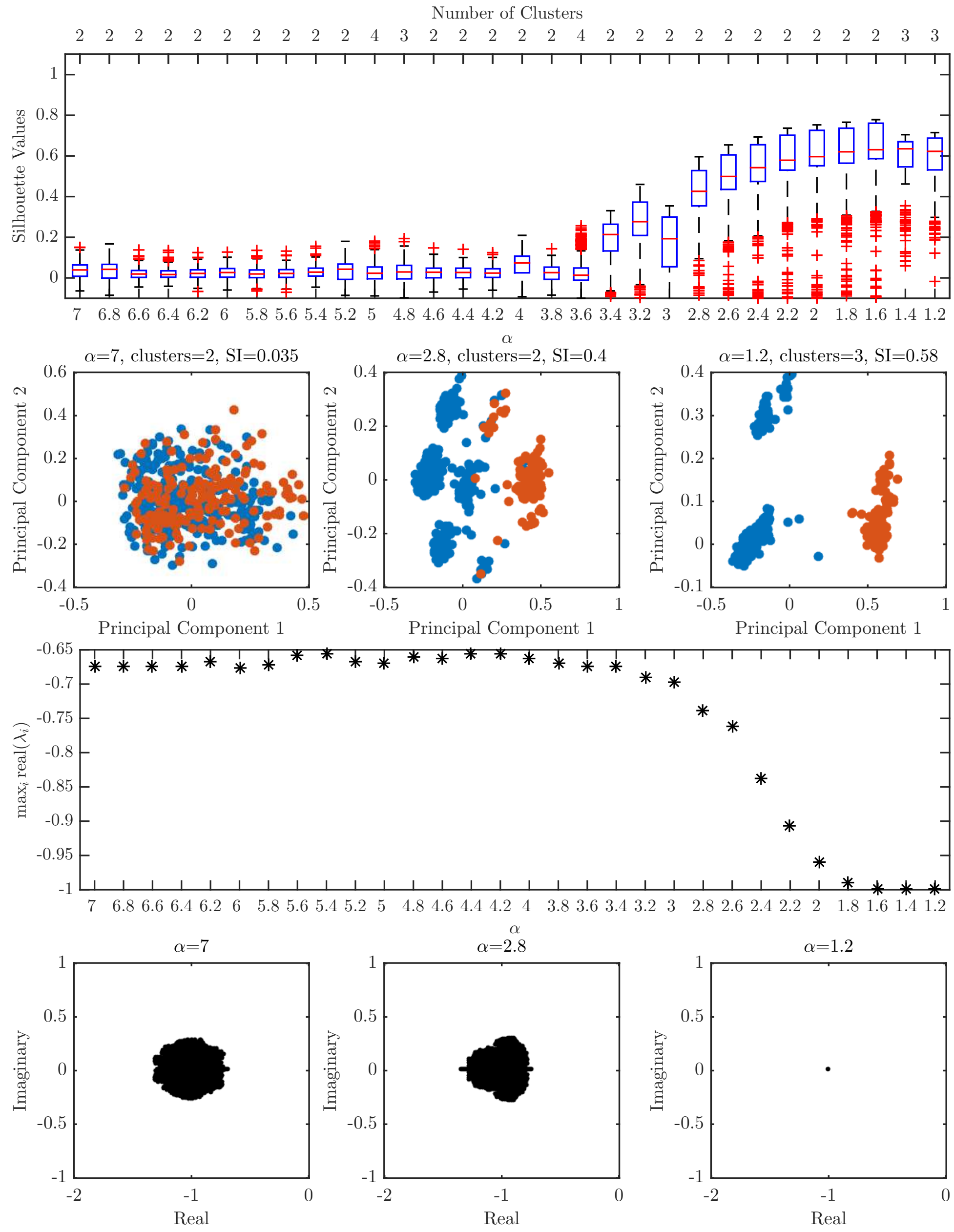

Supplementary Text Figure T9: Universal Model Heterogeneity Study Scenario 4 in Table T1. The first plot is a comprehensive clustering analysis of the steady state values obtained from the Lotka-Volterra simulations. The x-axis denotes the heterogeneity value α. The box plots are the silhouette values pertaining to the number of clusters for which the silhouette index (maximum over the number of clusters of the mean silhouette value for each given total number of cluster) was defined. The total number of clusters pertaining to the silhouette index is denoted on the top x-axis. The second row is a principle coordinate analysis of the steady state values obtained at three different heterogeneity values. Clusters are color coded to match the optimal clustering from k-medoids. The third row of the figure plots $\max_{i,j}\,\mathrm{real}(\lambda_i(A^{[j]}))$ as a function of α. The fourth row is a plot of $\lambda_i(A^{[j]})$for $i \in \{1, 2, \ldots, 80\}$ and $j \in \{1, 2, \ldots, 500\}$ at three values of α.

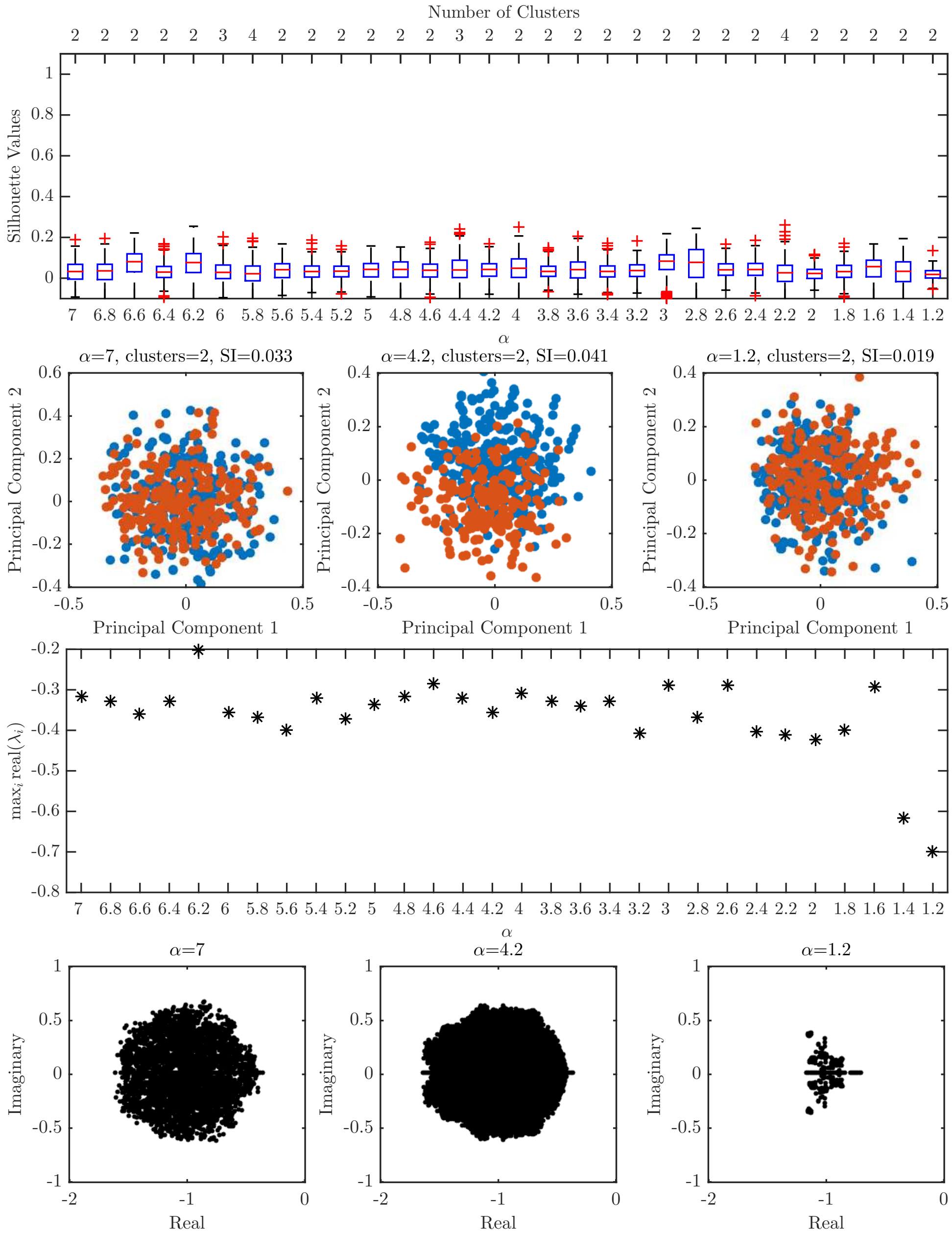

Supplementary Text Figure T10: Universal Model Heterogeneity Study Scenario 5 in Table T1. The first plot is a comprehensive clustering analysis of the steady state values obtained from the Lotka-Volterra simulations. The x-axis denotes the heterogeneity value α. The box plots are the silhouette values pertaining to the number of clusters for which the silhouette index (maximum over the number of clusters of the mean silhouette value for each given total number of cluster) was defined. The total number of clusters pertaining to the silhouette index is denoted on the top x-axis. The second row is a principle coordinate analysis of the steady state values obtained at three different heterogeneity values. Clusters are color coded to match the optimal clustering from k-medoids. The third row of the figure plots $\max_{i,j}$ real$(\lambda_i(A^{[j]}))$ as a function of α. The fourth row is a plot of $\lambda_i(A^{[j]})$for $i \in \{1, 2, \ldots, 80\}$ and $j \in \{1, 2, \ldots, 500\}$ at three values of α.

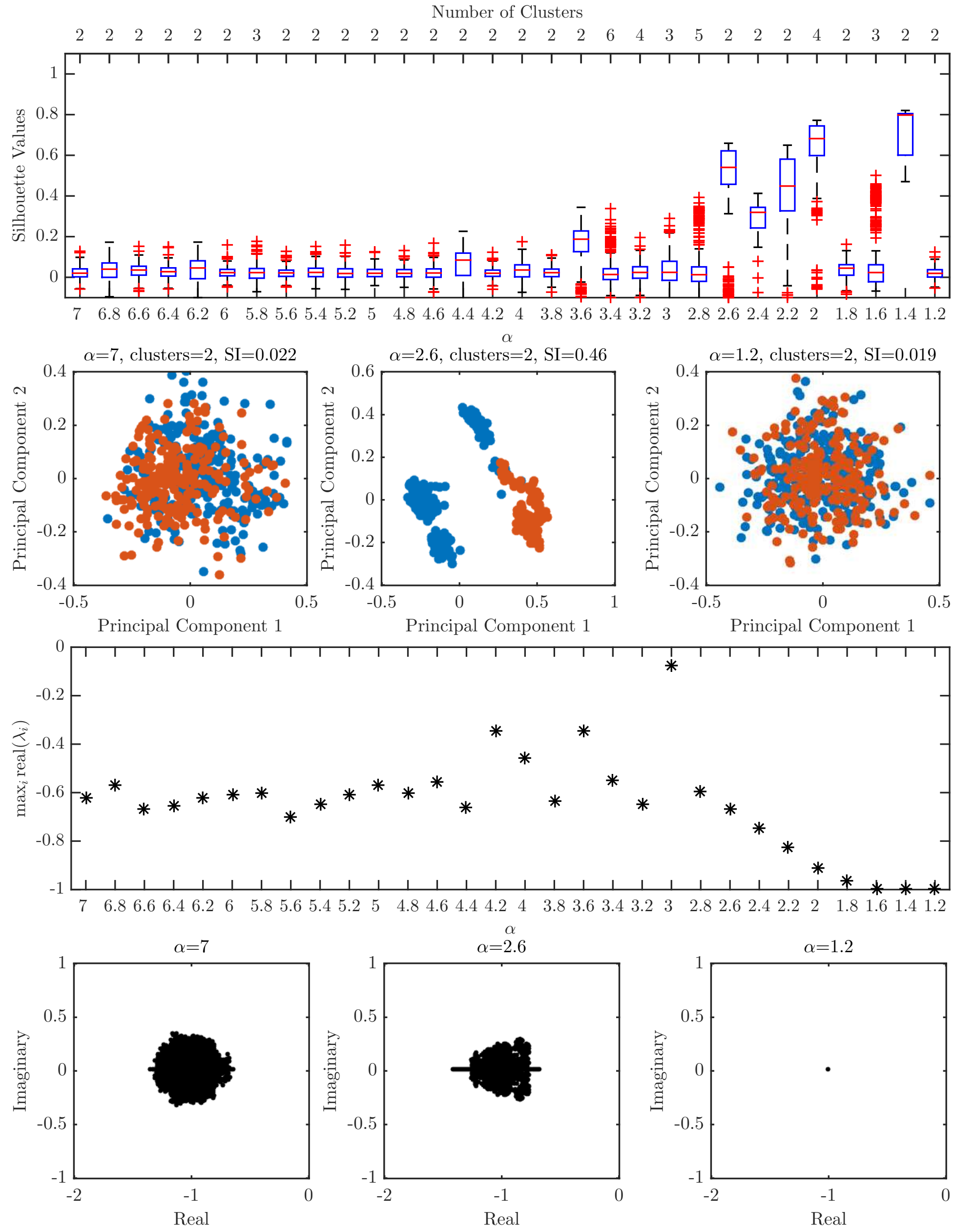

Supplementary Text Figure T11: Universal Model Heterogeneity Study Scenario 6 in Table T1. The first plot is a comprehensive clustering analysis of the steady state values obtained from the Lotka-Volterra simulations. The x-axis denotes the heterogeneity value α. The box plots are the silhouette values pertaining to the number of clusters for which the silhouette index (maximum over the number of clusters of the mean silhouette value for each given total number of cluster) was defined. The total number of clusters pertaining to the silhouette index is denoted on the top x-axis. The second row is a principle coordinate analysis of the steady state values obtained at three different heterogeneity values. Clusters are color coded to match the optimal clustering from k-medoids. The third row of the figure plots $\max_{i,j} \text{real}(\lambda_i(A^{[j]}))$ as a function of α. The fourth row is a plot of $\lambda_i(A^{[j]})$for $i \in \{1, 2, \ldots, 80\}$ and $j \in \{1, 2, \ldots, 500\}$ at three values of α.

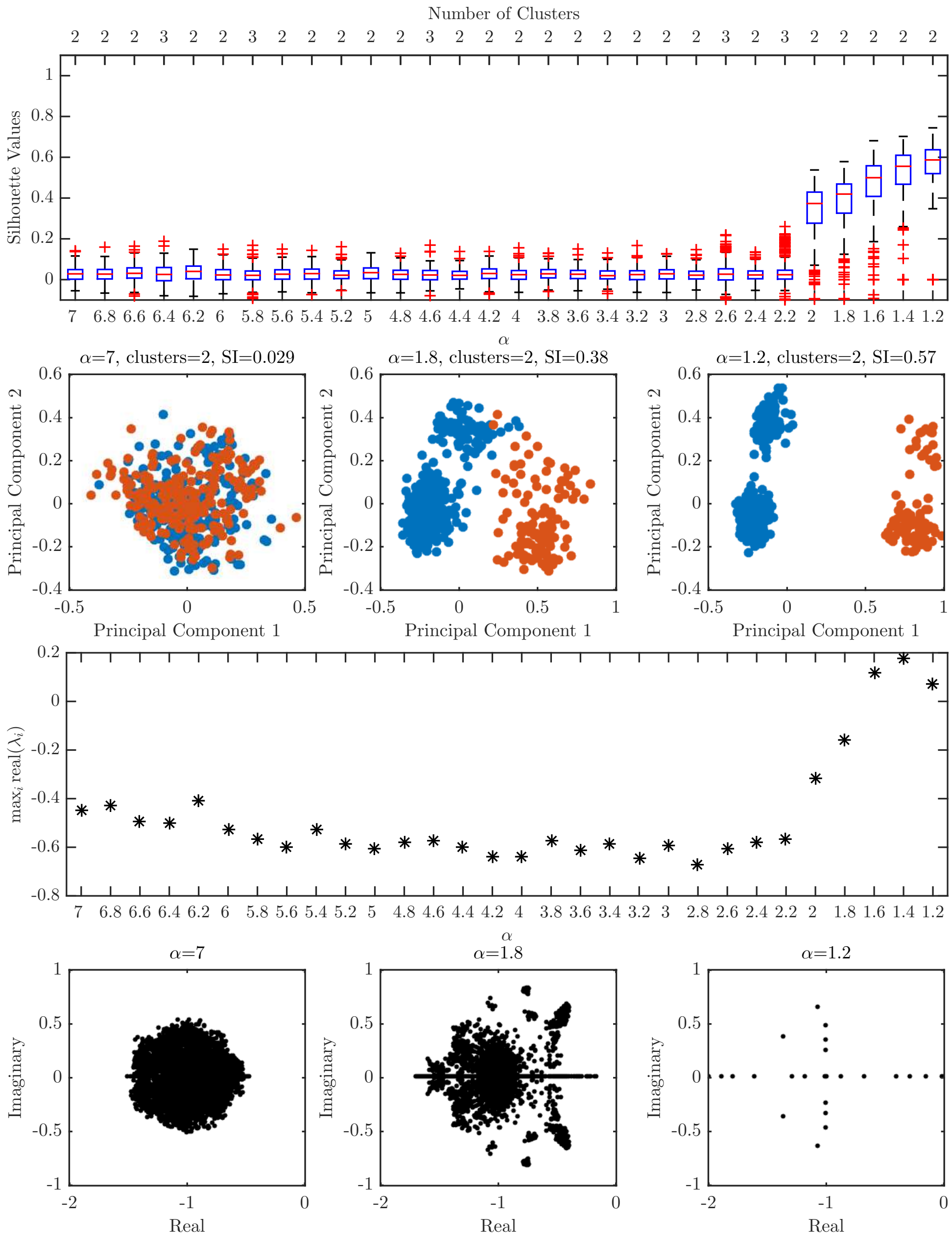

Supplementary Text Figure T12: Universal Model Heterogeneity Study Scenario 7 in Table T1. The first plot is a comprehensive clustering analysis of the steady state values obtained from the Lotka-Volterra simulations. The x-axis denotes the heterogeneity value α. The box plots are the silhouette values pertaining to the number of clusters for which the silhouette index (maximum over the number of clusters of the mean silhouette value for each given total number of cluster) was defined. The total number of clusters pertaining to the silhouette index is denoted on the top x-axis. The second row is a principle coordinate analysis of the steady state values obtained at three different heterogeneity values. Clusters are color coded to match the optimal clustering from k-medoids. The third row of the figure plots $\max_{i,j}$ real$(\lambda_i(A^{[j]}))$ as a function of α. The fourth row is a plot of $\lambda_i(A^{[j]})$for $i \in \{1, 2, \ldots, 80\}$ and $j \in \{1, 2, \ldots, 500\}$ at three values of α.

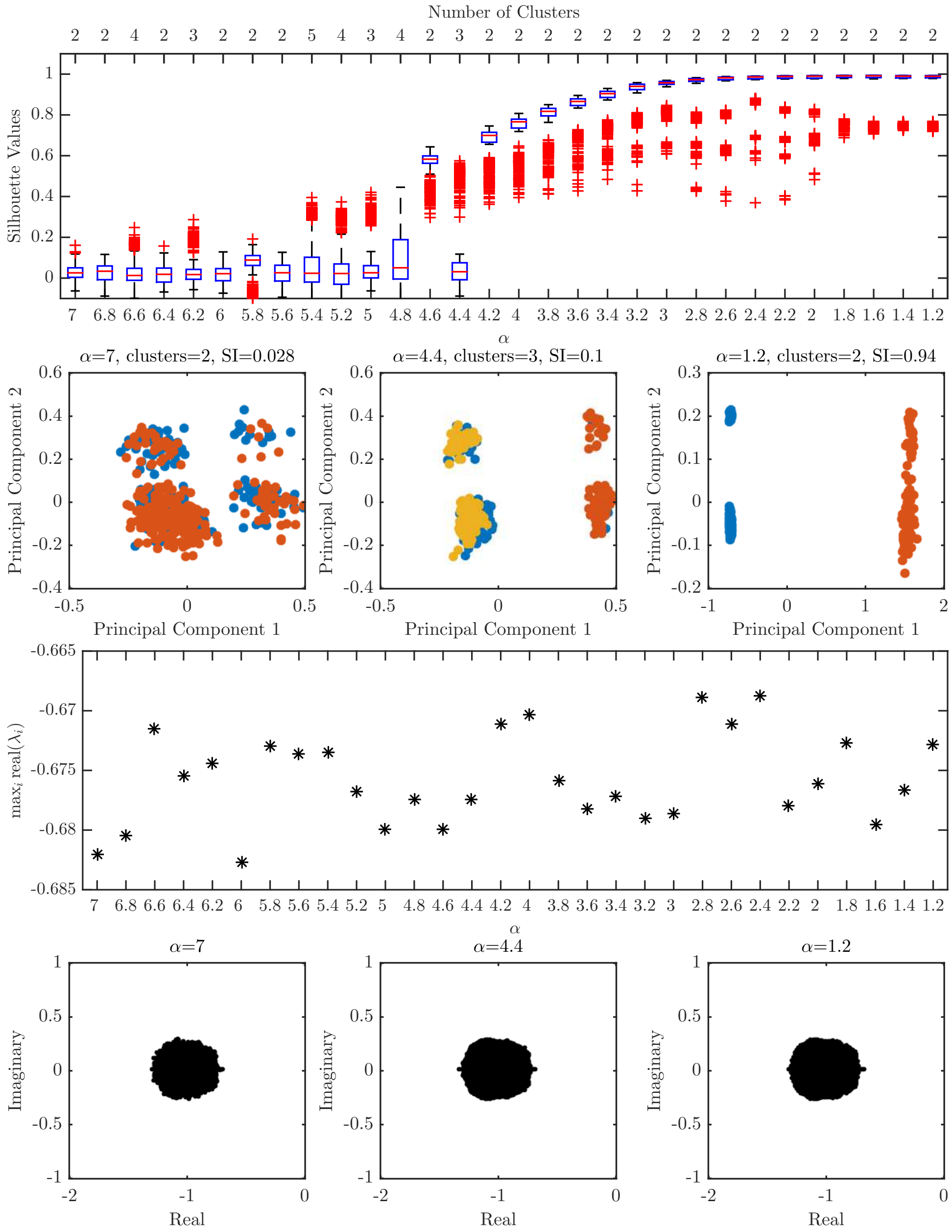

Supplementary Text Figure T13: Universal Model Heterogeneity Study Scenario 8 in Table T1. The first plot is a comprehensive clustering analysis of the steady state values obtained from the Lotka-Volterra simulations. The x-axis denotes the heterogeneity value α. The box plots are the silhouette values pertaining to the number of clusters for which the silhouette index (maximum over the number of clusters of the mean silhouette value for each given total number of cluster) was defined. The total number of clusters pertaining to the silhouette index is denoted on the top x-axis. The second row is a principle coordinate analysis of the steady state values obtained at three different heterogeneity values. Clusters are color coded to match the optimal clustering from k-medoids. The third row of the figure plots $\max_{i,j} \text{real}(\lambda_i(A^{[j]}))$ as a function of α. The fourth row is a plot of $\lambda_i(A^{[j]})$for $i \in \{1, 2, \ldots, 80\}$ and $j \in \{1, 2, \ldots, 500\}$ at three values of α.

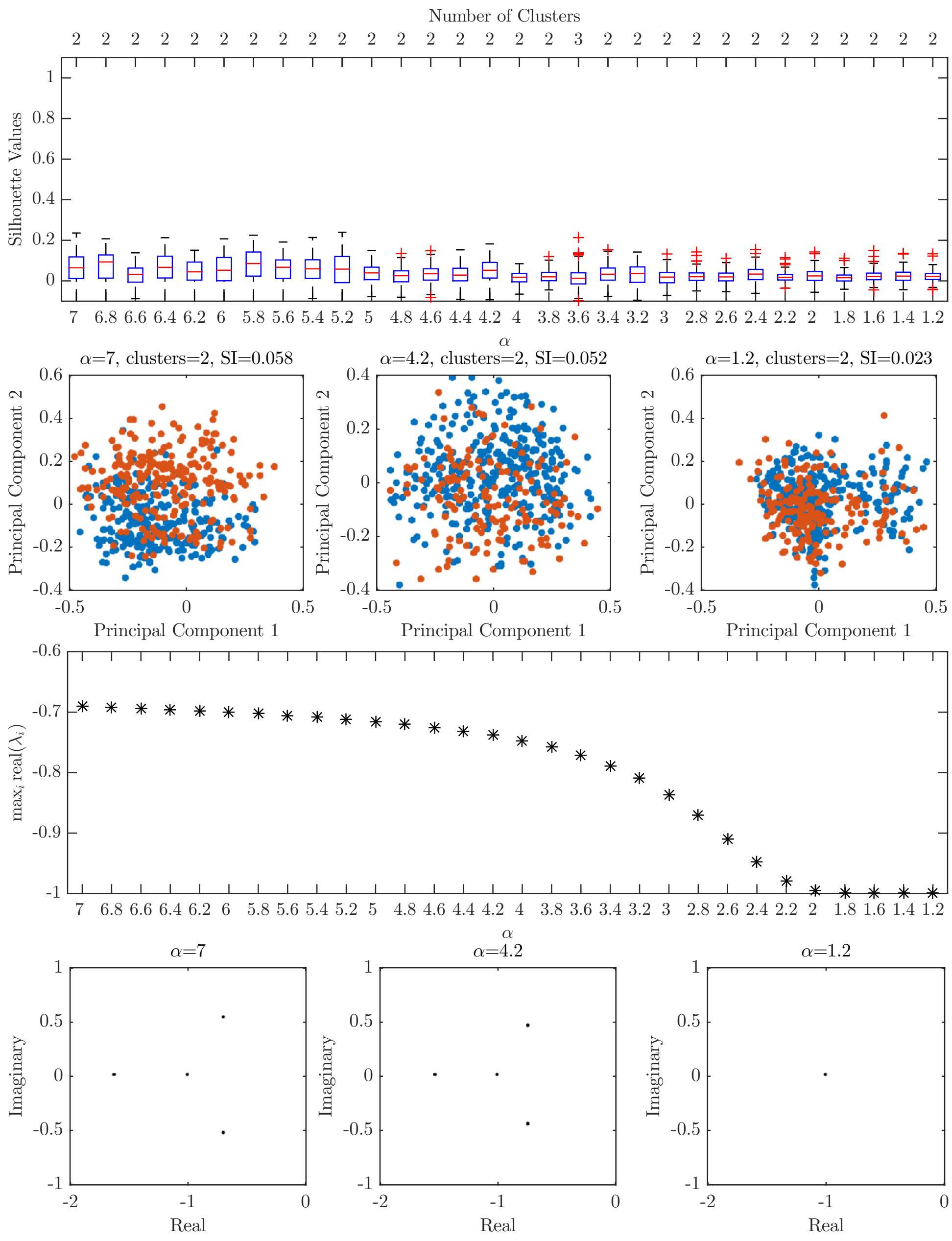

Supplementary Text Figure T14: Universal Model Sparsity Study Scenario 1 in Table T2. The first plot is a comprehensive clustering analysis of the steady state values obtained from the Lotka-Volterra simulations. The x-axis denotes the heterogeneity value α. The box plots are the silhouette values pertaining to the number of clusters for which the silhouette index (maximum over the number of clusters of the mean silhouette value for each given total number of cluster) was defined. The total number of clusters pertaining to the silhouette index is denoted on the top x-axis. The second row is a principle coordinate analysis of the steady state values obtained at three different heterogeneity values. Clusters are color coded to match the optimal clustering from k-medoids. The third row of the figure plots $\max_{i,j} \text{real}(\lambda_i(A^{[j]}))$ as a function of α. The fourth row is a plot of $\lambda_i(A^{[j]})$ for $i \in \{1, 2, \ldots, 80\}$ and $j \in \{1, 2, \ldots, 500\}$ at three values of α.

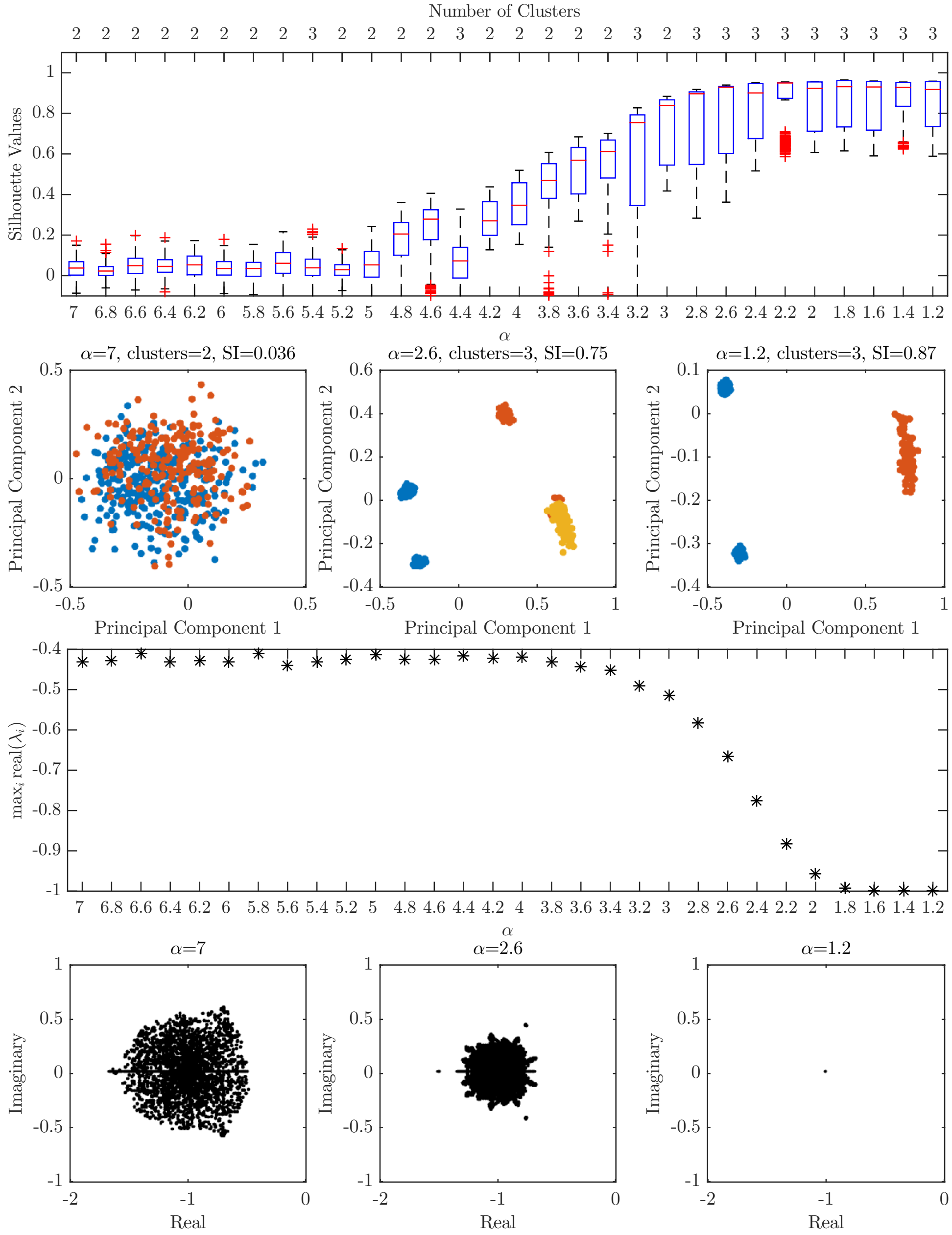

Supplementary Text Figure T15: Universal Model Sparsity Study Scenario 2 in Table T2. The first plot is a comprehensive clustering analysis of the steady state values obtained from the Lotka-Volterra simulations. The x-axis denotes the heterogeneity value α. The box plots are the silhouette values pertaining to the number of clusters for which the silhouette index (maximum over the number of clusters of the mean silhouette value for each given total number of cluster) was defined. The total number of clusters pertaining to the silhouette index is denoted on the top x-axis. The second row is a principle coordinate analysis of the steady state values obtained at three different heterogeneity values. Clusters are color coded to match the optimal clustering from k-medoids. The third row of the figure plots $\max_{i,j} \text{real}(\lambda_i(A^{[j]}))$ as a function of α. The fourth row is a plot of $\lambda_i(A^{[j]})$ for $i \in \{1, 2, \ldots, 80\}$ and $j \in \{1, 2, \ldots, 500\}$ at three values of α.

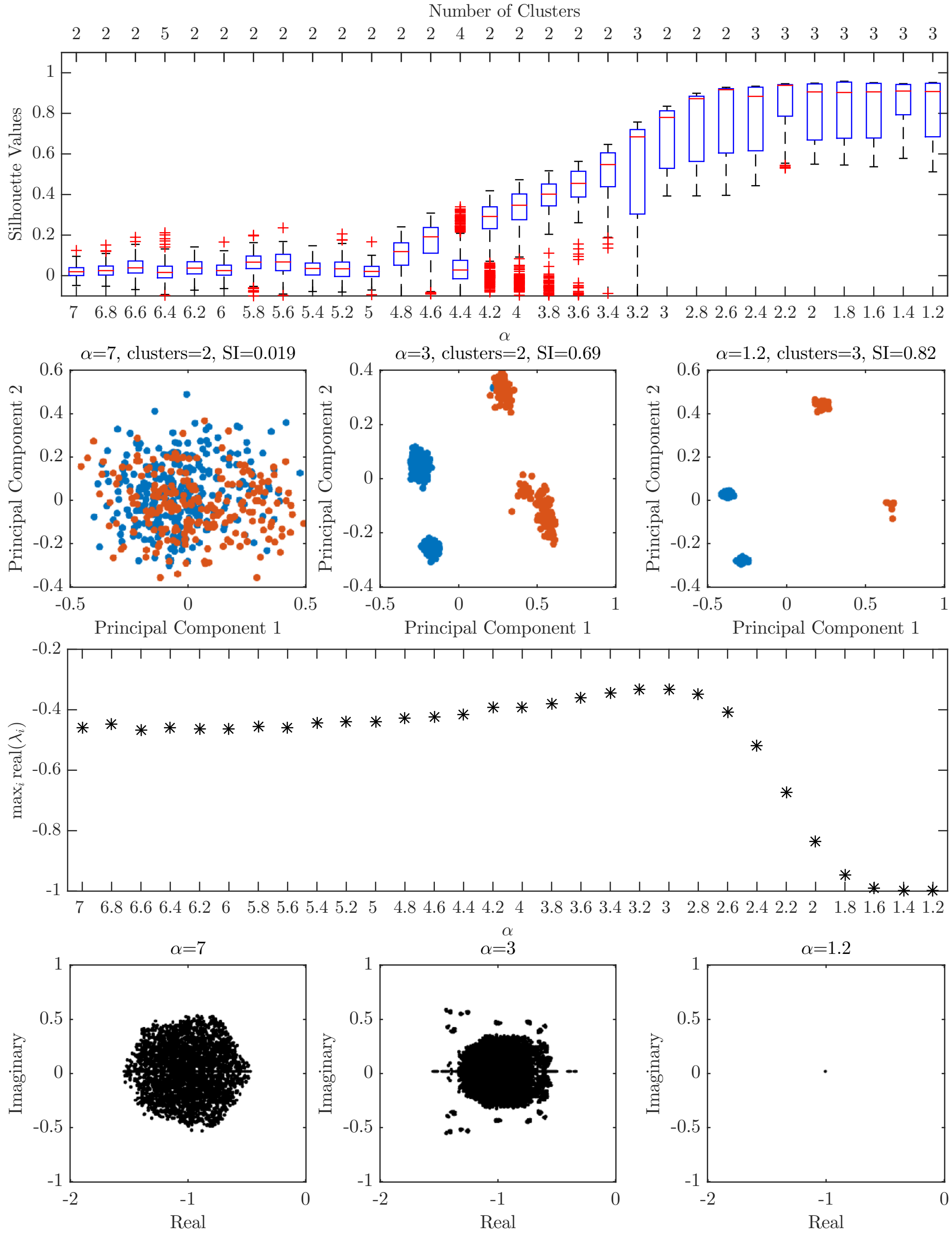

Supplementary Text Figure T16: Universal Model Sparsity Study Scenario 3 in Table T2. The first plot is a comprehensive clustering analysis of the steady state values obtained from the Lotka-Volterra simulations. The x-axis denotes the heterogeneity value α. The box plots are the silhouette values pertaining to the number of clusters for which the silhouette index (maximum over the number of clusters of the mean silhouette value for each given total number of cluster) was defined. The total number of clusters pertaining to the silhouette index is denoted on the top x-axis. The second row is a principle coordinate analysis of the steady state values obtained at three different heterogeneity values. Clusters are color coded to match the optimal clustering from k-medoids. The third row of the figure plots $\max_{i,j} \mathrm{real}(\lambda_i(A^{[j]}))$ as a function of α. The fourth row is a plot of $\lambda_i(A^{[j]})$ for $i \in \{1, 2, \ldots, 80\}$ and $j \in \{1, 2, \ldots, 500\}$ at three values of α.

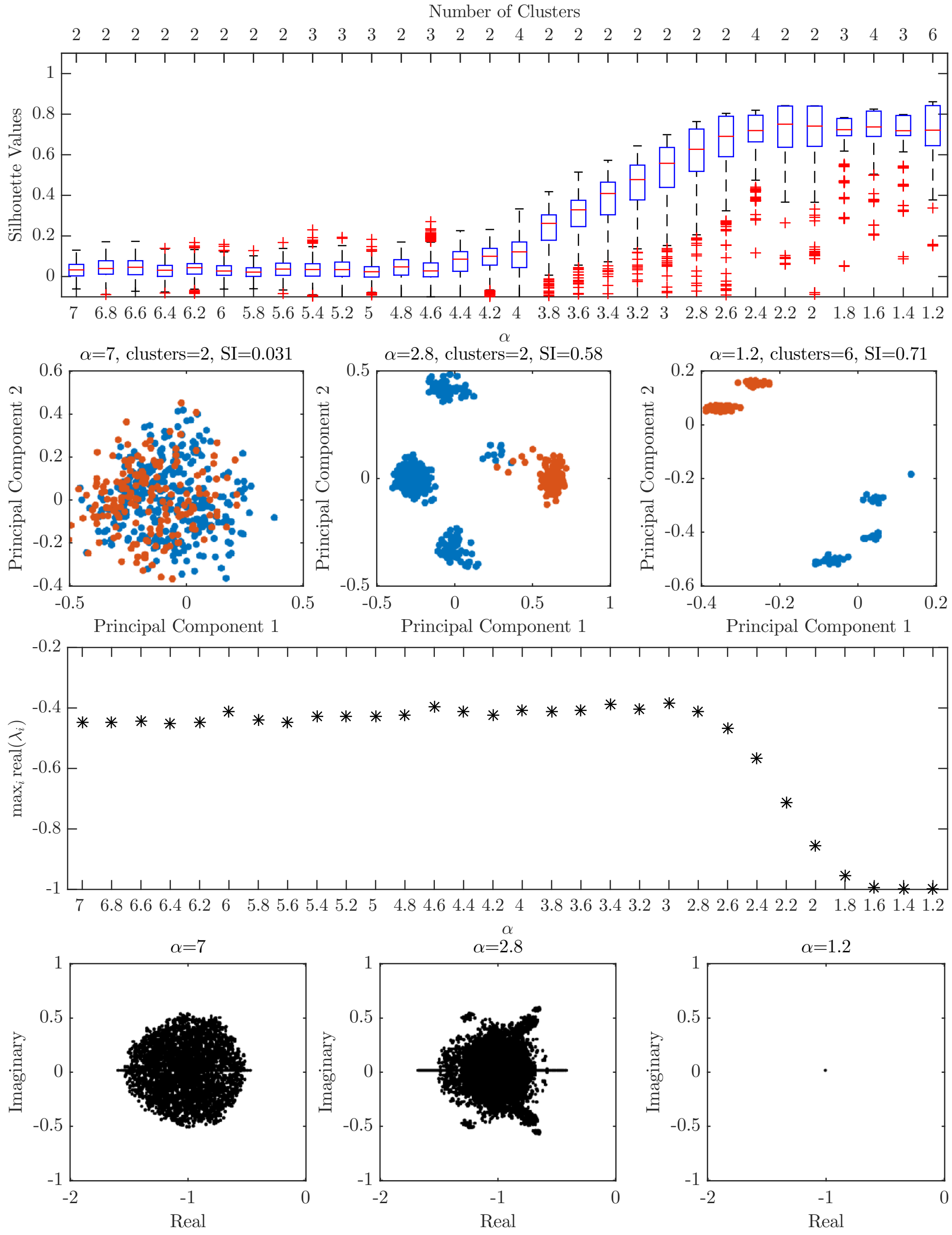

Supplementary Text Figure T17: Universal Model Sparsity Study Scenario 4 in Table T2. The first plot is a comprehensive clustering analysis of the steady state values obtained from the Lotka-Volterra simulations. The x-axis denotes the heterogeneity value α. The box plots are the silhouette values pertaining to the number of clusters for which the silhouette index (maximum over the number of clusters of the mean silhouette value for each given total number of cluster) was defined. The total number of clusters pertaining to the silhouette index is denoted on the top x-axis. The second row is a principle coordinate analysis of the steady state values obtained at three different heterogeneity values. Clusters are color coded to match the optimal clustering from k-medoids. The third row of the figure plots $\max_{i,j} \text{real}(\lambda_i(A^{[j]}))$ as a function of α. The fourth row is a plot of $\lambda_i(A^{[j]})$ for $i \in \{1, 2, \ldots, 80\}$ and $j \in \{1, 2, \ldots, 500\}$ at three values of α.

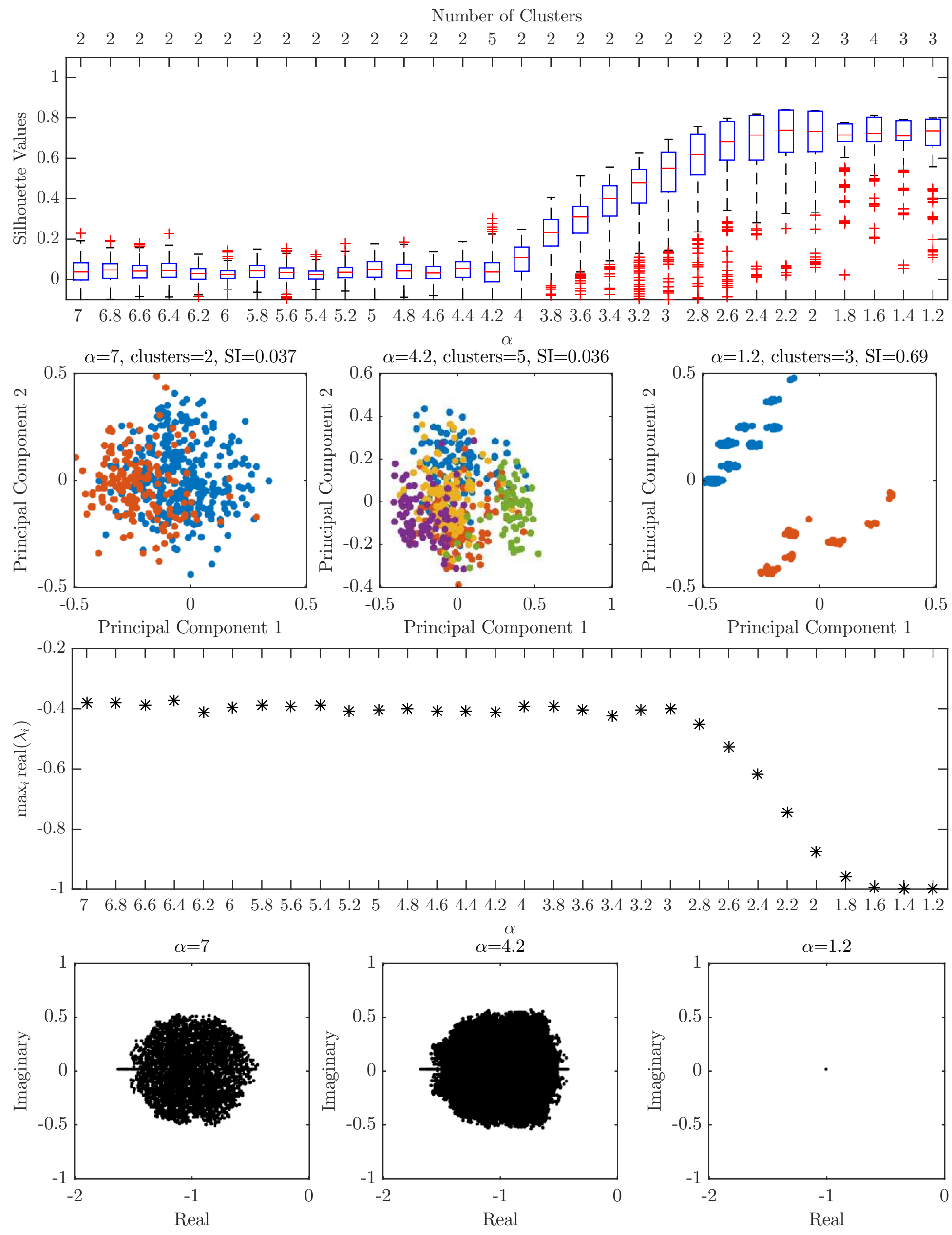

Supplementary Text Figure T18: Universal Model Sparsity Study Scenario 5 in Table T2. The first plot is a comprehensive clustering analysis of the steady state values obtained from the Lotka-Volterra simulations. The x-axis denotes the heterogeneity value α. The box plots are the silhouette values pertaining to the number of clusters for which the silhouette index (maximum over the number of clusters of the mean silhouette value for each given total number of cluster) was defined. The total number of clusters pertaining to the silhouette index is denoted on the top x-axis. The second row is a principle coordinate analysis of the steady state values obtained at three different heterogeneity values. Clusters are color coded to match the optimal clustering from k-medoids. The third row of the figure plots $\max_{i,j} \text{real}(\lambda_i(A^{[j]}))$ as a function of α. The fourth row is a plot of $\lambda_i(A^{[j]})$ for $i \in \{1, 2, \ldots, 80\}$ and $j \in \{1, 2, \ldots, 500\}$ at three values of α.

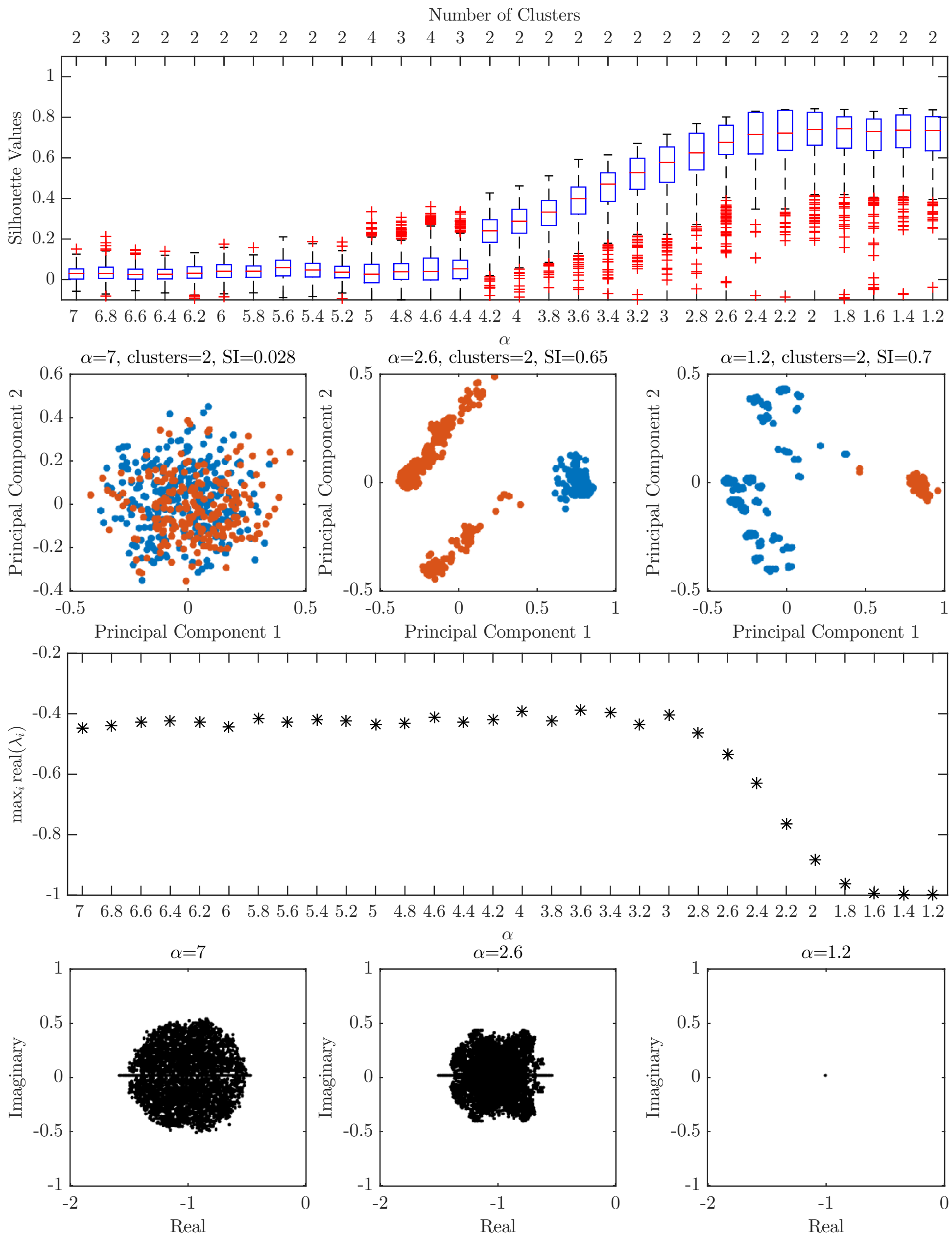

Supplementary Text Figure T19: Universal Model Sparsity Study Scenario 6 in Table T2. The first plot is a comprehensive clustering analysis of the steady state values obtained from the Lotka-Volterra simulations. The x-axis denotes the heterogeneity value α. The box plots are the silhouette values pertaining to the number of clusters for which the silhouette index (maximum over the number of clusters of the mean silhouette value for each given total number of cluster) was defined. The total number of clusters pertaining to the silhouette index is denoted on the top x-axis. The second row is a principle coordinate analysis of the steady state values obtained at three different heterogeneity values. Clusters are color coded to match the optimal clustering from k-medoids. The third row of the figure plots $\max_{i,j} \text{real}(\lambda_i(A^{[j]}))$ as a function of α. The fourth row is a plot of $\lambda_i(A^{[j]})$ for $i \in \{1, 2, \ldots, 80\}$ and $j \in \{1, 2, \ldots, 500\}$ at three values of α.

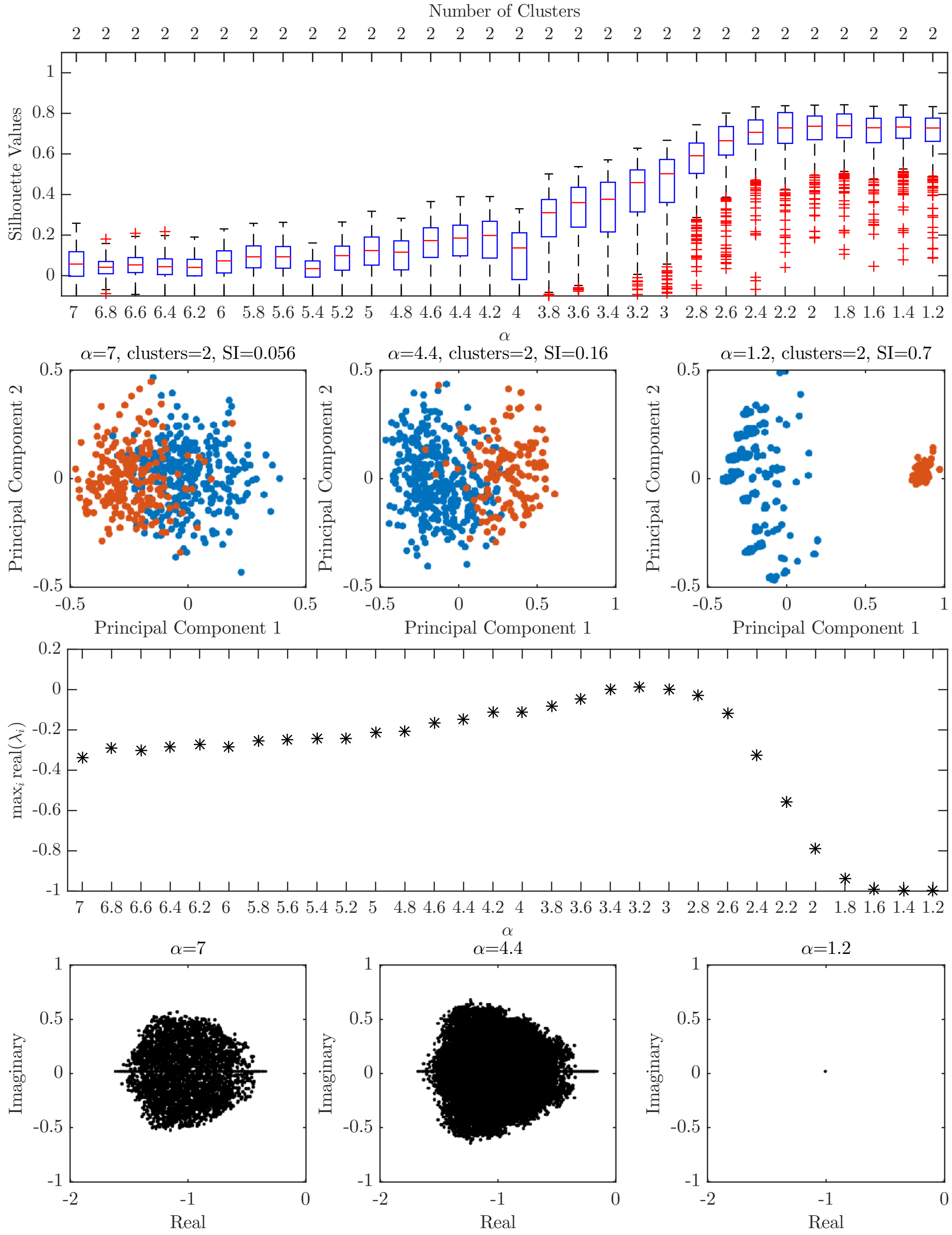

Supplementary Text Figure T20: Universal Model Sparsity Study Scenario 7 in Table T2. The first plot is a comprehensive clustering analysis of the steady state values obtained from the Lotka-Volterra simulations. The x-axis denotes the heterogeneity value α. The box plots are the silhouette values pertaining to the number of clusters for which the silhouette index (maximum over the number of clusters of the mean silhouette value for each given total number of cluster) was defined. The total number of clusters pertaining to the silhouette index is denoted on the top x-axis. The second row is a principle coordinate analysis of the steady state values obtained at three different heterogeneity values. Clusters are color coded to match the optimal clustering from k-medoids. The third row of the figure plots $\max_{i,j} \text{real}(\lambda_i(A^{[j]}))$ as a function of α. The fourth row is a plot of $\lambda_i(A^{[j]})$ for $i \in \{1, 2, \ldots, 80\}$ and $j \in \{1, 2, \ldots, 500\}$ at three values of α.

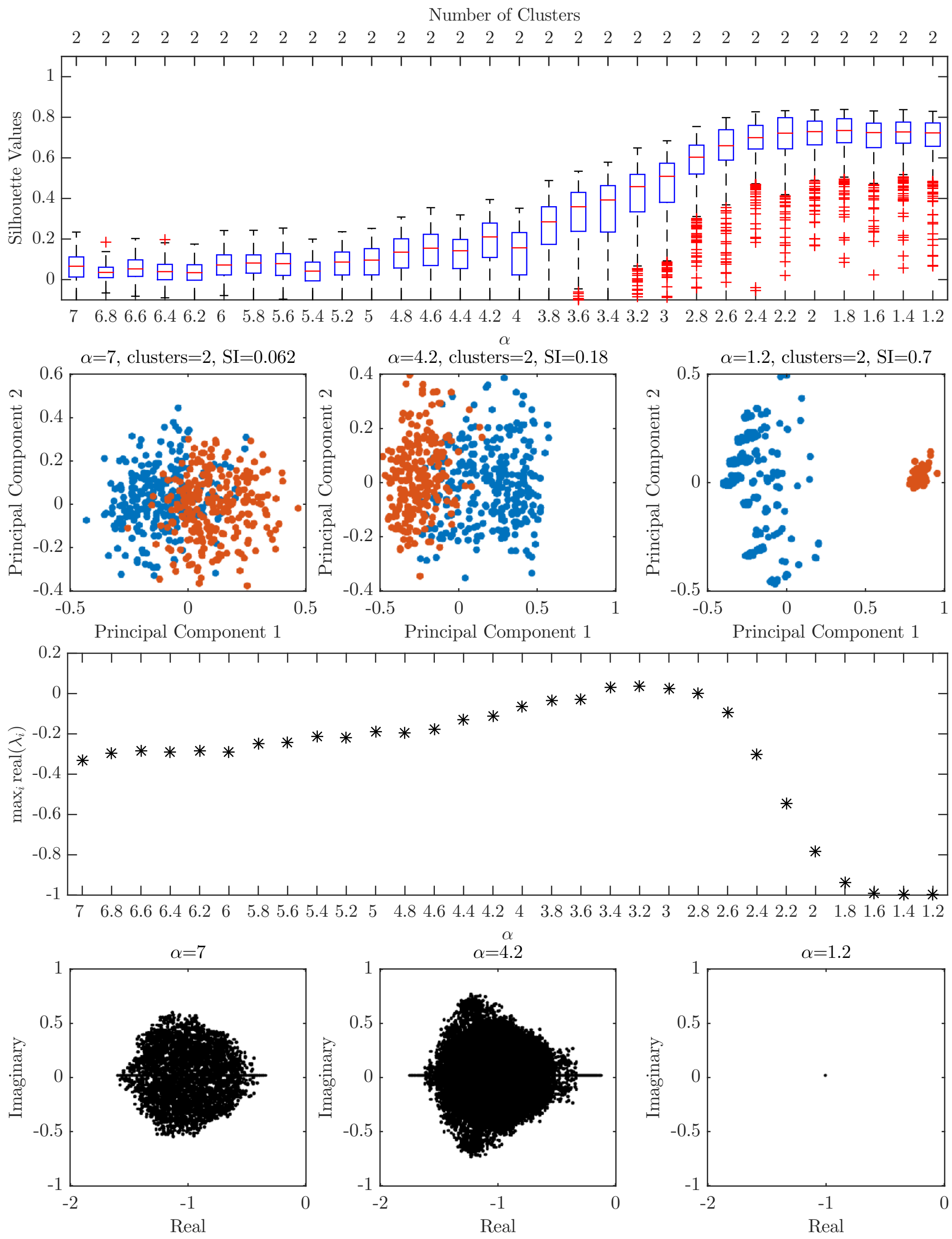

Supplementary Text Figure T21: Universal Model Sparsity Study Scenario 8 in Table T2. The first plot is a comprehensive clustering analysis of the steady state values obtained from the Lotka-Volterra simulations. The x-axis denotes the heterogeneity value α. The box plots are the silhouette values pertaining to the number of clusters for which the silhouette index (maximum over the number of clusters of the mean silhouette value for each given total number of cluster) was defined. The total number of clusters pertaining to the silhouette index is denoted on the top x-axis. The second row is a principle coordinate analysis of the steady state values obtained at three different heterogeneity values. Clusters are color coded to match the optimal clustering from k-medoids. The third row of the figure plots $\max_{i,j} \text{real}(\lambda_i(A^{[j]}))$ as a function of α. The fourth row is a plot of $\lambda_i(A^{[j]})$ for $i \in \{1, 2, \ldots, 80\}$ and $j \in \{1, 2, \ldots, 500\}$ at three values of α.

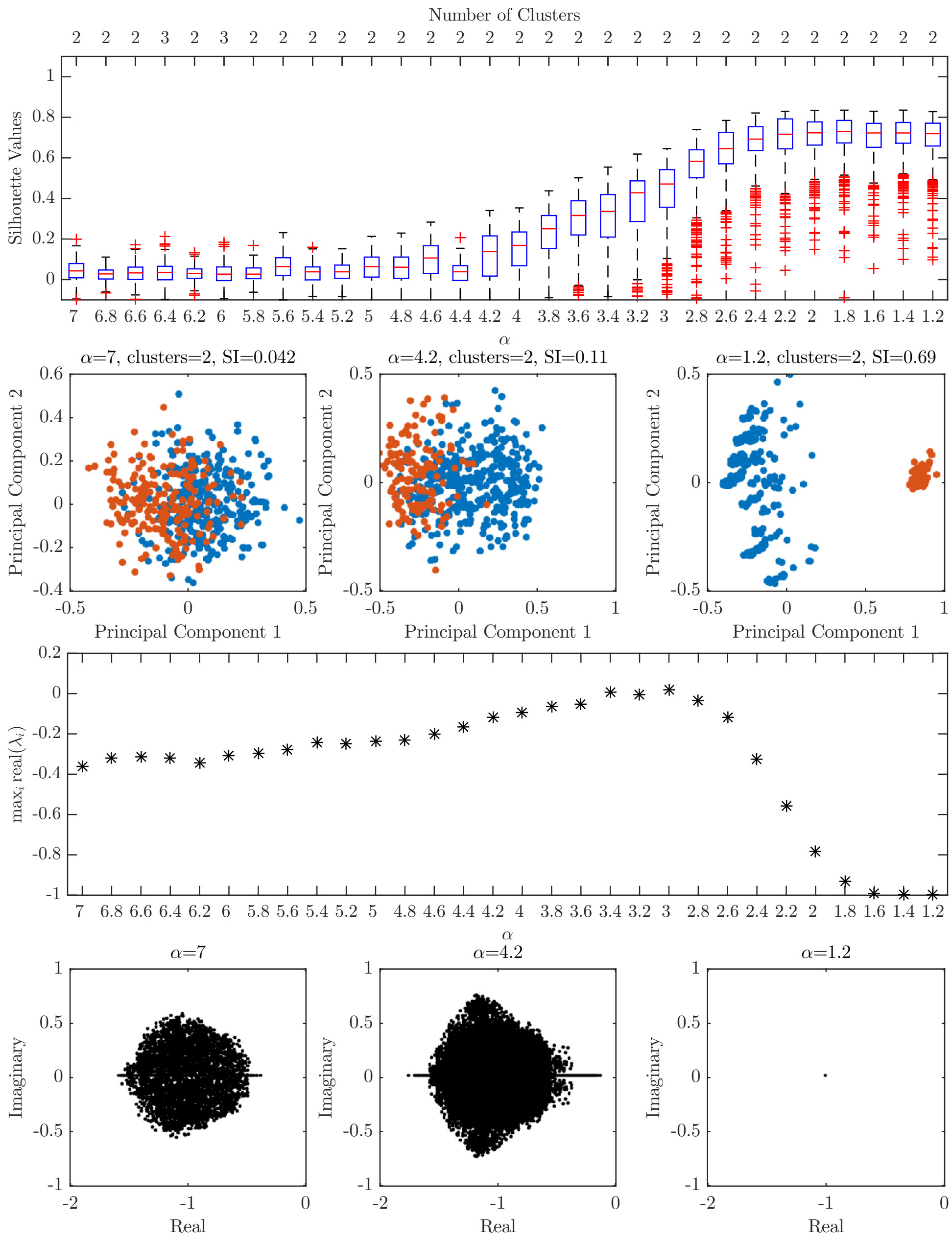

Supplementary Text Figure T22: Universal Model Sparsity Study Scenario 9 in Table T2. The first plot is a comprehensive clustering analysis of the steady state values obtained from the Lotka-Volterra simulations. The x-axis denotes the heterogeneity value α. The box plots are the silhouette values pertaining to the number of clusters for which the silhouette index (maximum over the number of clusters of the mean silhouette value for each given total number of cluster) was defined. The total number of clusters pertaining to the silhouette index is denoted on the top x-axis. The second row is a principle coordinate analysis of the steady state values obtained at three different heterogeneity values. Clusters are color coded to match the optimal clustering from k-medoids. The third row of the figure plots $\max_{i,j} \text{real}(\lambda_i(A^{[j]}))$ as a function of α. The fourth row is a plot of $\lambda_i(A^{[j]})$ for $i \in \{1, 2, \ldots, 80\}$ and $j \in \{1, 2, \ldots, 500\}$ at three values of α.

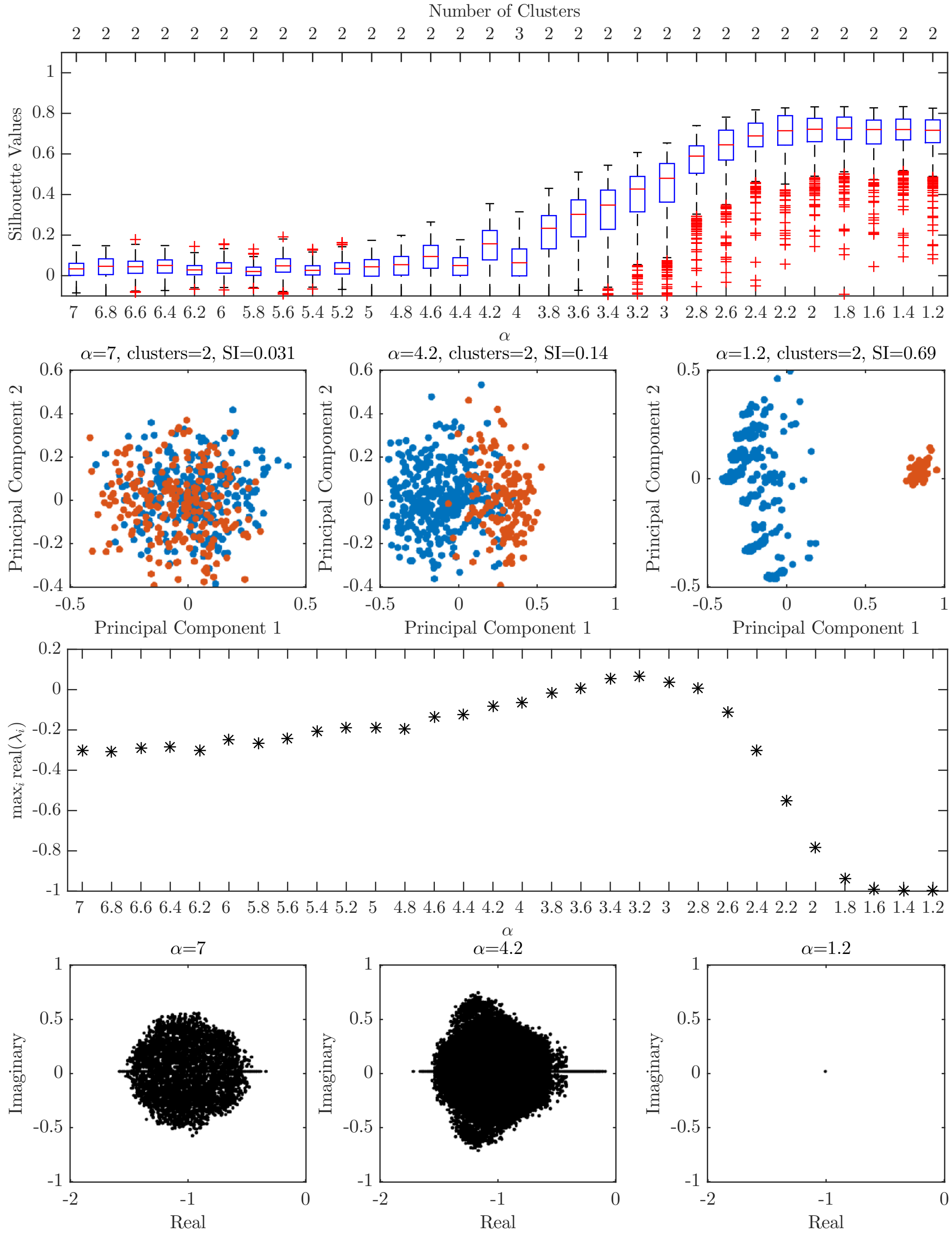

Supplementary Text Figure T23: Universal Model Sparsity Study Scenario 10 in Table T2. The first plot is a comprehensive clustering analysis of the steady state values obtained from the Lotka-Volterra simulations. The x-axis denotes the heterogeneity value α. The box plots are the silhouette values pertaining to the number of clusters for which the silhouette index (maximum over the number of clusters of the mean silhouette value for each given total number of cluster) was defined. The total number of clusters pertaining to the silhouette index is denoted on the top x-axis. The second row is a principle coordinate analysis of the steady state values obtained at three different heterogeneity values. Clusters are color coded to match the optimal clustering from k-medoids. The third row of the figure plots $\max_{i,j} \text{real}(\lambda_i(A^{[j]}))$ as a function of α. The fourth row is a plot of $\lambda_i(A^{[j]})$ for $i \in \{1, 2, \ldots, 80\}$ and $j \in \{1, 2, \ldots, 500\}$ at three values of α.

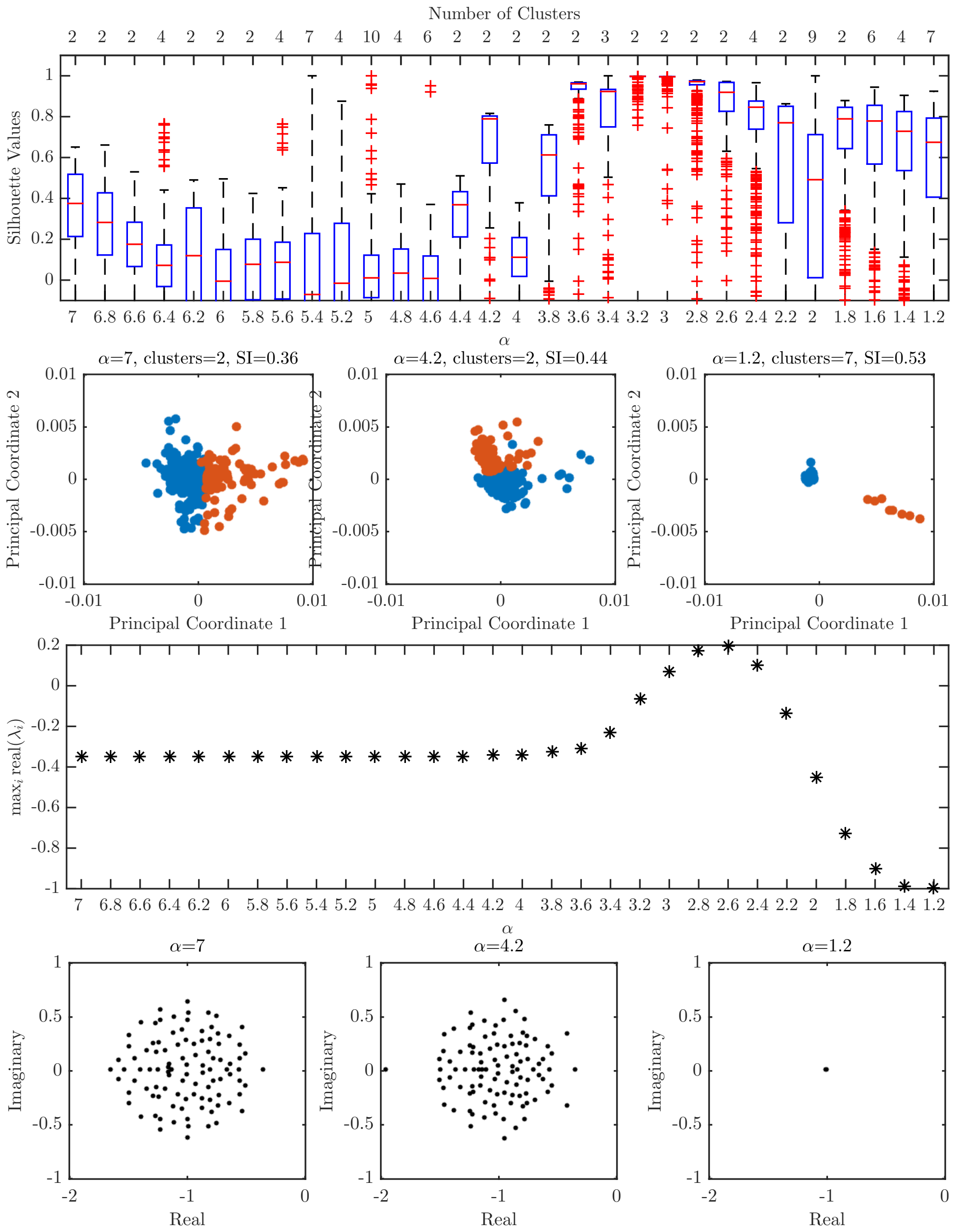

Supplementary Text Figure T24: Universal Model Community Size Overlap Study Scenario 1 in Table T3. The first plot is a comprehensive clustering analysis of the steady state values obtained from the Lotka-Volterra simulations. The x-axis denotes the heterogeneity value α. The box plots are the silhouette values pertaining to the number of clusters for which the silhouette index (maximum over the number of clusters of the mean silhouette value for each given total number of cluster) was defined. The total number of clusters pertaining to the silhouette index is denoted on the top x-axis. The second row is a principle coordinate analysis of the steady state values obtained at three different heterogeneity values. Clusters are color coded to match the optimal clustering from k-medoids. The third row of the figure plots $\max_{i,j} \text{real}(\lambda_i(A^{[j]}))$ as a function of α. The fourth row is a plot of $\lambda_i(A^{[j]})$ for $i \in \{1, 2, \ldots, p\}$ and $j \in \{1, 2, \ldots, 500\}$ at three values of α.

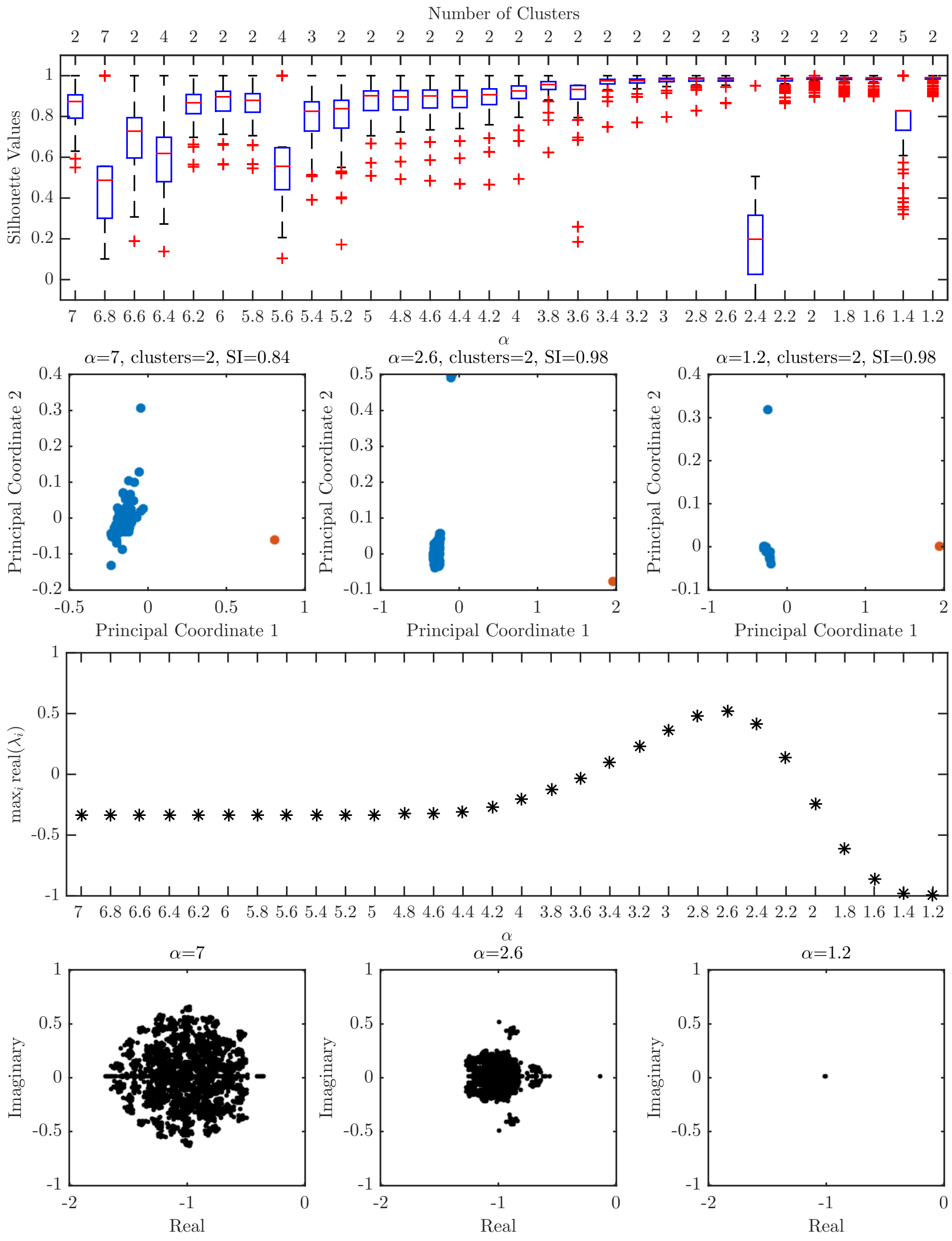

Supplementary Text Figure T25: Universal Model Community Size Overlap Study Scenario 2 in Table T3. The first plot is a comprehensive clustering analysis of the steady state values obtained from the Lotka-Volterra simulations. The x-axis denotes the heterogeneity value α. The box plots are the silhouette values pertaining to the number of clusters for which the silhouette index (maximum over the number of clusters of the mean silhouette value for each given total number of cluster) was defined. The total number of clusters pertaining to the silhouette index is denoted on the top x-axis. The second row is a principle coordinate analysis of the steady state values obtained at three different heterogeneity values. Clusters are color coded to match the optimal clustering from k-medoids. The third row of the figure plots $\max_{i,j} \text{real}(\lambda_i(A^{[j]}))$ as a function of α. The fourth row is a plot of $\lambda_i(A^{[j]})$ for $i \in \{1, 2, \ldots, p\}$ and $j \in \{1, 2, \ldots, 500\}$ at three values of α.

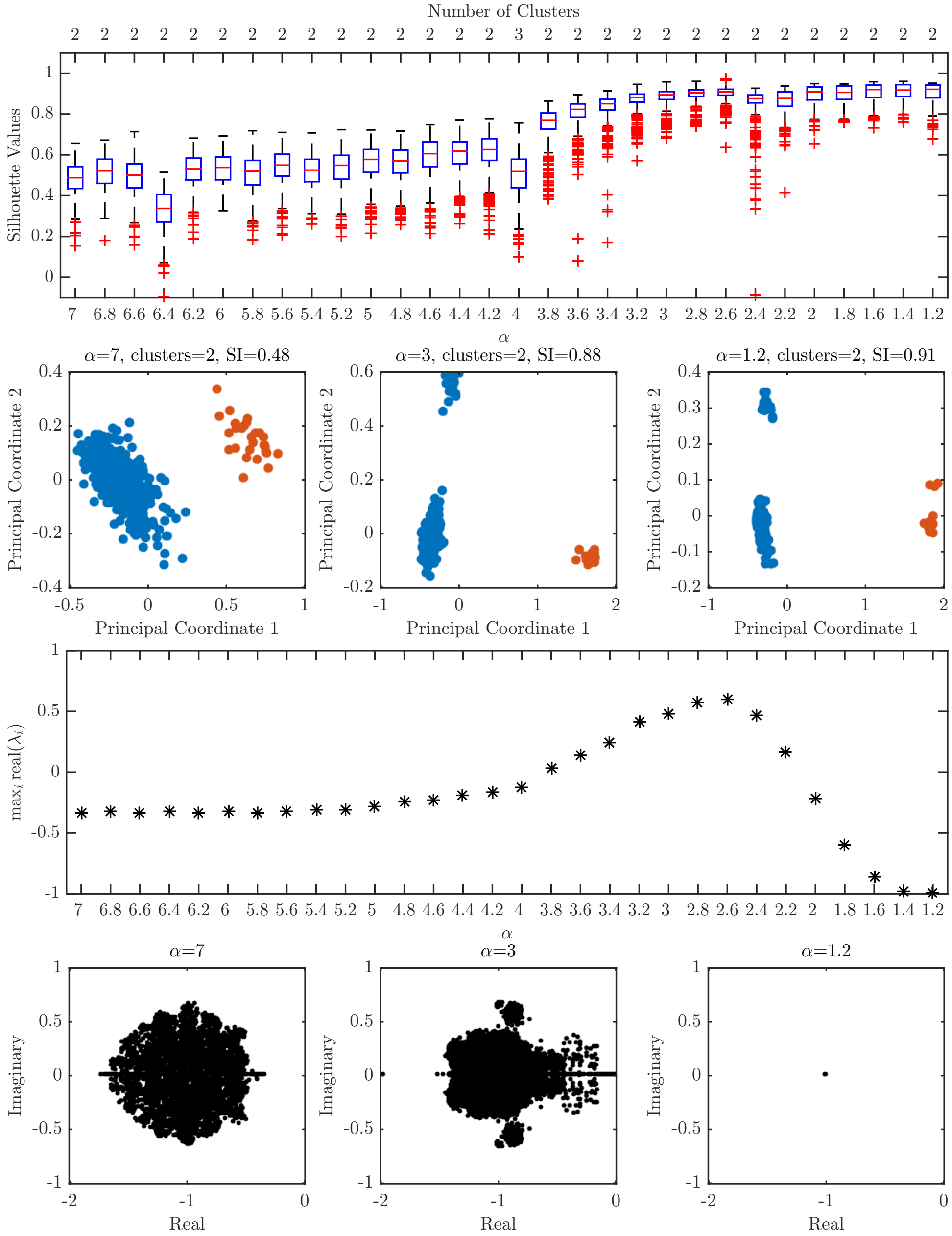

Supplementary Text Figure T26: Universal Model Community Size Overlap Study Scenario 3 in Table T3. The first plot is a comprehensive clustering analysis of the steady state values obtained from the Lotka-Volterra simulations. The x-axis denotes the heterogeneity value α. The box plots are the silhouette values pertaining to the number of clusters for which the silhouette index (maximum over the number of clusters of the mean silhouette value for each given total number of cluster) was defined. The total number of clusters pertaining to the silhouette index is denoted on the top x-axis. The second row is a principle coordinate analysis of the steady state values obtained at three different heterogeneity values. Clusters are color coded to match the optimal clustering from k-medoids. The third row of the figure plots $\max_{i,j}$ real$(\lambda_i(A^{[j]}))$ as a function of α. The fourth row is a plot of $\lambda_i(A^{[j]})$ for $i \in \{1, 2, \ldots, p\}$ and $j \in \{1, 2, \ldots, 500\}$ at three values of α.

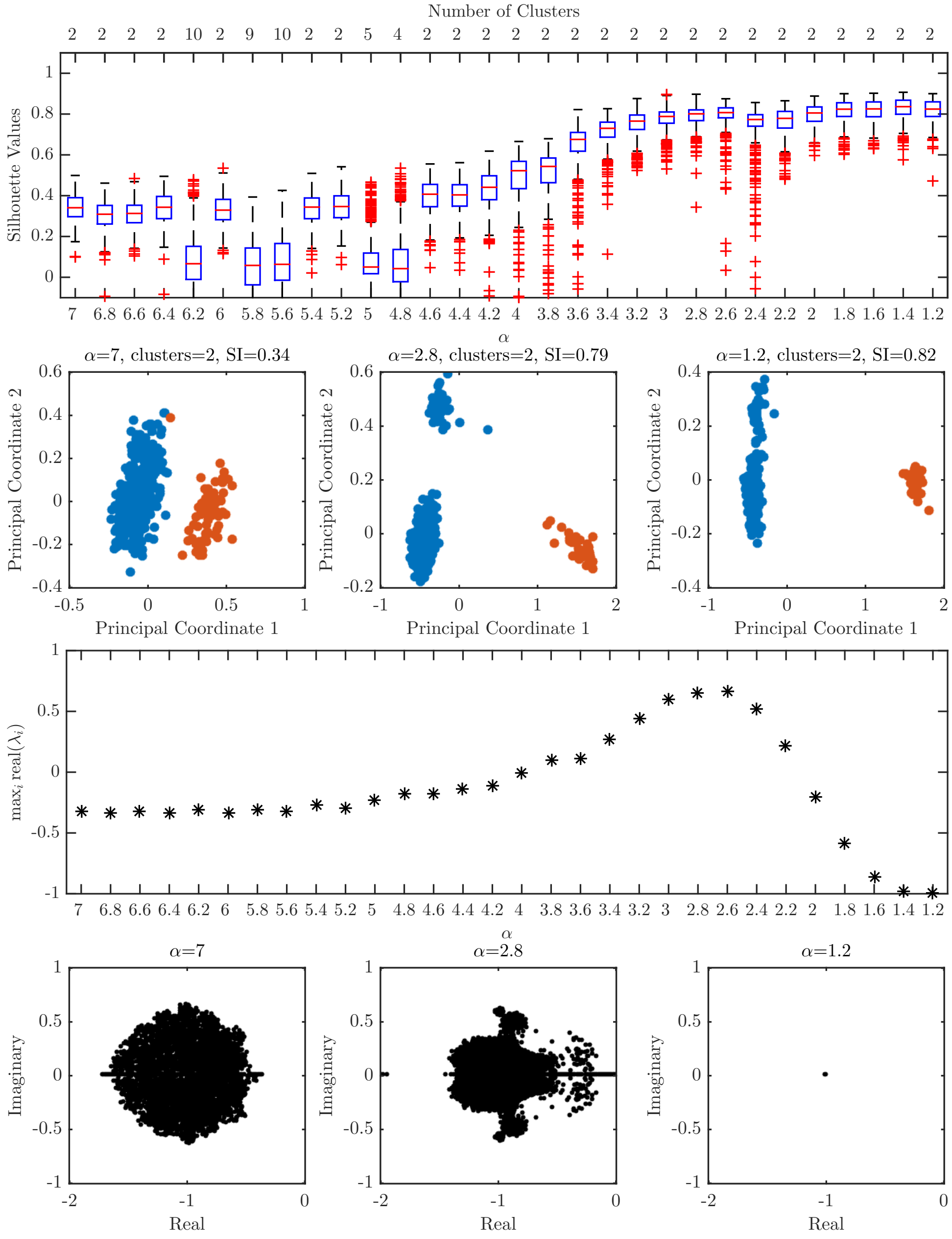

Supplementary Text Figure T27: Universal Model Community Size Overlap Study Scenario 4 in Table T3. The first plot is a comprehensive clustering analysis of the steady state values obtained from the Lotka-Volterra simulations. The x-axis denotes the heterogeneity value α. The box plots are the silhouette values pertaining to the number of clusters for which the silhouette index (maximum over the number of clusters of the mean silhouette value for each given total number of cluster) was defined. The total number of clusters pertaining to the silhouette index is denoted on the top x-axis. The second row is a principle coordinate analysis of the steady state values obtained at three different heterogeneity values. Clusters are color coded to match the optimal clustering from k-medoids. The third row of the figure plots $\max_{i,j}\, \mathrm{real}(\lambda_i(A^{[j]}))$ as a function of α. The fourth row is a plot of $\lambda_i(A^{[j]})$ for $i \in \{1, 2, \ldots, p\}$ and $j \in \{1, 2, \ldots, 500\}$ at three values of α.

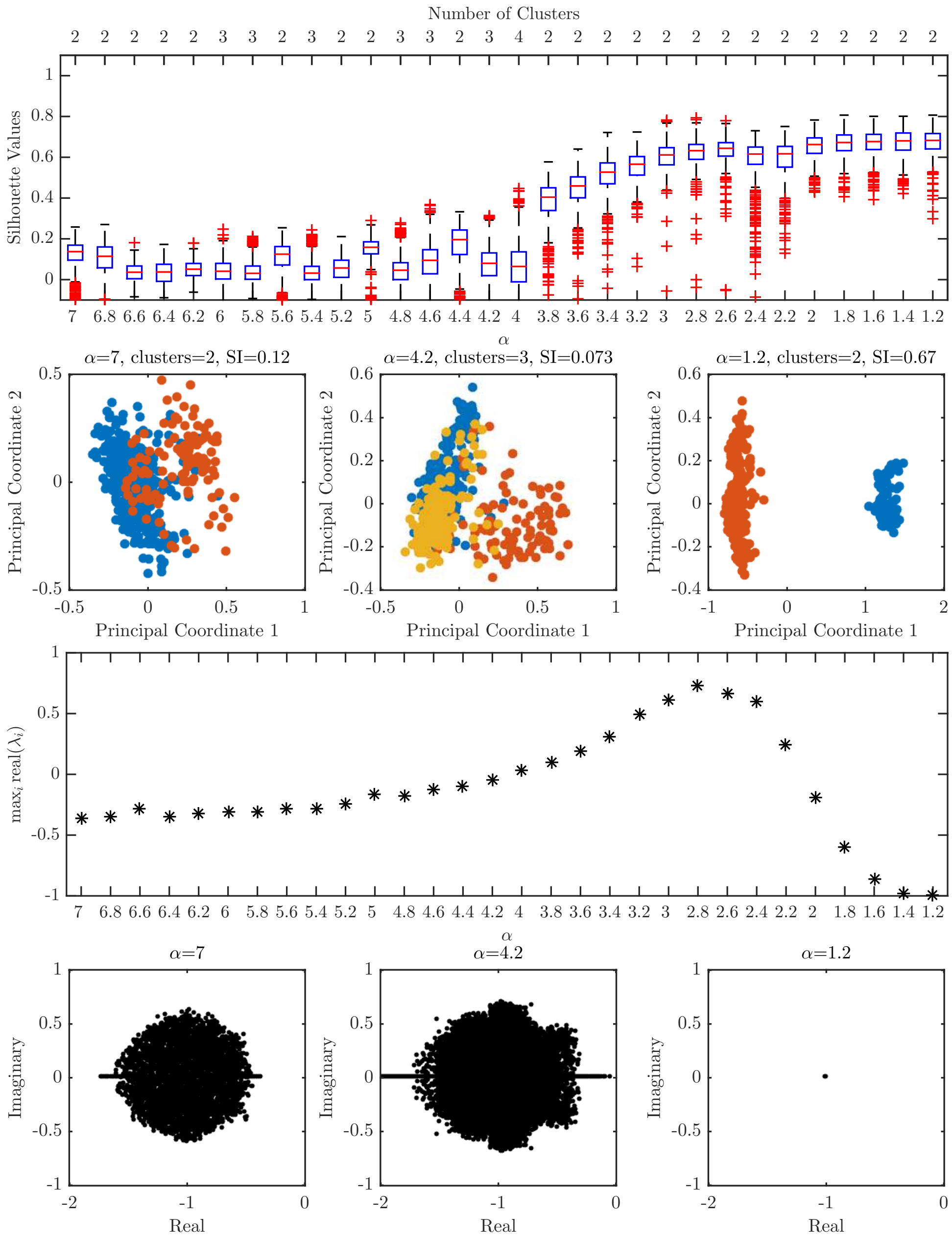

Supplementary Text Figure T28: Universal Model Community Size Overlap Study Scenario 5 in Table T3. The first plot is a comprehensive clustering analysis of the steady state values obtained from the Lotka-Volterra simulations. The x-axis denotes the heterogeneity value α. The box plots are the silhouette values pertaining to the number of clusters for which the silhouette index (maximum over the number of clusters of the mean silhouette value for each given total number of cluster) was defined. The total number of clusters pertaining to the silhouette index is denoted on the top x-axis. The second row is a principle coordinate analysis of the steady state values obtained at three different heterogeneity values. Clusters are color coded to match the optimal clustering from k-medoids. The third row of the figure plots $\max_{i,j}$ real$(\lambda_i(A^{[j]}))$ as a function of α. The fourth row is a plot of $\lambda_i(A^{[j]})$ for $i \in \{1, 2, \ldots, p\}$ and $j \in \{1, 2, \ldots, 500\}$ at three values of α.

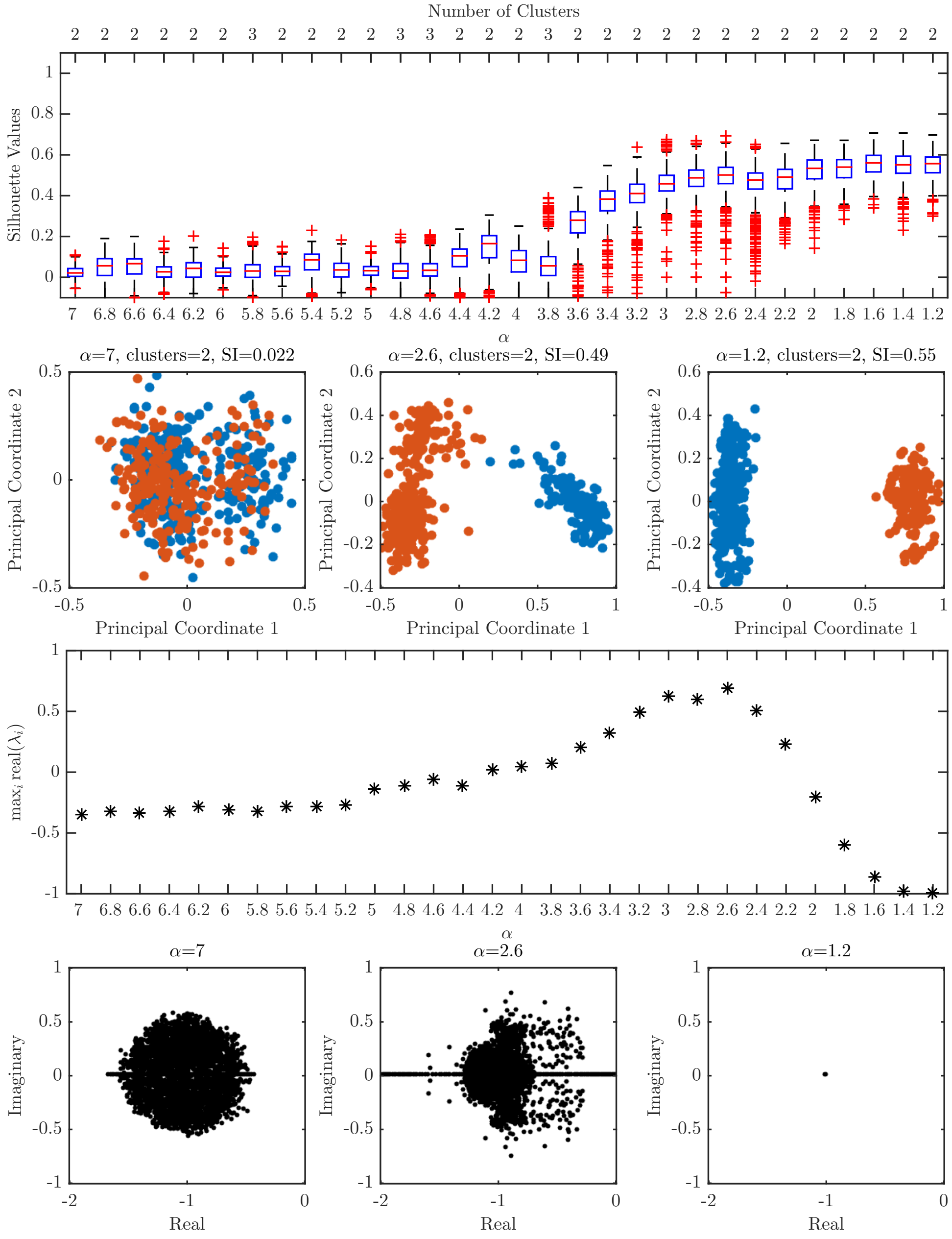

Supplementary Text Figure T29: Universal Model Community Size Overlap Study Scenario 6 in Table T3. The first plot is a comprehensive clustering analysis of the steady state values obtained from the Lotka-Volterra simulations. The x-axis denotes the heterogeneity value α. The box plots are the silhouette values pertaining to the number of clusters for which the silhouette index (maximum over the number of clusters of the mean silhouette value for each given total number of cluster) was defined. The total number of clusters pertaining to the silhouette index is denoted on the top x-axis. The second row is a principle coordinate analysis of the steady state values obtained at three different heterogeneity values. Clusters are color coded to match the optimal clustering from k-medoids. The third row of the figure plots $\max_{i,j} \text{real}(\lambda_i(A^{[j]}))$ as a function of α. The fourth row is a plot of $\lambda_i(A^{[j]})$ for $i \in \{1, 2, \ldots, p\}$ and $j \in \{1, 2, \ldots, 500\}$ at three values of α.

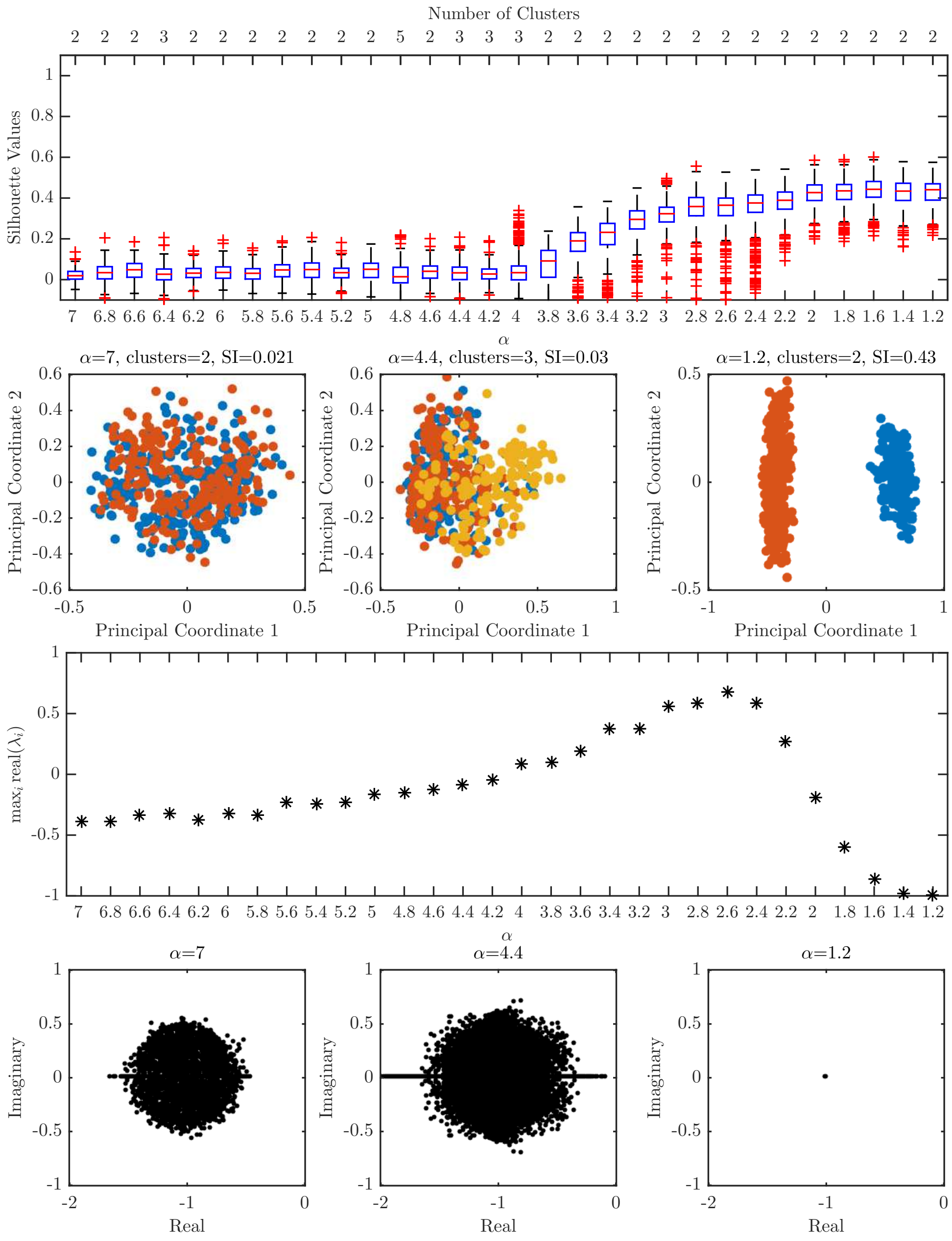

Supplementary Text Figure T30: Universal Model Community Size Overlap Study Scenario 7 in Table T3. The first plot is a comprehensive clustering analysis of the steady state values obtained from the Lotka-Volterra simulations. The x-axis denotes the heterogeneity value α. The box plots are the silhouette values pertaining to the number of clusters for which the silhouette index (maximum over the number of clusters of the mean silhouette value for each given total number of cluster) was defined. The total number of clusters pertaining to the silhouette index is denoted on the top x-axis. The second row is a principle coordinate analysis of the steady state values obtained at three different heterogeneity values. Clusters are color coded to match the optimal clustering from k-medoids. The third row of the figure plots $\max_{i,j} \text{real}(\lambda_i(A^{[j]}))$ as a function of α. The fourth row is a plot of $\lambda_i(A^{[j]})$ for $i \in \{1, 2, \ldots, p\}$ and $j \in \{1, 2, \ldots, 500\}$ at three values of α.

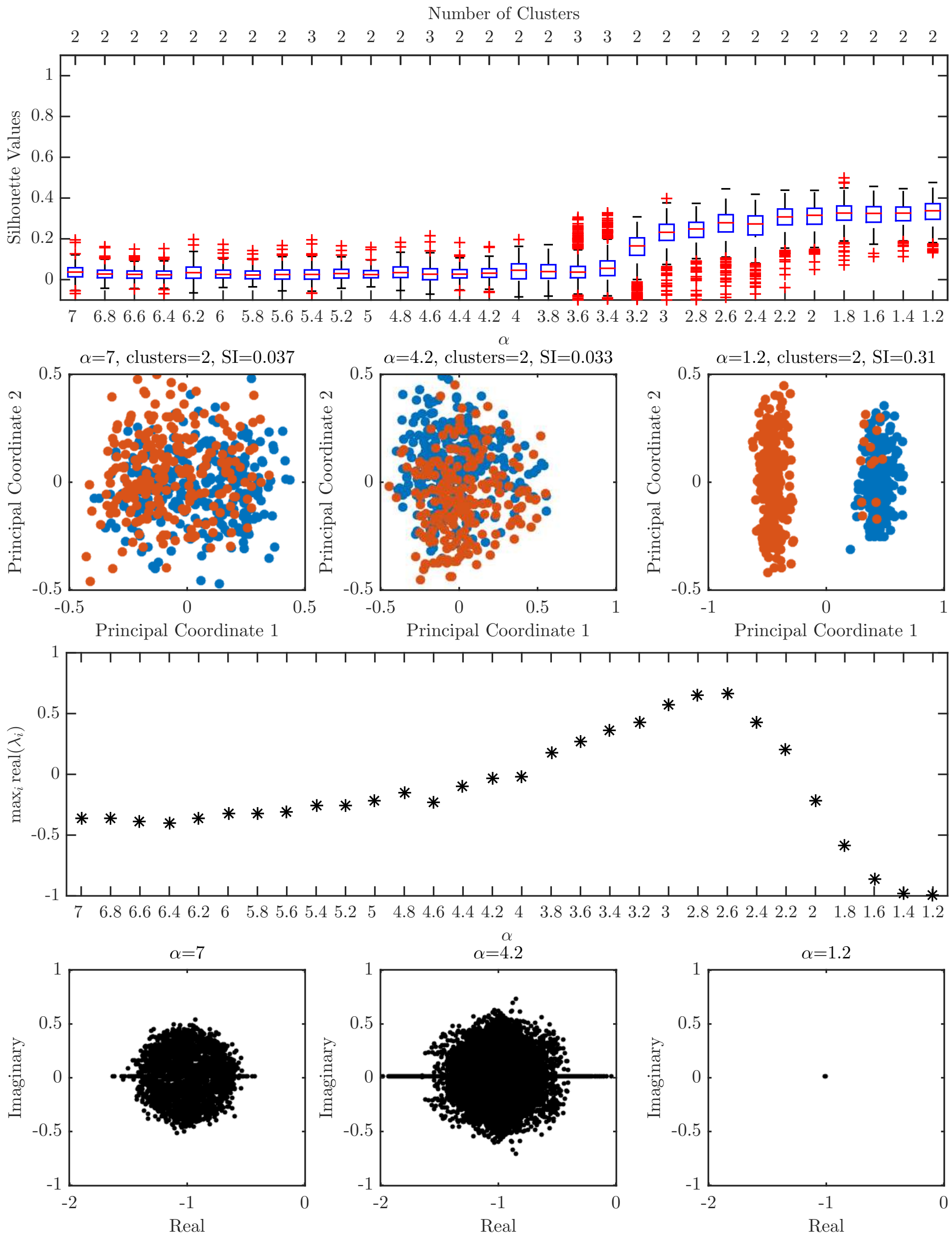

Supplementary Text Figure T31: Universal Model Community Size Overlap Study Scenario 8 in Table T3. The first plot is a comprehensive clustering analysis of the steady state values obtained from the Lotka-Volterra simulations. The x-axis denotes the heterogeneity value α. The box plots are the silhouette values pertaining to the number of clusters for which the silhouette index (maximum over the number of clusters of the mean silhouette value for each given total number of cluster) was defined. The total number of clusters pertaining to the silhouette index is denoted on the top x-axis. The second row is a principle coordinate analysis of the steady state values obtained at three different heterogeneity values. Clusters are color coded to match the optimal clustering from k-medoids. The third row of the figure plots $\max_{i,j}$ real$(\lambda_i(A^{[j]}))$ as a function of α. The fourth row is a plot of $\lambda_i(A^{[j]})$ for $i \in \{1, 2, \ldots, p\}$ and $j \in \{1, 2, \ldots, 500\}$ at three values of α.

Bibliography

[1] ZD Bai, YQ Yin, et al., *Necessary and sufficient conditions for almost sure convergence of the largest eigenvalue of a wigner matrix*, The Annals of Probability **16** (1988), no. 4, 1729–1741.

[2] Béla Bollobás, Christian Borgs, Jennifer Chayes, and Oliver Riordan, *Directed scale-free graphs*, Proceedings of the fourteenth annual acm-siam symposium on discrete algorithms, 2003, pp. 132–139.

[3] Béla Bollobás and Oliver M Riordan, *Mathematical results on scale-free random graphs*, Handbook of graphs and networks: from the genome to the internet (2003), 1–34.

[4] T. Caliński and J Harabasz, *A dendrite method for cluster analysis*, Communications in Statistics **3** (1974), no. 1, 1–27.

[5] A. Clauset, C. Shalizi, and M. Newman, *Power-law distributions in empirical data*, SIAM Review **51** (2009), no. 4, 661–703.

[6] Earl A Coddington and Norman Levinson, *Theory of ordinary differential equations*, Tata McGraw-Hill Education, 1955.

[7] Elizabeth K. Costello, Keaton Stagaman, Les Dethlefsen, Brendan J. M. Bohannan, and David A. Relman, *The application of ecological theory toward an understanding of the human microbiome*, Science **336** (2012), no. 6086, 1255–1262.

[8] G. W. Cross, *Three types of matrix stability*, Linear Algebra and its Applications **20** (19786), no. 3, 253–263.

[9] Hua Deng, Miroslav Krstić, and Ruth J Williams, *Stabilization of stochastic nonlinear systems driven by noise of unknown covariance*, Automatic Control, IEEE Transactions on **46** (2001), no. 8, 1237–1253.

[10] Joseph L Doob, *Stochastic processes*, Whiley, 1990.

[11] ______, *Classical potential theory and its probabilistic counterpart*, Springer Science & Business Media, 2012.

[12] Erdős and Rényi, *On random graphs i*, Publicationes Mathematicae Debrecen **6** (1959), 290–297.

[13] ______, *On the evolution of random graphs*, Publications of the Mathematical Institute of the Hungarian Academy of Sciences **5** (1960), 17–61.

[14] Walter R. Evans, *Control system synthesis by root locus method*, American Institute of Electrical Engineers, Transactions of the **69** (1950), no. 1, 66–69.

[15] E. N. Gilbert, *Random graphs*, The Annals of Mathematical Statistics **30** (1959), no. 4, 1141–1144.

[16] B. S. Goh, *Global stability in many-species systems*, American Naturalist (1977), 135–143.

[17] W. Hahn, *Stability of motion*, Springer–Verlag, New York NY, 1967.

[18] Kiyosi Itô, *Stochastic integral*, Proc. Imperial Acad. Tokyo **20** (1944), 519–524.

[19] Kiyosi Itô, *On a stochastic integral equation*, Proceedings of the Japan Academy **22** (1946), no. 1-4, 32–35.

[20] A. K. Jain, M. N. Murty, and P. J. Flynn, *Data clustering: A review*, ACM Comput. Surv. **31** (1999), no. 3, 264–323.

[21] H. Jeffreys, *The theory of probability*, 2nd ed., Oxford, 1948.

[22] Johan Ludwig William Valdemar Jensen, *Sur les fonctions convexes et les inégalités entre les valeurs moyennes*, Acta Mathematica **30** (1906), no. 1, 175–193.

[23] R. E. Kalman and J. E. Bertram, *Control systems analysis and design via the 'second method' of liapunov, i. continuous-time systems*, Journal of Basic Engineering **82** (1960), 371–393.

[24] Leonard Kaufman and Peter Rousseeuw, *Clustering by means of medoids*, North-Holland, 1987.

[25] Rafail Khasminskii, *Stochastic stability of differential equations*, Vol. 66, Springer Science & Business Media, 2011.

[26] Omry Koren, Dan Knights, Antonio Gonzalez, Levi Waldron, Nicola Segata, Rob Knight, Curtis Huttenhower, and Ruth E. Ley, *A guide to enterotypes across the human body: Meta-analysis of microbial community structures in human microbiome datasets*, PLoS Comput Biol **9** (2013), no. 1.

[27] Solomon Kullback and Richard A Leibler, *On information and sufficiency*, The Annals of Mathematical Statistics (1951), 79–86.

[28] Lun Li, David Alderson, John C Doyle, and Walter Willinger, *Towards a theory of scale-free graphs: Definition, properties, and implications*, Internet Mathematics **2** (2004), no. 4, 431–523.

[29] Jianhua Lin, *Divergence measures based on the shannon entropy*, Information Theory, IEEE Transactions on **37** (1991Jan), no. 1, 145–151.

[30] J. S. Massera, *Contributions to stability theory*, Annals of Mathematics **64** (1956 (Erratum, Ann. of Math, 1958)), no. 1.

[31] Paul André Meyer, *Probability and potentials*, Vol. 7, Blaisdell Publishing Company Waltham (Mass.), 1966.

[32] Glenn W Milligan and Martha C Cooper, *Methodology review: Clustering methods*, Applied psychological measurement **11** (1987), no. 4, 329–354.

[33] K. S. Narendra and A. M. Annaswamy, *Robust adaptive control in the presence of bounded disturbances*, IEEE Trans. Automat. Contr. **31** (1986), 306–315.

[34] ———, *Stable adaptive systems*, Dover, 2005.

[35] William H Press, Brian P Flannery, Saul A Teukolsky, and William T Vetterling, *Numerical recipes: The art of scientific computing*, 3rd ed., Cambridge University Press London, 2007.

[36] Philip E Protter, *Stochastic integration and differential equations*, Vol. 21, Springer, 2013.

[37] George AF Seber, *Multivariate observations*, John Wiley & Sons, 1984.

[38] Robert Shorten and Kumpati S Narendra, *On a theorem of redheffer concerning diagonal stability*, Linear Algebra and its Applications **431** (2009), no. 12, 2317–2329.

[39] Daniel W Stroock, *Probability theory: an analytic view*, Cambridge university press, 2010.

[40] T. Tao and V. Vu, *Random matrices: The Universality phenomenon for Wigner ensembles*, ArXiv (January 2012), available at http://arxiv.org/abs/1202.0068.

[41] Terence Tao, *An introduction to measure theory*, Vol. 126, American Mathematical Soc., 2011.

[42] ———, *Topics in random matrix theory*, Vol. 132, American Mathematical Soc., 2012.

[43] Terence Tao, Van Vu, and Manjunath Krishnapur, *Random matrices: Universality of esds and the circular law*, The Annals of Probability **38** (201009), no. 5, 2023–2065.

[44] Eugene P. Wigner, *Characteristic vectors of bordered matrices with infinite dimensions*, Annals of Mathematics **62** (1955/11/01), no. 3, 548–564.

[45] ———, *Characteristics vectors of bordered matrices with infinite dimensions ii*, Annals of Mathematics **65** (1957/03/01), no. 2, 203–207.

[46] ———, *On the distribution of the roots of certain symmetric matrices*, Annals of Mathematics **67** (1958/03/01), no. 2, 325–327.
